# Supplementary material for: Genome mining for macrolactam-encoding gene clusters allowed for the network-guided isolation of β-amino acid-containing cyclic derivatives and heterologous production of ciromicin A
Source: Commun Chem. 2023 Nov 20;6:257. doi: 10.1038/s42004-023-01034-w (PMC10662134; doi:10.1038/s42004-023-01034-w)
Supplement: Supplementary file 2 — Supplementary Information [file 42004_2023_1034_MOESM2_ESM.pdf]

Supplementary Items

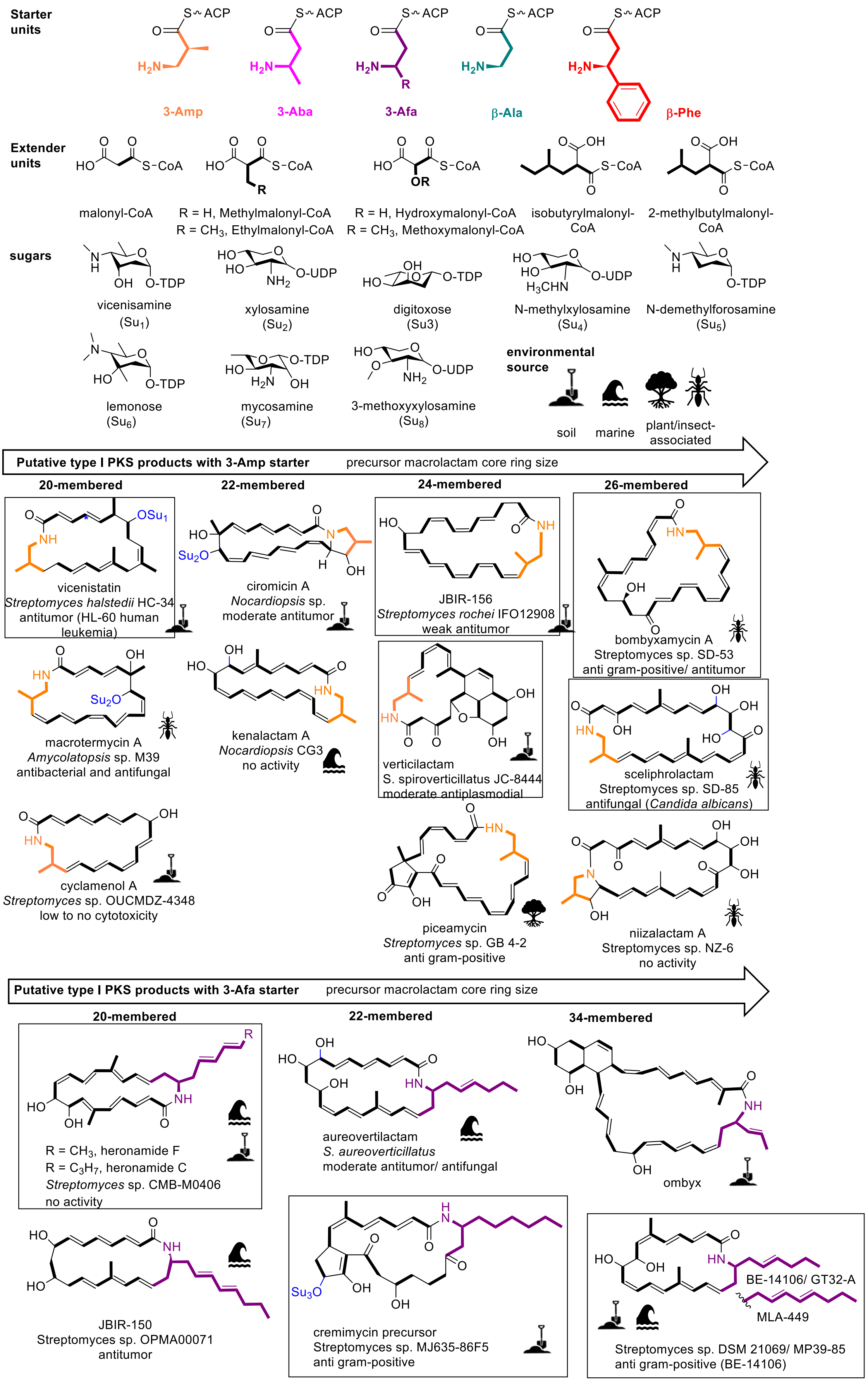

Figure S1. Comparative summary of known natural polyene macrolactams organized by starter unit and precursor ring size (part I). For compounds in boxes the biosynthesis gene cluster was already verified, for all others only predicted. Pictograms show microbial isolation source**.**

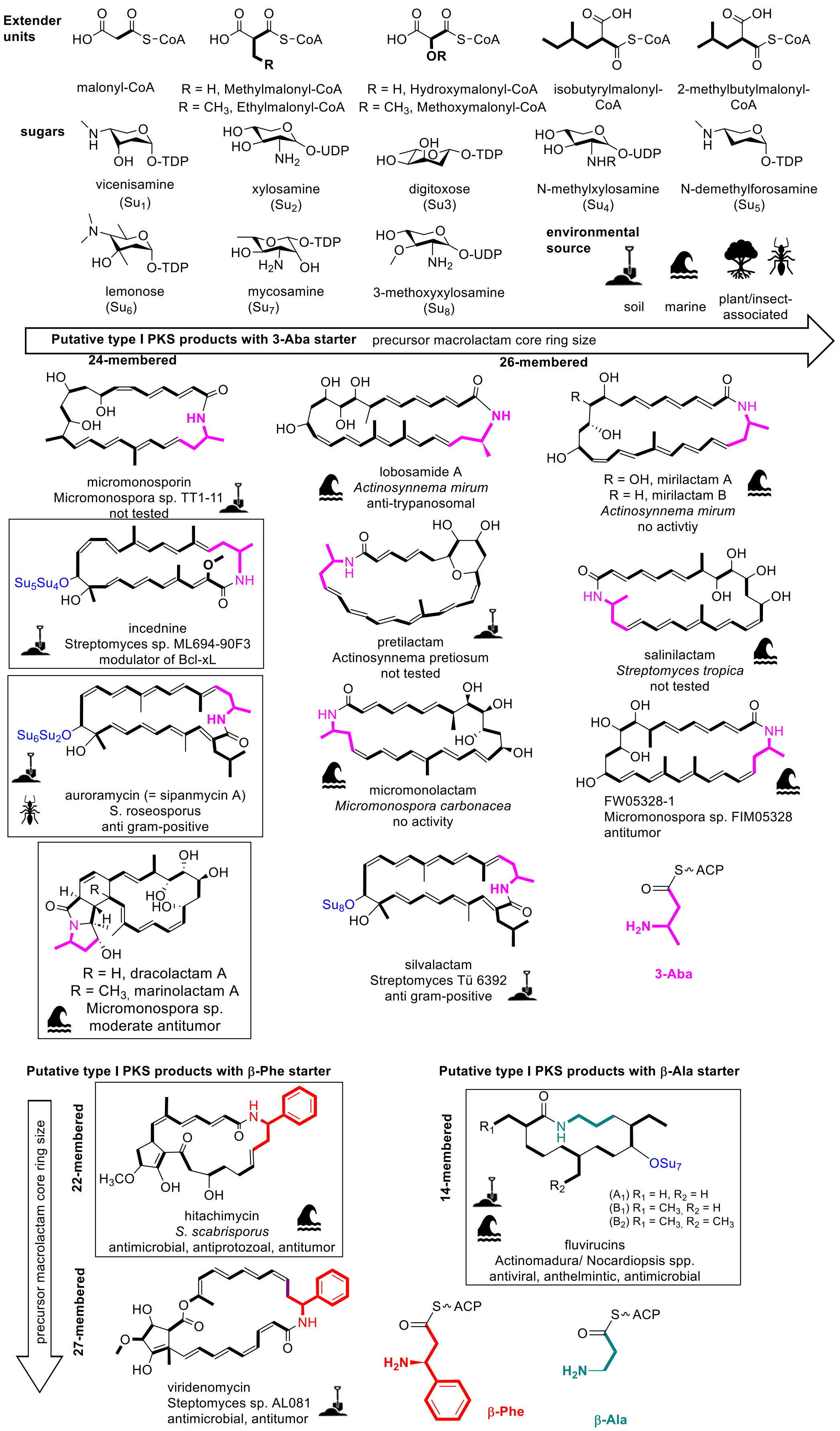

Figure S2. Comparative summary of known natural polyene macrolactams organised by starter unit and precursor ring size (part II). For compounds in boxes the biosynthesis gene cluster was already verified, for all others only predicted. Pictograms show microbial isolation source.

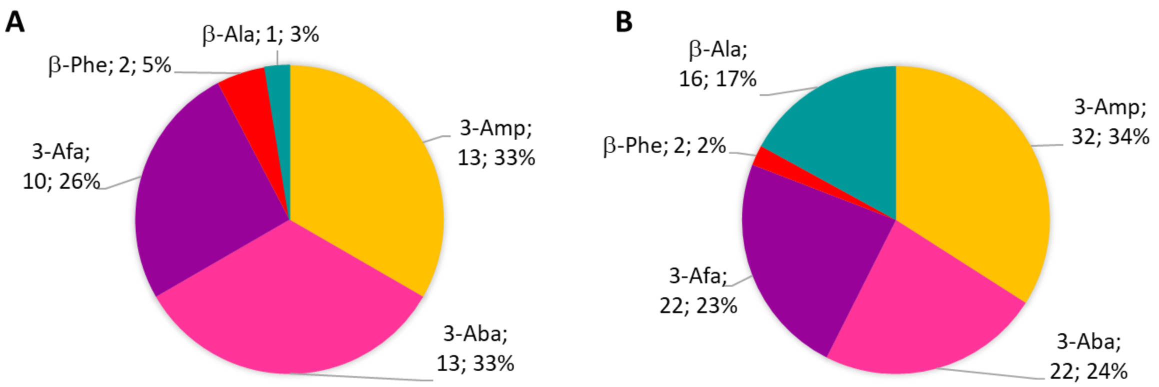

Figure S3. Distribution and classification of isolated macrolactams by amino acid starter unit. A: Distribution of individual isolated macrolactam families (collection of all derivatives sharing the same name) by starter unit. B: Distribution of individual compounds (all derivatives within one family) by starter unit.

Table S1. Query sequences and parameters used in cblaster homology search.

BGC type	Query BGC	Query sequences (number of query sequences [N])	cblaster search parameters
3-Amp	vicenistatin	VinH, VinI, VinK, VinL, VinM, VinN, VinO, IdnLP1 (8)	-ig -md 70000 -g 80000 -r VinK -u 6
β-Ala	vicenistatin	VinH*, VinI*, VinK, VinL, VinM, VinN, VinO, IdnLP1 (8)	-ig -md 70000 -g 80000 -r VinK VinO -u 4
3-Aba	incednine	IdnL1, IdnL2, IdnL3, IdnL4, IdnL6, IdnL7m IdnLP1 (7)	-ig -md 70000 -g 80000 -r IdnL2, IdnL3, IdnL4, -u 5
3-Afa	cremimycin	CmiS1, CmiS2, CmiS3, CmiS4, CmiS5, CmiS6, IdnLP1 (7)	-ig -md 70000 -g 80000 -r CmiS1 CmiS2
β-Phe	hitachimycin	HitA, HitB, HitC, HitD, HitE, IdnLP1 (6)	-ig -md 70000 -g 80000 -r hitA -u4

* The minimum number if total hits (-u) was set to N-4, as these enzymes are expected to be absent in β-Ala clusters.

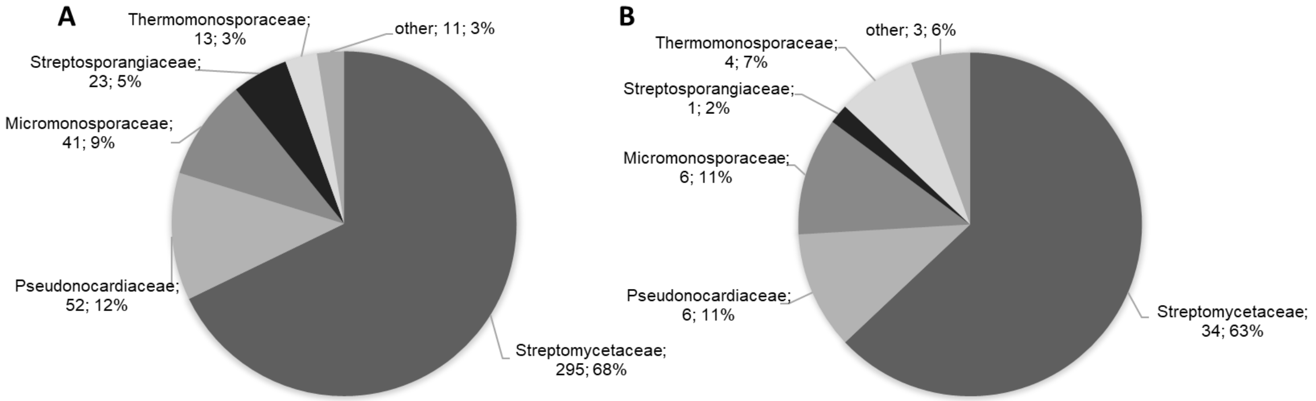

Figure S4. Comparison of the relative distribution of predicted BGCs and isolated macrolactam compounds in bacterial families. (A) Distribution of macrolactam BGCs in bacterial families. Macrolactam BGCs were predicted in genome-sequenced bacteria by cblaster homology search[1] using a set of specific query sequences for each macrolactam BGC type. **(B)** Distribution of isolated macrolactam compounds across bacterial families. Other families include Frankiaceae, Nocardiaceae, Nocardiopsaceae, Phyllobacteriaceae and Catenulisporaceae.

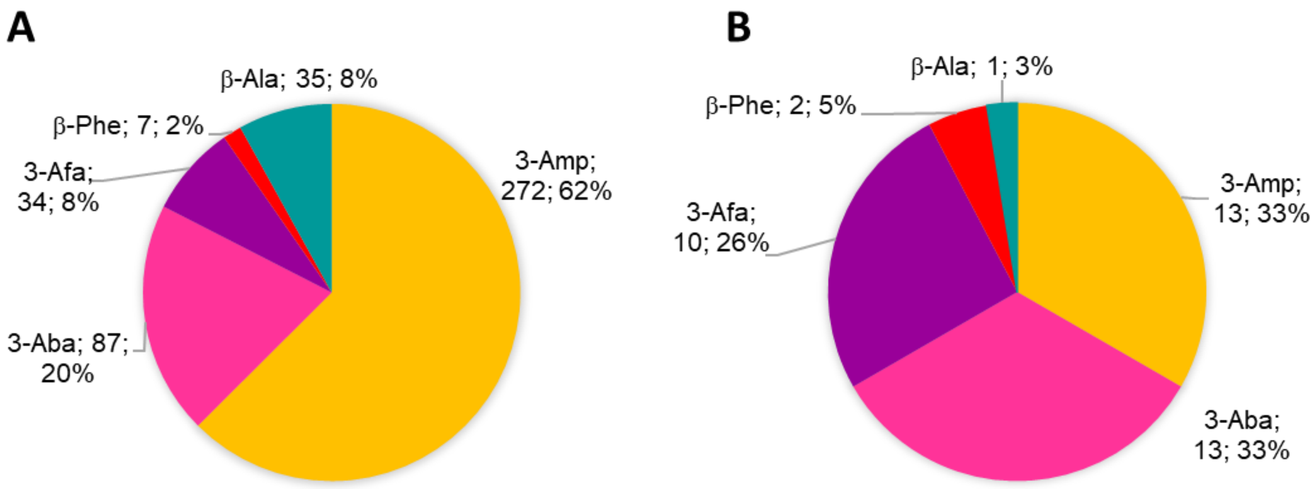

Figure S5. Comparison of the relative distribution of different macrolactam BGC types on genomic and compound level. (A) Relative distribution of predicted macrolactam BGCs with β-alanine (β-Ala, green), β-phenylalanine (β-Phe, red), 3-amino fatty acid (3-Afa, purple), 2-aminobutyrate (3-Aba, pink) and 3-aminomethylpropionate (3-Amp, yellow) starter unit. **(B)** Relative abundance of the different starter units among isolated macrolactams.

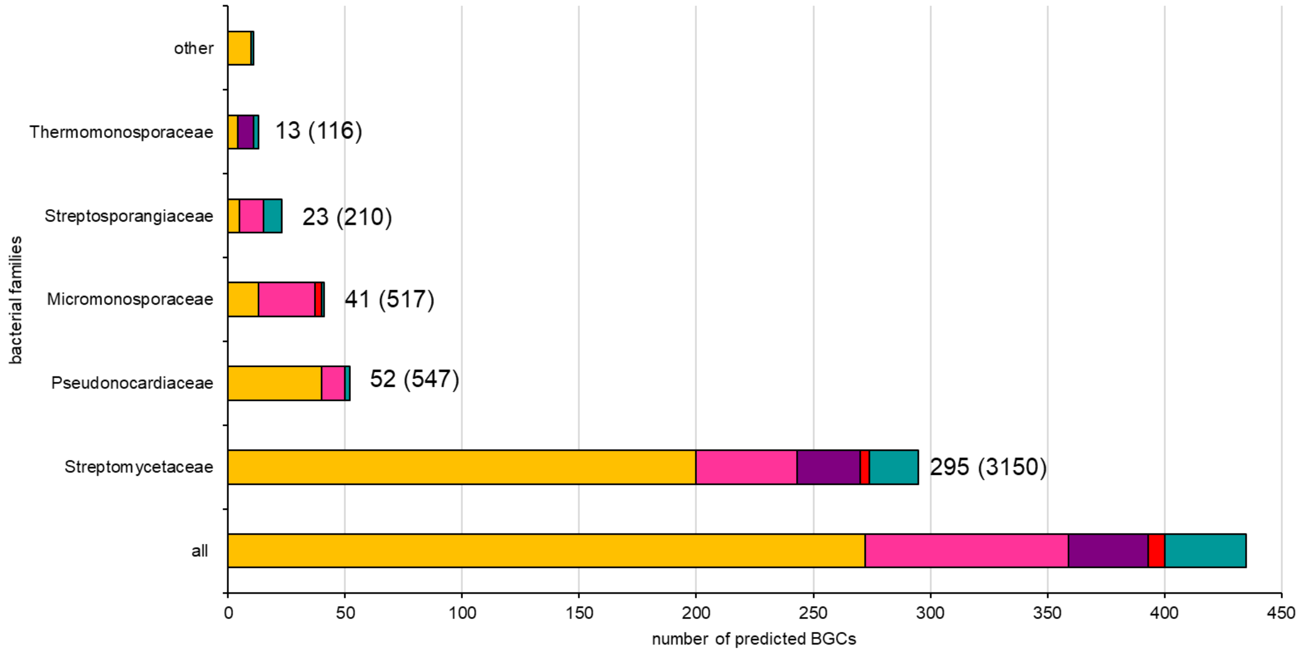

Figure S6. Distribution of different macrolactam BGC types among bacterial families. Depicted are the abundances of macrolactam BGCs with β-alanine (β-Ala, green), β-phenylalanine (β-Phe, red), 3-amino fatty acid (3-Afa, purple), 2-aminobutyrate (3-Aba, pink) and 3-aminomethylpropionate (3-Amp, yellow) starter unit among genome-sequenced bacterial families. All represents the sum of all detected clusters of each BGC type. Other families include Frankiaceae, Nocardiaceae, Nocardiopsaceae, Phyllobacteriaceae and Catenulisporaceae. The number in brackets indicates the number of available genomes of bacterial families in the NCBI database, retrieved on 27.03.2023.

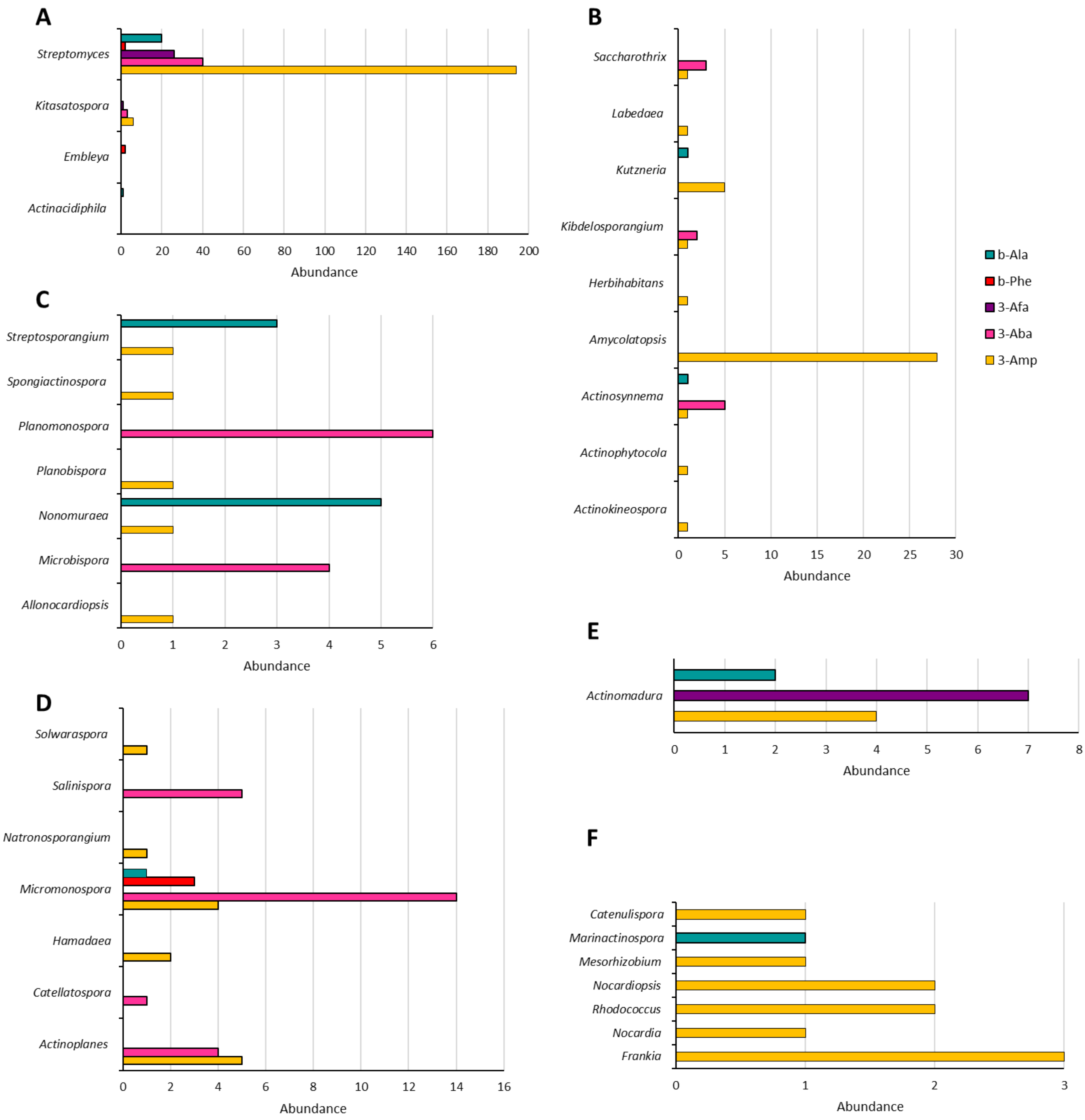

Figure S7. Distribution of macrolactam BGC among bacterial genera. Depicted are the abundances of macrolactam BGCs with β-alanine (β-Ala, green), β-phenylalanine (β-Phe, red), 3-amino fatty acid (3-Afa, purple), 2-aminobutyrate (3-Aba, pink) and 3-aminomethylpropionate (3-Amp, yellow) starter unit among genome-sequenced genera within the families of Streptomycetaceae **(A)**, Pseudonocardiaceae **(B)**, Streptosporangiaceae **(C)**, Micromonosporaceae **(D)**, Thermomonosporaceae **(E)** and others **(F)**.

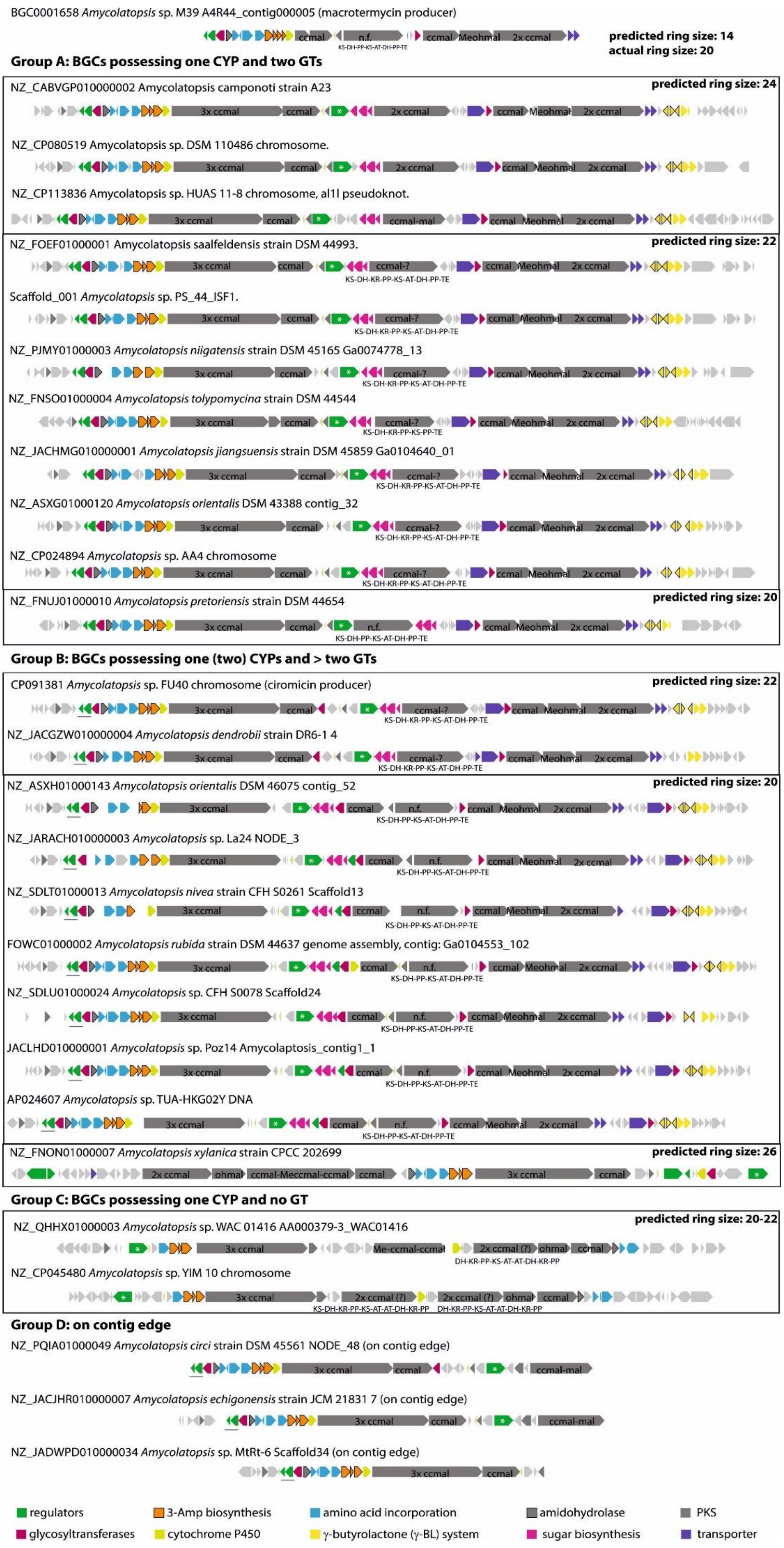

Figure S8. Cluster architecture of predicted macrolactam BGCs detected in *Amycolatopsis* by cblaster homology search. Clusters were visualized using multigeneblast. ccmal – partially reduced malonate resulting from ketoreduction (KR) and dehydration (DH), ohmal - partially reduced malonate resulting from ketoreduction (KR), Meccmal – partially reduced methyl malonate resulting from ketoreduction (KR) and dehydration (DH), Meohmal- partially reduced methyl malonate resulting from ketoreduction (KR), n. f. – putatively not functional, (?) – unclear module composition (missing/additional domains). * highlights LuxR regulator-encoding genes.

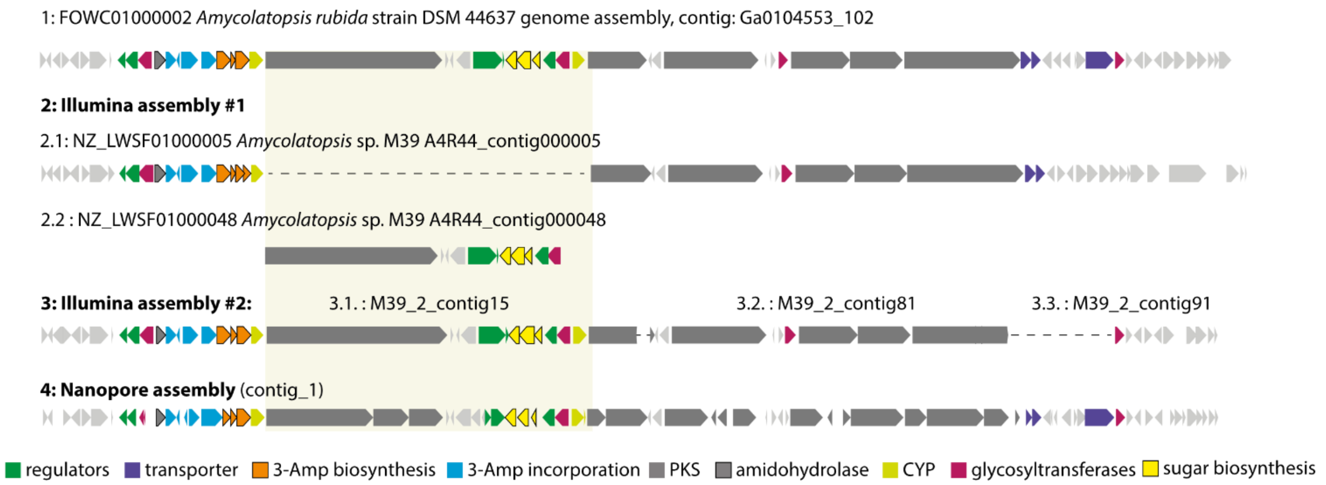

Figure S9. Comparison of the macrotermycin cluster in different genome assemblies. Short-read (Illumina) and long-read (nanopore) sequencing-based genome assemblies revealed the location of the additional cluster region missing in the published macrotermycin gene cluster and shows the high similarity to the BGC in *Amycolatopsis rubida* DSM 44637.

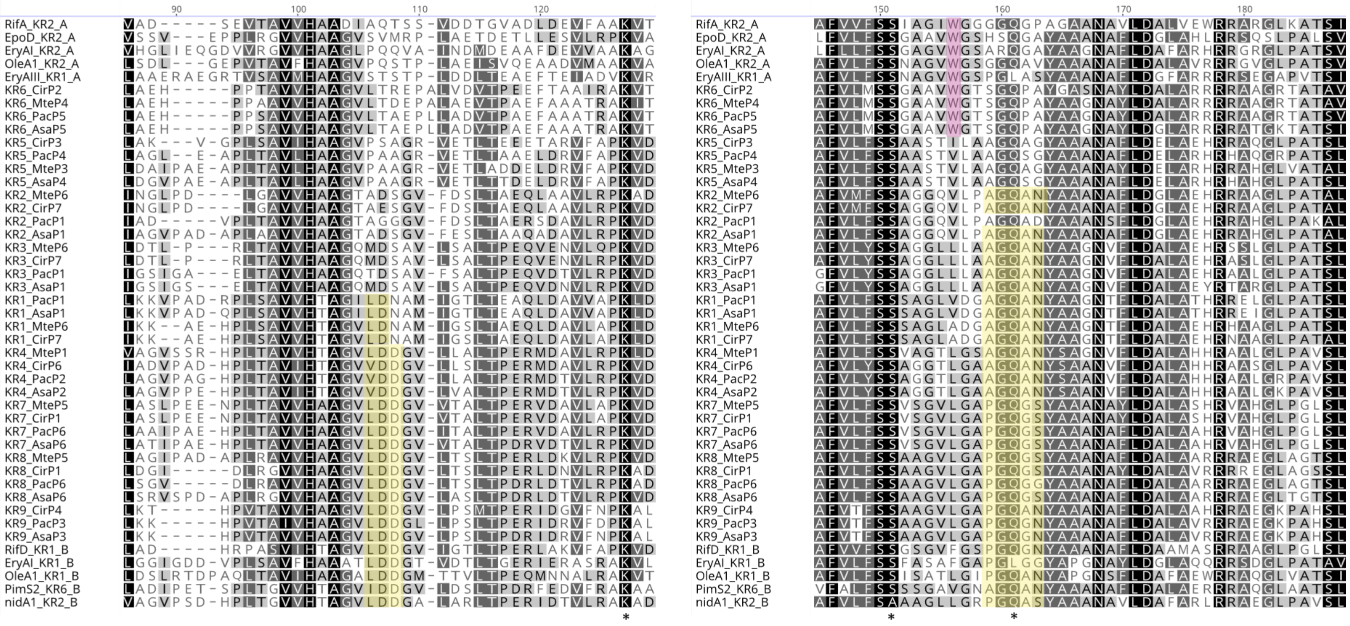

Figure S10. Analysis of ketoreductase stereochemistry. KR domains from *cir*, *mte*, *asa* and *pac* BGCs were aligned with A- and B-type ketoreductases from Caffrey et al. (2003).[2] The LDD motif and P144/N148 (in this alignment P159/N1163) typical for B-type ketoreductases and the characteristic W141 (here W156) are highlighted in yellow and pink, respectively. Asterisks (*) mark catalytic residues. NCBI accession numbers are: MteP1b (OAP25812.1), MteP2 (OAP25815.1), MteP3 (OAP25819.1), MteP4 (OAP25820.1), MteP6 (OAP25821.1), AsaP1 (WP_218156647.1), AsaP2 (SEO46972.1), AsaP3 (WP_091610894.1), AsaP4 (WP_091610879.1), AsaP5 (WP_091612723.1), AsaP6 (WP_218156646.1), CirP1 (UKD51442.1), CirP2 (UKD51443.1), CirP3 (UKD51444.1), CirP4 (UKD51451.1), CirP6 (UKD51462.1), CirP7 (UKD51463.1), RifA (AAC01710.1), EpoD (ADB12491.1), EryAI (AAU93807.2), OleAI (AAF82408.1), EryAIII (AAU93805.2), RifD (AAC01713.1), PimS2 (CAC20921.1), NidA1 (AAC46024.1). Rif – rifamycin, Epo – epothilone, Ery – erythromycin, Ole – oleandomycin, Pim – pimaricin, Nid – niddamycin, Mte – macrotermycin, Cir – ciromicin from *Amycolatopsis* sp. FU40, Asa – ciromicin from *A. saalfeldensis*, Pac – ciromicin from *Amycolatopsis* sp. PS_44_ISF1.

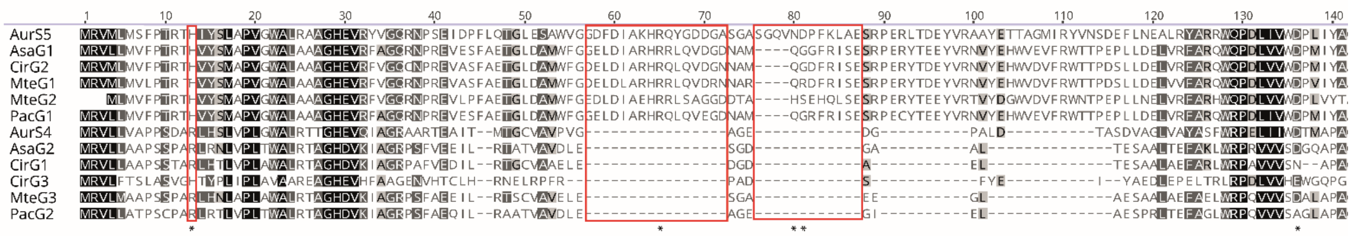

Figure S11. Alignment of glycosyltransferases (extracted region). Putative residues, based on AurS4 and AurS5 (Yeo et al.[3]), involved in acceptor and donor nucleotide binding are highlighted (*). Red boxes indicate distinctions within truncated glycosyltransferases AurS4 and homologs.

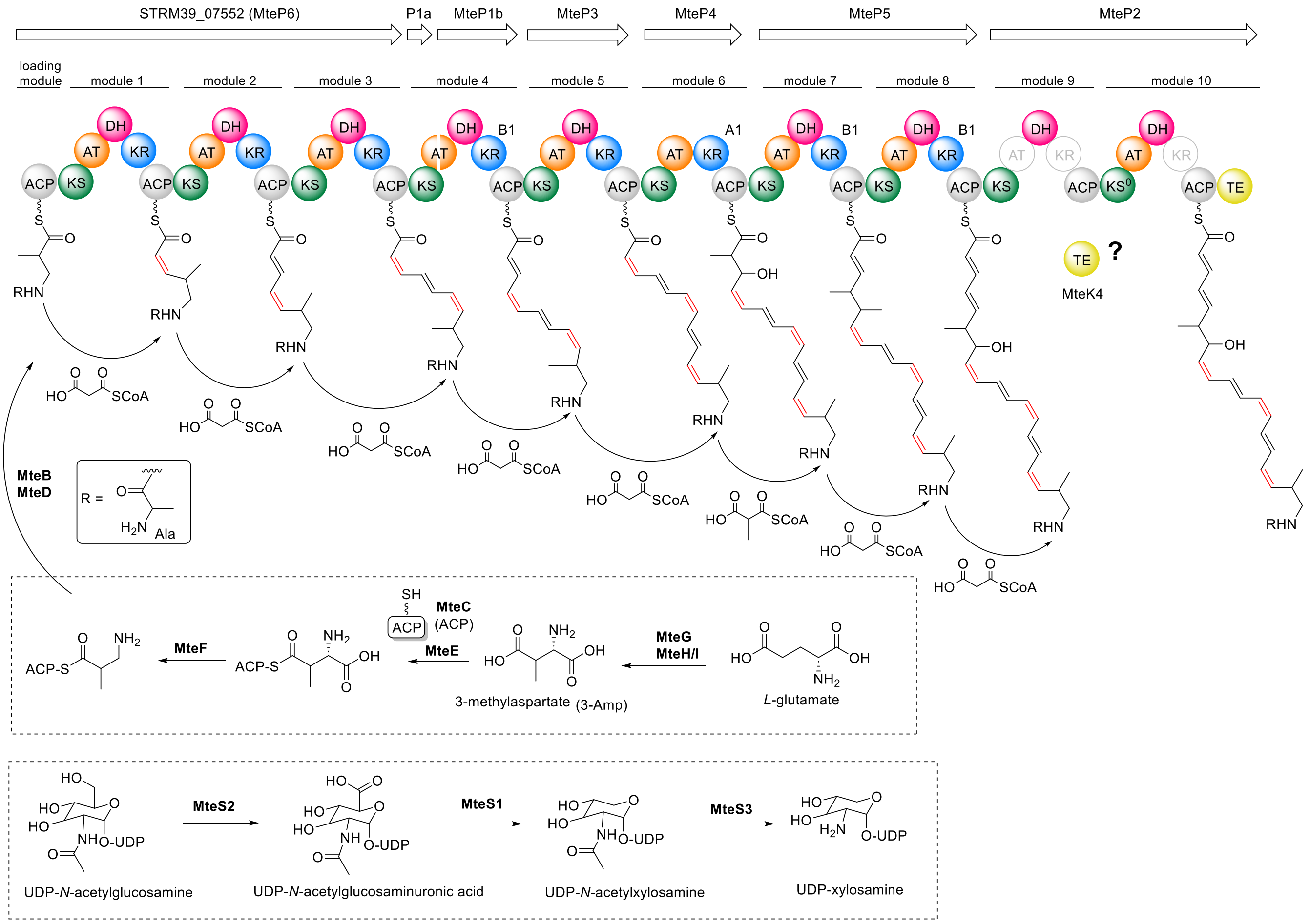

Figure S12. Revised biosynthetic pathway proposal for macrolactam biosynthesis in *Amycolatopsis* sp. M39.

Table S2. Proteins encoded in the *pac* BGC and comparison to NCBI and MiBIG database (MiBIG version 2.0, date of access to NCBI: 15.08.2022).

					Closest homolog from macrolactam BGC (>50% identity)		
Name	aa	Sequence Similarity (Protein, Origin) NCBI/MiBIG	Ident./ Sim. [%]	NCBI/ MiBIG Acc. No.	*asa*	*cir*	*mte*
PacR1	217	NCBI: response regulator transcription factor [Amycolatopsis saalfeldensis]<br>MiBIG: Transcriptional regulatory protein DegU [*Amycolatopsis sp. M39*, macrotermycin]	98/100<br>95	WP_177231118.1<br>OAP25798.1	R1	R8	R1
PacR2	376	NCBI: sensor histidine kinase [Amycolatopsis saalfeldensis]<br>MiBIG: Sensor_histidine_kinase_LiaS [Amycolatopsis sp. M39, macrotermycin]	88/92<br>79	WP_091610949.1<br>OAP25799.1	R2	R7	R2
PacG1	420	NCBI: glycosyltransferase, activator-dependent family [*Amycolatopsis saalfeldensis*]<br>MiBIG: Desosaminyl transferase EryCIII precursor [*Amycolatopsis sp. M39*, macrotermycin]	95/96<br>79	WP_091610946.1<br>OAP25800.1	G1	G2	G1
PacA	319	NCBI: L-proline amide hydrolase [*Amycolatopsis saalfeldensis*]<br>MiBIG: L-amino_acid_amidase [*Amycolatopsis sp. M39*, macrotermycin]	89/90<br>81	SEO47314.1<br>OAP25801.1	A	X2	A
PacB	319	NCBI: ACP S-malonyltransferase [Amycolatopsis saalfeldensis]<br>MiBIG: Malonyl_CoA-acyl_carrier_protein_transacylase [Amycolatopsis sp. M39, macrotermycin]	97/99<br>89	WP_091610940.1<br>OAP25802.1	B	A4	B
PacC	81	NCBI: Acyl carrier protein [Amycolatopsis saalfeldensis]<br>MiBIG: Acyl_carrier_protein[Amycolatopsis sp. M39, macrotermycin]	98/98<br>84	SEO47253.1<br>OAP25803.1	C	A7	C
PacD	507	NCBI: AMP-binding protein [Amycolatopsis saalfeldensis]<br>MiBIG: Surfactin_synthase_subunit_1 [Amycolatopsis sp. M39, macrotermycin]	93/95<br>83	WP_091610934.1<br>OAP25804.1	D	A6	D
PacE	492	NCBI: AMP-binding protein [Amycolatopsis saalfeldensis]<br>MiBIG: Long-chain-fatty-acid--CoA_ligase[Amycolatopsis sp. M39, macrotermycin]	93/95<br>86	WP_245787070.1<br>OAP25805.1	F	A5	E
PacF	404	NCBI: decarboxylase [Amycolatopsis saalfeldensis]<br>MiBIG: L-glutamyl-[BtrI_acyl-carrier_protein]_decarboxylase[Amycolatopsis sp. M39, macrotermycin]	90/93<br>83	WP_091610927.1<br>OAP25806.1	G	A3	F
PacG	158	NCBI: methylaspartate mutase sigma subunit [Amycolatopsis saalfeldensis]<br>MiBIG: Methylaspartate_mutase_S_chain[Amycolatopsis sp. M39, macrotermycin]	91/94<br>75	WP_091610925.1<br>OAP25807.1	H	A2	G
PacH	424	NCBI: methylaspartate mutase [Amycolatopsis saalfeldensis]<br>MiBIG: Methylaspartate_mutase_E_chain_(MutE) [Amycolatopsis sp. M39, macrotermycin]	95/97<br>79	WP_091610922.1<br>OAP25809.1	I	A1	H/I
PacO1	398	NCBI: cytochrome P450 [Amycolatopsis saalfeldensis]<br>MiBIG: Cytochrome_P450_107B1 [Amycolatopsis sp. M39, macrotermycin]	94/97<br>84	WP_091610919.1<br>OAP25810.1	O1	O1	O1/2
PacP1	5370	PKS (ACP-KS-AT-DH-KR-ACP-KS-AT-DH-KR-ACP-KS-AT-DH-KR-ACP)			P1	P7	P1
PacP2	1797	PKS (KS-AT-DH-KR-ACP-Cterm_Docking)			P2	P6	P2a/b
ctg1_2610	89	NCBI: hypothetical protein SAMN04489732_101127 [Amycolatopsis saalfeldensis]<br>MiBIG: hypothetical_protein[Amycolatopsis sp. M39, macrotermycin]	91/97<br>75	SEO46938.1<br>OAP25813.1	BMY97_RS00610	U6	A4R44_RS50295
PacI	255	NCBI: Surfactin synthase thioesterase subunit [Amycolatopsis saalfeldensis]<br>MiBIG: Linear_gramicidin_dehydrogenase_LgrE[Amycolatopsis sp. M39, macrotermycin]	84/89<br>71	SEO46909.1<br>OAP25814.1	I	P5	I
PacR3	911	NCBI: regulatory protein, luxR family [Amycolatopsis saalfeldensis]	86/89<br>44	SEO46875.1<br>BAP34706.1	R3	R5	R4

		MiBIG: LuxR-family_transcriptional_regulator [IdnR1, *Streptomyces sp. ML694-90F3, incednine]*					
PacR4	50	NCBI: helix-turn-helix transcriptional regulator [Amycolatopsis saalfeldensis]	72/76	WP_177231117.1	R4	U4	R5
PacS1	319	NCBI: GDP-mannose 4,6-dehydratase [Amycolatopsis saalfeldensis]<br>MiBIG: NAD-dependent_epimerase/dehydratase [idnS3, , *Streptomyces sp. ML694-90F3, incednine]*	93/96<br>74	WP_091610901.1<br>BAP34705.1	S1	S3	S1
PacS2	430	NCBI: nucleotide sugar dehydrogenase [Amycolatopsis saalfeldensis]<br>MiBIG: UDP-glucose/GDP-mannose_dehydrogenase (idnS2, *Streptomyces sp. ML694-90F3, incednine)*	94/97<br>68	WP_091610900.1<br>BAP34704.1	S2	S2	S2
PacS3	227	NCBI: N-acetylglucosaminyl deacetylase, LmbE family [Amycolatopsis saalfeldensis]<br>MiBIG: N-acetylglucosaminyl_deacetilase [*Streptomyces sp. CS149, sipanmycin]*	94/96<br>73	SEO46758.1<br>sipS1	S3	S1	S3
PacP3	3246	PKS (KS-AT-DH-KR-ACP-KS-AT-DH-ACP-TE)			P3	P4	P3
PacR5	241	NCBI: TetR/AcrR family transcriptional regulator [Amycolatopsis saalfeldensis]<br>MiBIG: putative_TetR_family_transcriptional_regulator [*Streptomyces filamentosus, auroramycin]*	84/88<br>33	WP_091610892.1<br>AWR88400.1	R5	R4	-
**ctg1_2619**	139	NCBI: hypothetical protein [Amycolatopsis saalfeldensis]<br>MiBIG: hypothetical_protein [Amycolatopsis sp. M39, macrotermycin]	87/93<br>67	WP_091610890.1<br>OAP25817.1	BMY97_RS00565	U2	-
**ctg1_2620**	78	NCBI: hypothetical protein [Amycolatopsis saalfeldensis]	88/93	WP_091610887.1	BMY97_RS00560	U1	-
PacT1	255	NCBI: putative ABC transport system ATP-binding protein [Amycolatopsis saalfeldensis]<br>MiBIG: ABC_transporter [*Micromonospora sp. RL09-050-HVF-A, lobosamide]*	85/99<br>64	SEO46596.1<br>ALA09377.1	T1	T4	-
PacT2	854	NCBI: ABC transporter permease [Amycolatopsis saalfeldensis]<br>MiBIG: ABC_transporter [*Micromonospora sp. RL09-050-HVF-A, lobosamide]*	91/94<br>41	WP_091610885.1<br>ALA09378.1	T2	T3	-
PacG2	303	NCBI: DUF1205 domain-containing protein [Amycolatopsis saalfeldensis]<br>MiBIG: Desosaminyl_transferase_EryCIII_precursor[Amycolatopsis sp. M39, macrotermycin]	77/83<br>66	WP_177231116.1<br>OAP25818.1	G2	G1	G3
PacP4	1743	PKS (KS-AT-DH-KR-ACP)			P4	P3	P4
PacP5	1591	PKS (KS-AT-KR-ACP)			P5	P2	P5
PacP6	3472	PKS (KS-AT-DH-KR-ACP-KS-AT-DH-KR-ACP)			P6	P1	P6
PacT3	308	NCBI: ABC-2 type transport system ATP-binding protein [Amycolatopsis saalfeldensis]<br>MiBIG:Daunorubicin/doxorubicin_resistance_ATP-binding_protein_DrrA [Amycolatopsis sp. M39, macrotermycin]	94/95<br>88	SEO46383.1<br>OAP25822.1	T3	T2	T1
PacT4	275	NCBI: ABC transporter permease [Amycolatopsis saalfeldensis]<br>MiBIG: Daunorubicin/doxorubicin_resistance_ABC_transporter_permease_protein_DrrB [Amycolatopsis sp. M39, macrotermycin]	95/96<br>83	WP_091610874.1<br>OAP25823.1	T4	T1	T2
ctg1_2629	200	NCBI: hypothetical protein [Amycolatopsis saalfeldensis]<br>MiBIG: CcbJ, methyltransferase [*Streptomyces caelestis, celesticetin]*	76/80<br>34	WP_091610872.1<br>ADB92558.1	BMY97_RS00510	M1	-
PacR6	254	NCBI: AfsR/SARP family transcriptional regulator [Amycolatopsis saalfeldensis]	96/98<br>50	WP_091610869.1<br>AFI57010.1	R6	R3	R7

		MiBIG: Regulator [*Amycolatopsis orientalis, quartromicin A1]*					
PacR7	205	NCBI: TetR/AcrR family transcriptional regulator [Amycolatopsis saalfeldensis]<br>MiBIG: butyrolactone_receptor [*Streptomyces carzinostaticus subsp. Neocarzinostaticus,* neocarzinostatin]	76/83<br>35	WP_091610867.1<br>AAM78022.1	R7	R2	R8
PacR8	205	NCBI: DNA-binding transcriptional regulator, AcrR family [Amycolatopsis saalfeldensis]<br>MiBIG: PgaR2, TetR family transcriptional regulator [*Streptomyces sp. PGA64, prejadomycin]*	86/92<br>42	SEO46237.1<br>AHW57765.1	R8	R1	R9
PacY1	322	NCBI: A-factor biosynthesis hotdog domain-containing protein [Amycolatopsis saalfeldensis]<br>MiBIG: Lct9, butyrolactone: AfsA [*Streptomyces rishiriensis, lactonamycin]*	72/81<br>39	SEO46211.1<br>ABX71092.1	Y1	Y1	Y1
PacY2	341	NCBI: NAD-dependent epimerase/dehydratase family protein [Amycolatopsis saalfeldensis]<br>MiBIG: NDP-hexose-2,3-dehydratase [*Streptomyces carzinostaticus subsp. Neocarzinostaticus,* neocarzinostatin]	76/80<br>39	WP_091610859.1<br>AAM78021.1	Y2	Y2	Y2

Table S3. Recipes of media used in this study.

Medium	Composition (per L)	Purpose
MS broth/ agar	20 g D(-) mannitol, 20 g soybean flour (Sigma-Aldrich), for agar: 2% agar (w/v)	Standard cultivation/ conjugation metabolite production medium
ISP2 broth/ agar	4 g yeast extract, 10 g malt extract, 4 g dextrose, pH 7.2, for agar: 2% agar (w/v)	metabolite production medium
LB broth/ agar	25 g LB (Luria/Miller), for agar: 1.5% agar (w/v)	Standard cultivation of *E. coli*
2x YT broth	16 g Bacto Tryptone, 10 g yeast extract, 5 g NaCl, pH 7.0	Resurrection of *Streptomyces* spores
PDB/ PDA	26.5 g potato extract glucose broth, for PDA: 2% agar (w/v)	metabolite production medium/ transcriptome analysis/ co-cultivation assays
DNPM broth/agar	40 g dextrin, 7.5 g peptone from soy, 5 g yeast extract, 21 g MOPS, pH 6.8	metabolite production medium
VM broth/ agar	5 g glucose, 5 g glycerol, 1.3 g glutamate, 5 g soybean flour, 1 g CaCO3, pH 7.4	metabolite production medium

Table S4. Strains used in this study.

Strain	Purpose, genotype	Abbreviated name	Reference
Amycolatopsis sp. M39	wild type (isolated from the gut of *Macrotermes natalensis,* Pretoria, SA)	M39	[4]
A. saalfeldensis	wild type (isolated from rock wall, Feengrotten, Saalfeld, GER)	-	[5]
Amycolatopsis sp. PS_44_ISF1	wild type (isolated from uropygial gland secretions of *Pachycephala schlegelii*, Yawan, Huon Peninsula, PNG)	PS_44_ISF1	This study: Bio Project PRJNA873226
Pseudoxylaria sp. X802	Wild type (isolated from the fungus comb of *Macrotermes natalensis,* Pretoria, SA)	X802	[6]
E. coli K12 HB101::pRK2013	Helper plasmid for mobilisation of non-self-transmissible plasmids in triparental conjugation, Kan^R		Laboratory strain
E. coli BacOpt2.0::pAsa	BAC vector containing Asa BGC from *A. saalfeldensis*		Terra Bioworks
Streptomyces albus J1074	Host for heterologous production of natural products		[7]
S. albus::pAsa	Heterologous host harbouring the Asa BGC		This study
S. coelicolor M1146	Host for heterologous production of natural products		[8]
S. coelicolor::pAsa	Heterologous host harbouring the Asa BGC		This study
S. lividans TK24	Host for heterologous production of natural products		[9]
S. lividans::pAsa	Heterologous host harbouring the Asa BGC		This study

Apr^R – apramycin resistance, Kan^R – kanamycin resistance, Cam^R – chloramphenicol resistance

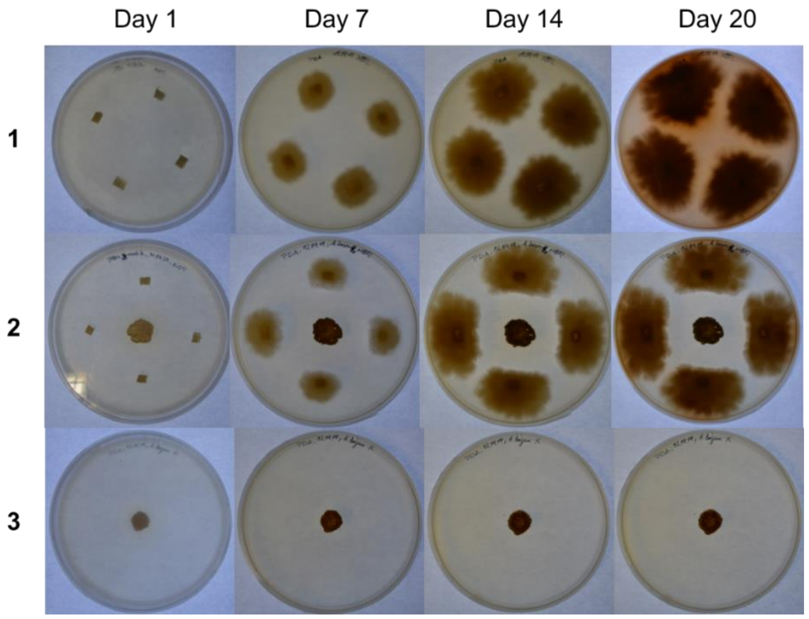

Figure S13. Representative co-cultivation of *Amycolatopsis* sp. PS_44_ISF1 with *Pseudoxylaria* sp. X802 over a cultivation period of 20 days. 1) *Pseudoxylaria* sp. X802 in axenic culture, 2) *Amycolatopsis* sp. PS_44_ISF1 and *Pseudoxylaria* sp. X802 in co-culture, 3) *Amycolatopsis* sp. PS_44_ISF1 in axenic culture.

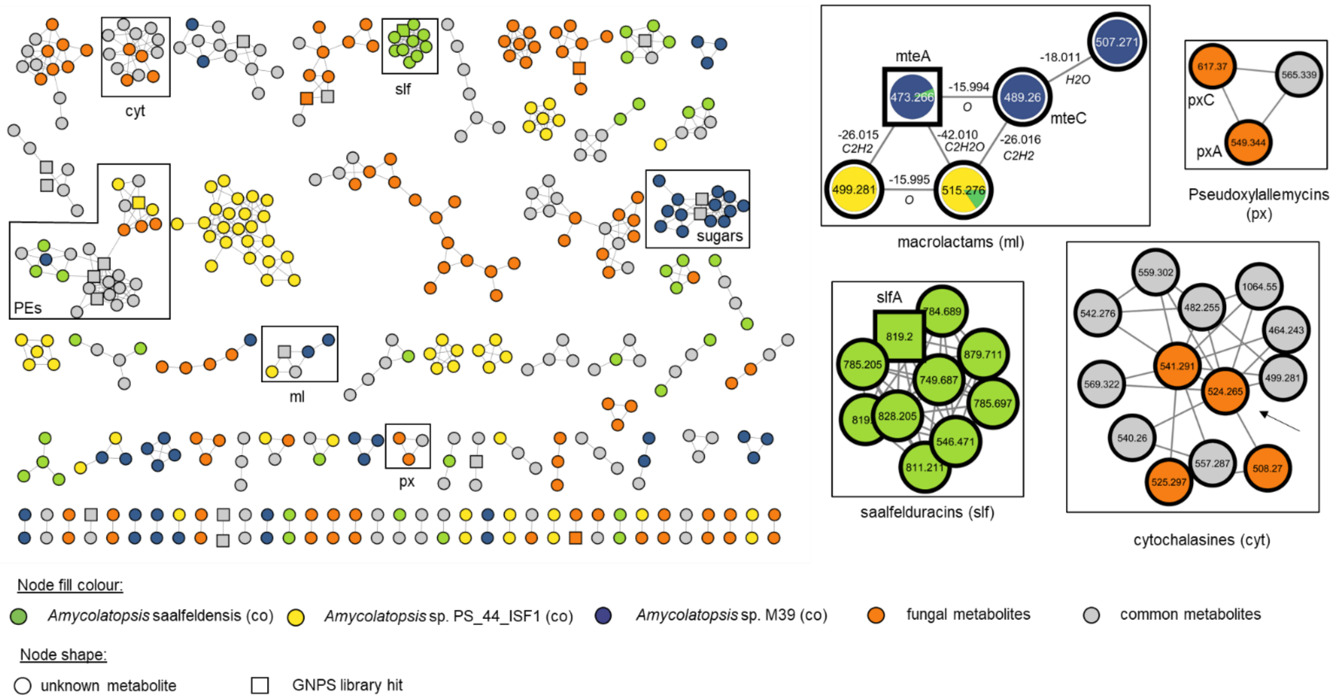

Figure S14. Comparative MS-based network analysis of methanolic extracts from *Amycolatopsis* spp. in co-cultivation with *Pseudoxylaria* sp. X802. GNPS cluster showing common and unique metabolites produced by *Amycolatopsis* spp. when co-cultured with X802. As a control, a X802 axenic culture was included to the analysis to identify fungal metabolites (●). Compounds unique for each *Amycolatopsis* strain are depicted by different node fill colours (●, ●, ●) and common metabolites are represented by grey nodes. Clusters were annotated using GNPS library hits (squared nodes) and known compounds from the literature (PEs: phosphoethanolamines) (cosine score: 0.75).

Supplementary Note 1

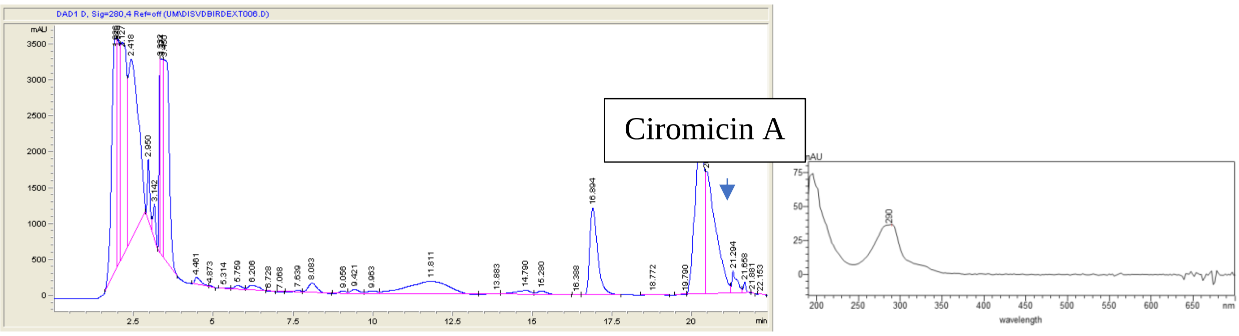

Figure S15. HPLC chromatogram and UV spectrum and HRMS spectrum of ciromicin A used for the isolation.

Deduction of chemical structure of Ciromicin A

Analysis of the ^{1}H NMR spectrum of ciromicin A (**1**) in DMSO-d_6 revealed the presence of fourteen olefin protons (δ_H 6.84, 6.83, 6.62, 6.51, 6.32, 6.28, 6.26, 6.09, 6.02, 6.00, 5.82, 5.45, and 5.41), six oxygenated methine protons (δ_H 4.87, 4.41, 4.15, 3.77, 3.67, and 3.28), one methine proton (δ_H 2.12), one amine-bearing methine proton (δ_H 2.50), two oxygenated methylene protons at (δ_H 3.67 and 2.99), and two *N*-amidated methylene protons (δ_H 3.80 and 2.89), along with two methyls (δ_H 1.36 and 1.03) displaying polyunsaturated features. The existence of two quaternary carbons, eighteen methines, and one methylenes, and two methyl groups was demonstrated by the HSQC and HMBC spectra requiring a cyclic structure to be incorporated in **1** with the degrees of unsaturation. The HSQC and HMBC spectra of ciromicin A indicated the presence of fourteen olefinic carbon signals (δ_C 140.3, 139.1, 135.8, 133.5, 133.5, 132.7, 130.7, 130.4, 129.5, 128.6 (2C), 127.9, 125.3, and 124.0), eight oxygen-bearing carbons (δ_C 81.1, 77.3, 76.6, 70.2, 66.7, and 58.7) including one quaternary carbon (δ_C 77.1) and an anomeric carbon at (δ_C 106.2), one *N*-amidated methylene carbon (δ_C 50.2), and two methyl groups (δ_C 26.7 and 15.3).

Comprehensive analysis of 2D NMR data (COSY, HSQC, and HMBC) confirmed the planar structure of ciromicin A (Figure S15). Interpretation of COSY spectrum revealed the connectivities among H-1'/H-2'/H-3'/H-4'/H_2-5' supported by the HMBC correlations indicating a presence of a deoxypentopyranose aminosugar group. The ^{1}H–^{1}H COSY correlations among the aliphatic methines H-2/H-3/H-4/H-5/H-6/H-7 established the connectivity from C-2 to C-7. In addition, further extension of the spin system from C-9 to C-21 including an epoxide (C-18 and C-19) and a methyl group (C-23) was assigned by COSY and *J*-couplings. Based on the HMBC data, the long-range heteronuclear correlations from the methyl protons H3-22 (δ_H 1.36) to C-7, C-8, and C-9 and from H-9 (δ_H 4.41) to anomeric carbon C-1' confirmed connectivities of the spin systems assigning the connectivity from C-2 to C-21 along with the deoxypentopyranose aminosugar group. The moiety accounts for ten of the eleven degrees of unsaturation inherent to the molecular formula, indicating that ciromicin A must consist of a cyclic structure. The connectivity of the ring was deduced from correlations between C-1 (δ_C 165.3) and NH-21, which completed the 22-membered macrolactam core structure of ciromicin A. On the basis of the characteristic large vicinal ^{1}H–^{1}H coupling constants (J = 15.0 Hz), the double bonds configurations were

assigned as 2*E*, 4*E*, 6*E*, 12*E*, 14*E*, while the alkene geometry at C-10 and C-16 was assigned as 10*Z* and 16*Z* showing coupling constant of 10.0 Hz. ROESY correlations established the stereochemistry of the five chiral centers of the ciromicin moiety. A strong NOE correlation was observed between H-9 and H-12 while a weak correlation between H-9 and H_3-22 was detected, comparable to that observed for originally reported ciromicin A.[10] Together, these deduction led to the configuration of C-8 as *R** and C-9 as *S**. Also, the relative configurations of the epoxide methine carbons at C-18 and C-19 were established by ROESY correlations among H-15/H-18/H-19. Similarly, the methine carbon at C-20 was determined as *S** by NOE correlation between H-17 and H-20. The relative stereochemistry of the deoxypentopyranose aminosugar moiety was confirmed by the large vicinal coupling constants of $J_{1',2'}$ = 7.5 Hz, $J_{2',3'}$ = 9.0 Hz, $J_{3',4'}$ = 9.0 Hz, and $J_{4',5'ax}$ = 10.0 Hz, displaying axial configuration of H-1', H-2', H-3', and H-4'. In addition, comparison of MS^2 spectra of ciromicin A and its putative precursor revealed a similar fragmentation pattern with xylosamine sugar unit (m/z 132.066) (Figure S15).

Table S5. NMR data of ciromicin A in DMSO-d_6.

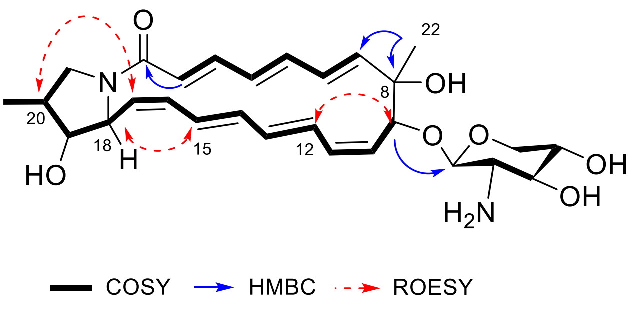

ciromicin A

N°	δ_C[a]	δ_H	mult (*J* in Hz)
1	165.3		
2	124.1	6.09	d (15.0)
3	139.1	6.84	dd (15.0, 9.5)
4	128.6	6.04	dd (15.0, 9.5)
5	135.8	6.26	dd (15.0, 8.5)
6	125.3	6.02	dd (15.0, 8.5)
7	140.3	5.82	d (15.0)
8	77.1		
9	81.1	4.41	d (9.5)
10	133.5	5.41	dd (10.0, 9.5)
11	127.9	6.00	dd (10.0, 10.0)
12	130.8	6.62	dd (15.0, 10.0)
13	132.7	6.32	dd (15.0, 10.0)
14	135.5	6.51	dd (15.0, 10.5)
15	128.6	6.83	dd (15.0, 10.5)
16	130.4	6.28	dd (10.0, 10.0)
17	129.5	5.45	dd (10.0, 10.0)
18	58.7	4.87	dd (10.0, 8.0)
19	77.5	3.77	m
20	36.2	2.12	m
21	50.2	3.56	dd (11.5, 8.0)
		2.89	dd (11.5, 10.0)
22	26.7	1.36	s
23	15.5	1.06	d (6.5)
1’	106.2	4.15	d (7.5)
2’	58.4	2.50	m[b]
3’	76.6	3.00	dd (9.0, 9.0)
4’	70.2	3.28	ddd (10.0, 9.0, 5.0)
5’eq	66.7	3.67	dd (11.0. 5.0)
5’ax		2.99	dd (11.0, 10.0)

[a] The assignments were based on COSY, gHSQC, TOCSY, and gHMBC experiments.
[b] overlapped

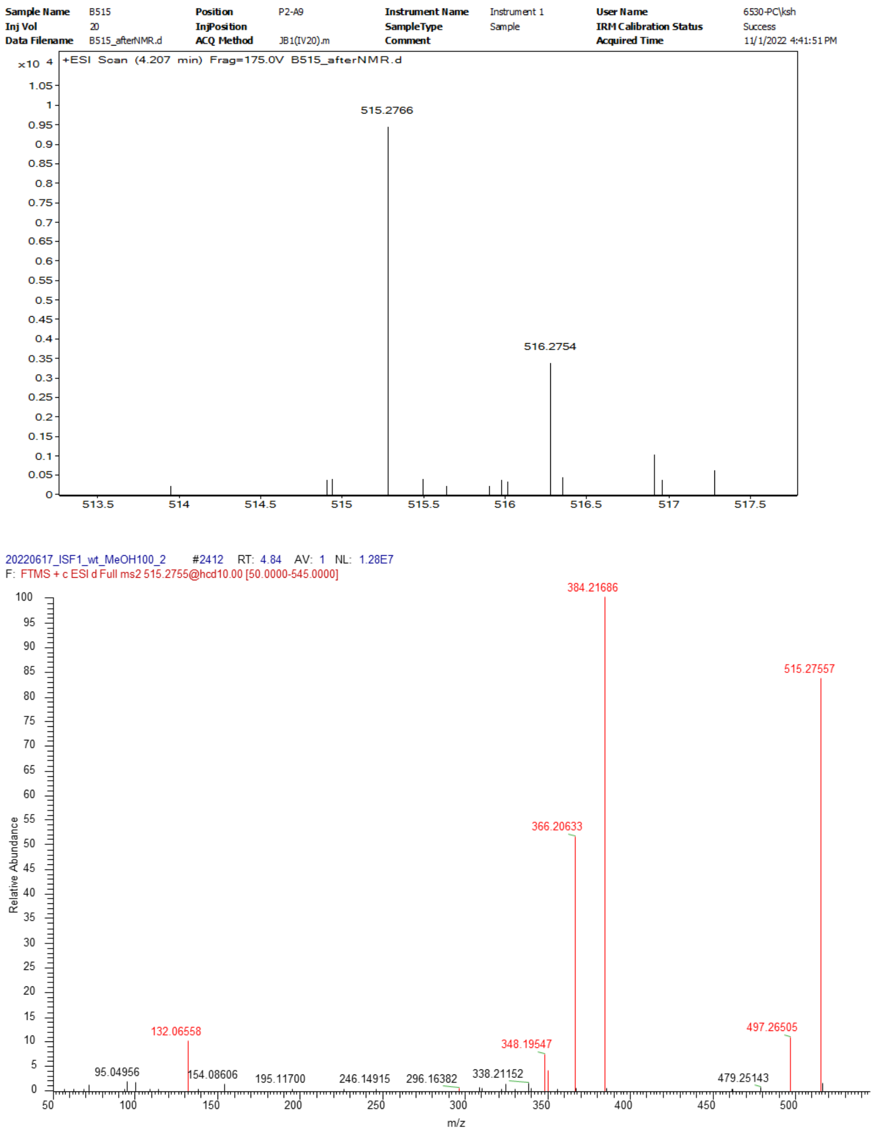

Figure S16. HRMS and MS/MS spectrum of ciromicin A. Fragments highlighted in red matched with computed MS/MS spectra from the CFM-ID 4.0.[11]

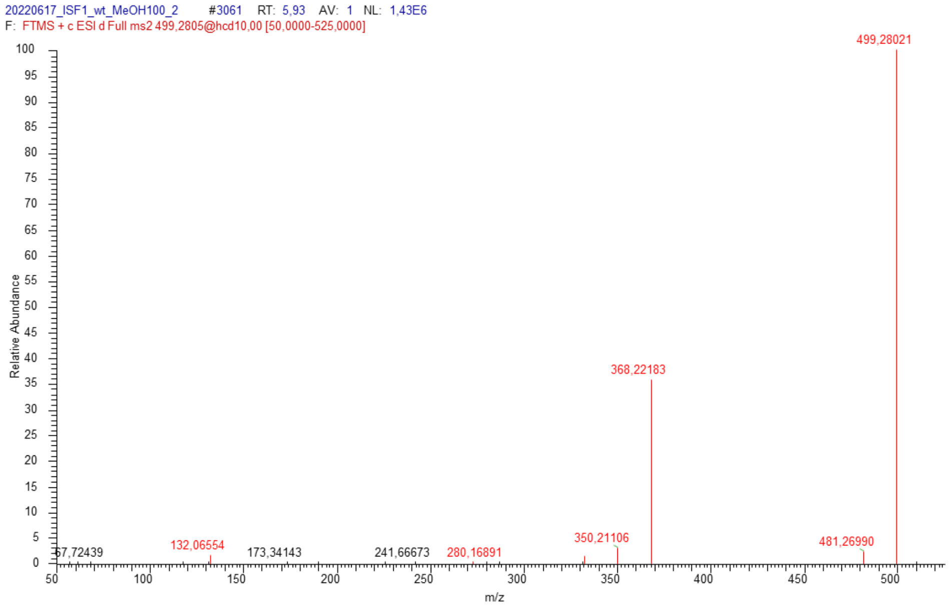

Figure S17. MS/MS spectrum of ciromicin precursor III. Fragments highlighted in red matched with computed MS/MS spectra from the CFM-ID 4.0.[11]

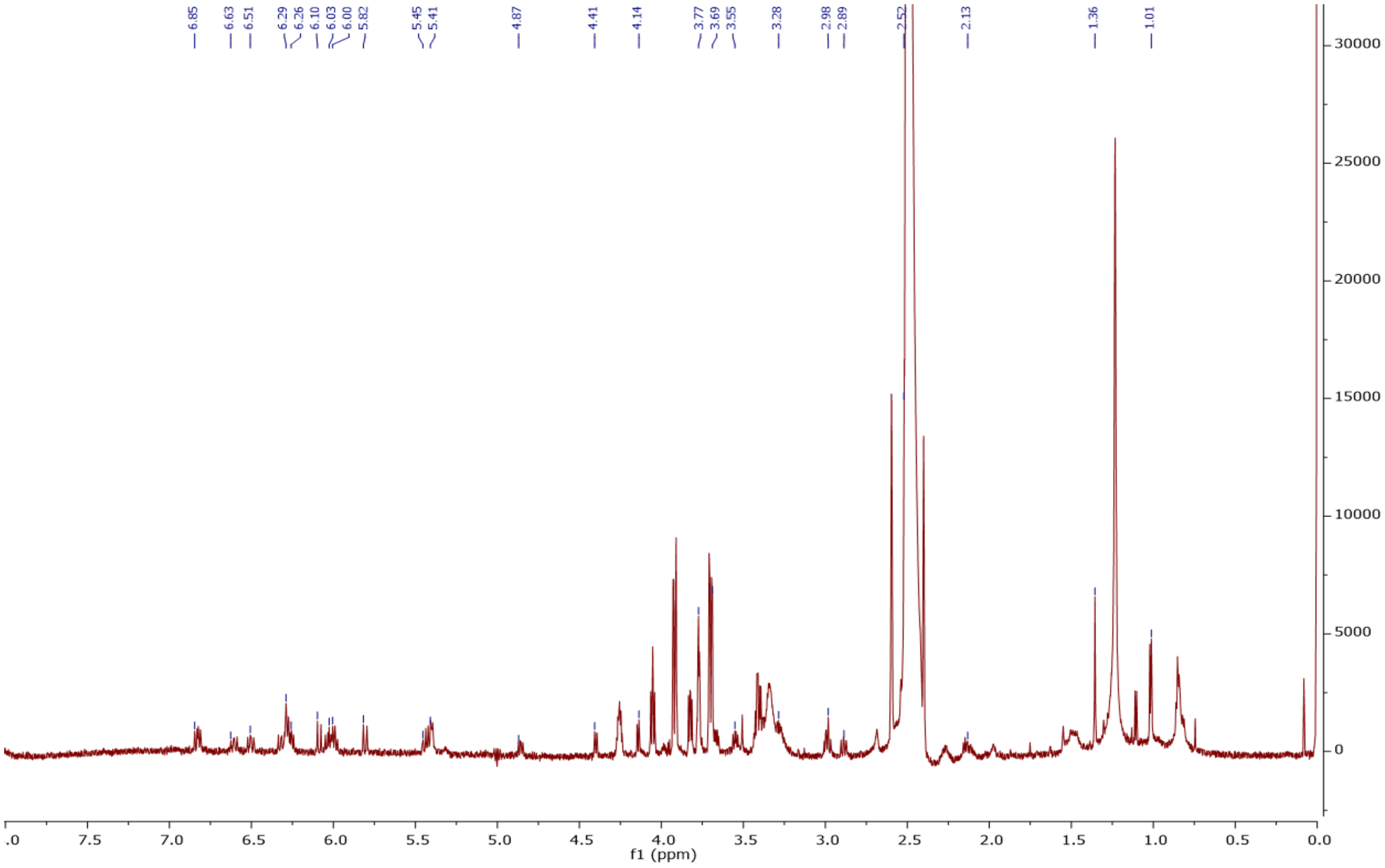

Figure S18. ^{1}H NMR spectrum of ciromicin A (**1**) in DMSO-d_6.

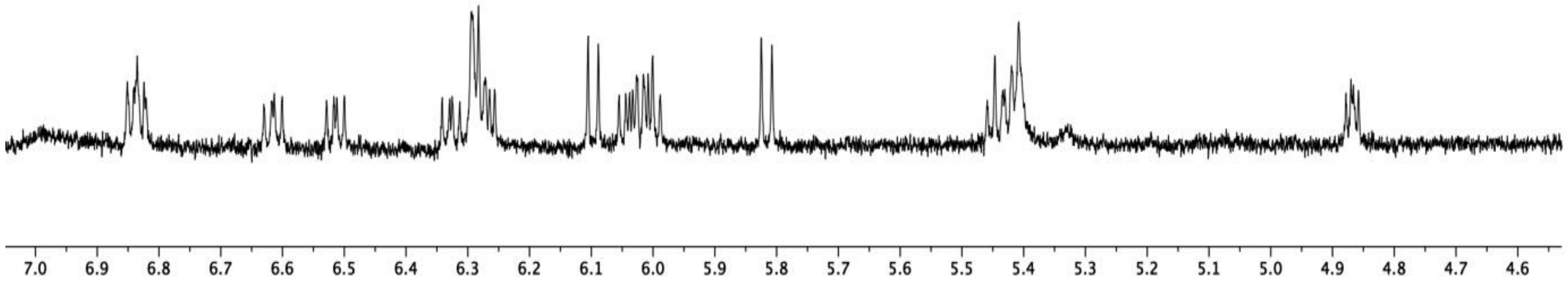

Figure S19. Magnified ^{1}H NMR spectrum (4.5 – 7.0 ppm) of ciromicin A (**1**) in DMSO-d_6.

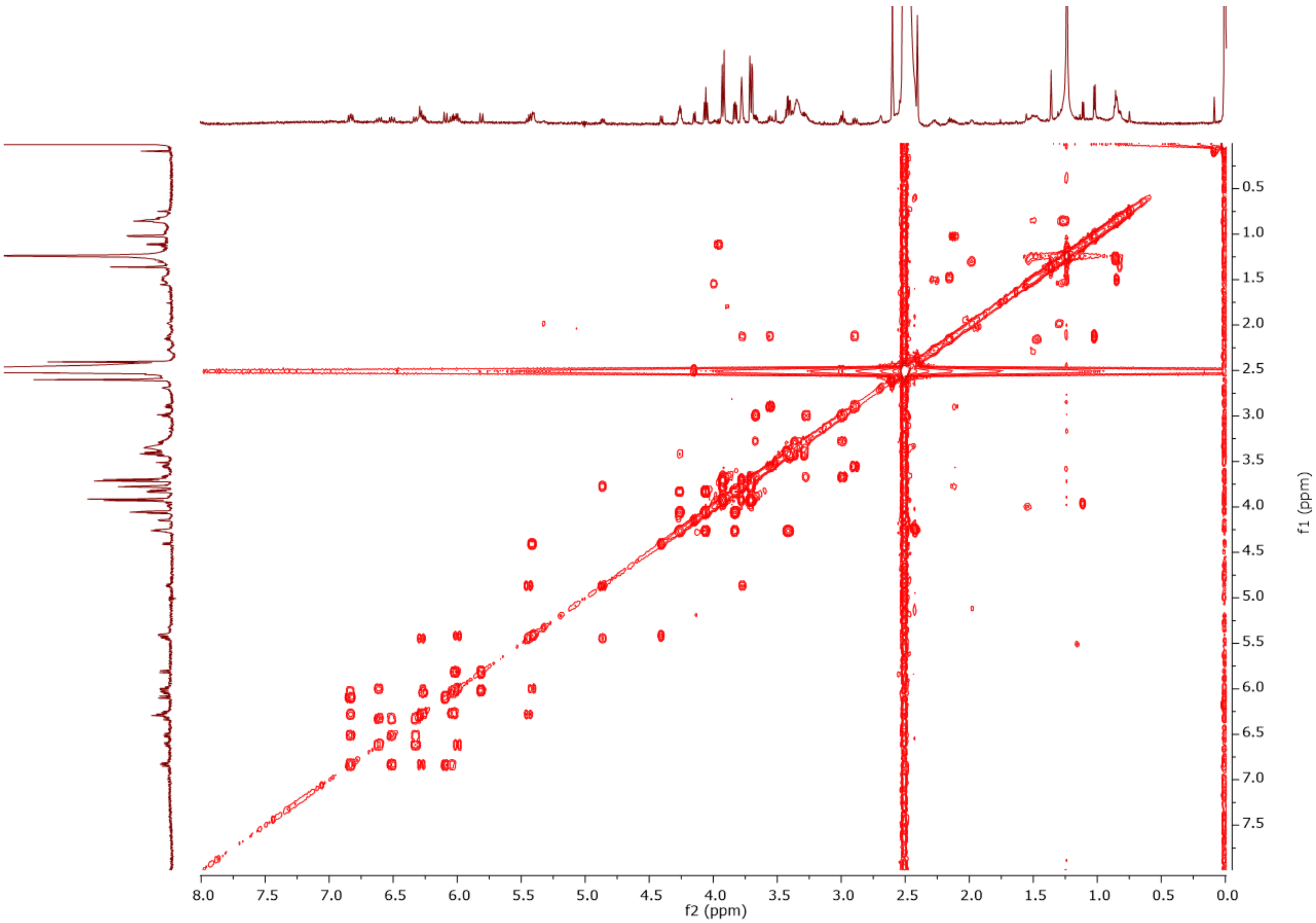

Figure S20. COSY NMR spectrum of ciromicin A (**1**) in DMSO-d_6.

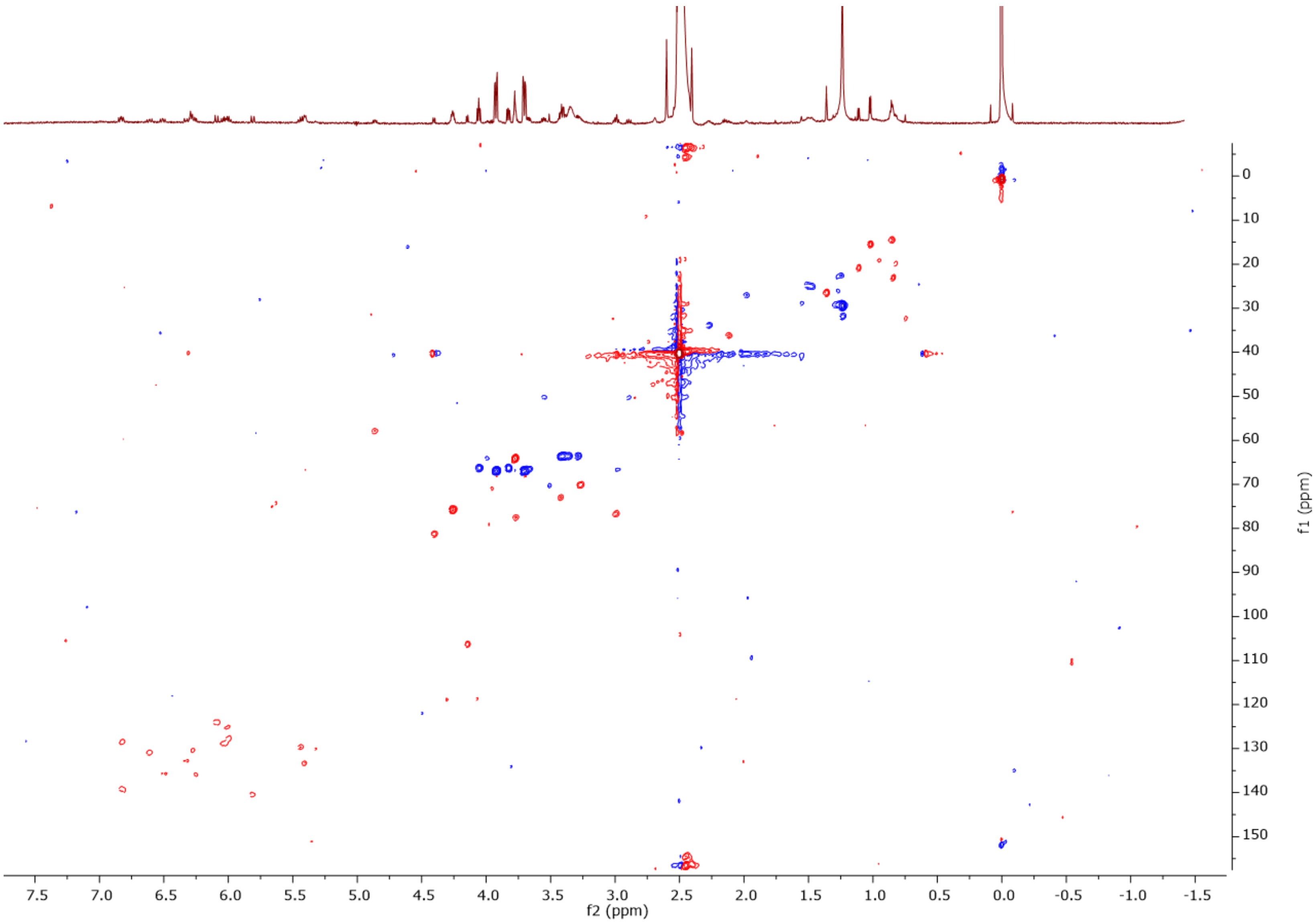

Figure S21. HSQC NMR spectrum of ciromicin A (**1**) in DMSO-d_6.

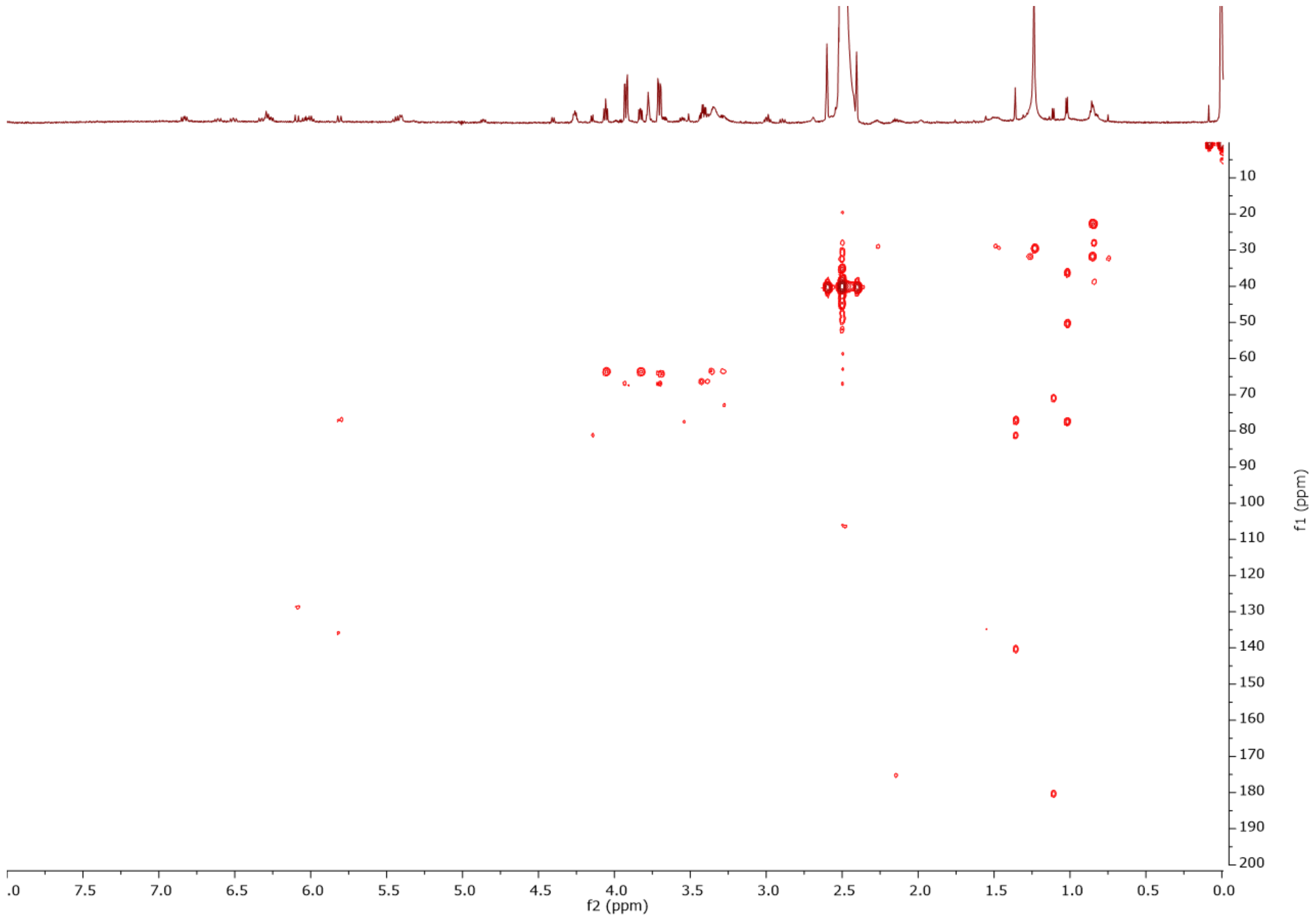

Figure S22. HMBC NMR spectrum of ciromicin A (**1**) in DMSO-d_6.

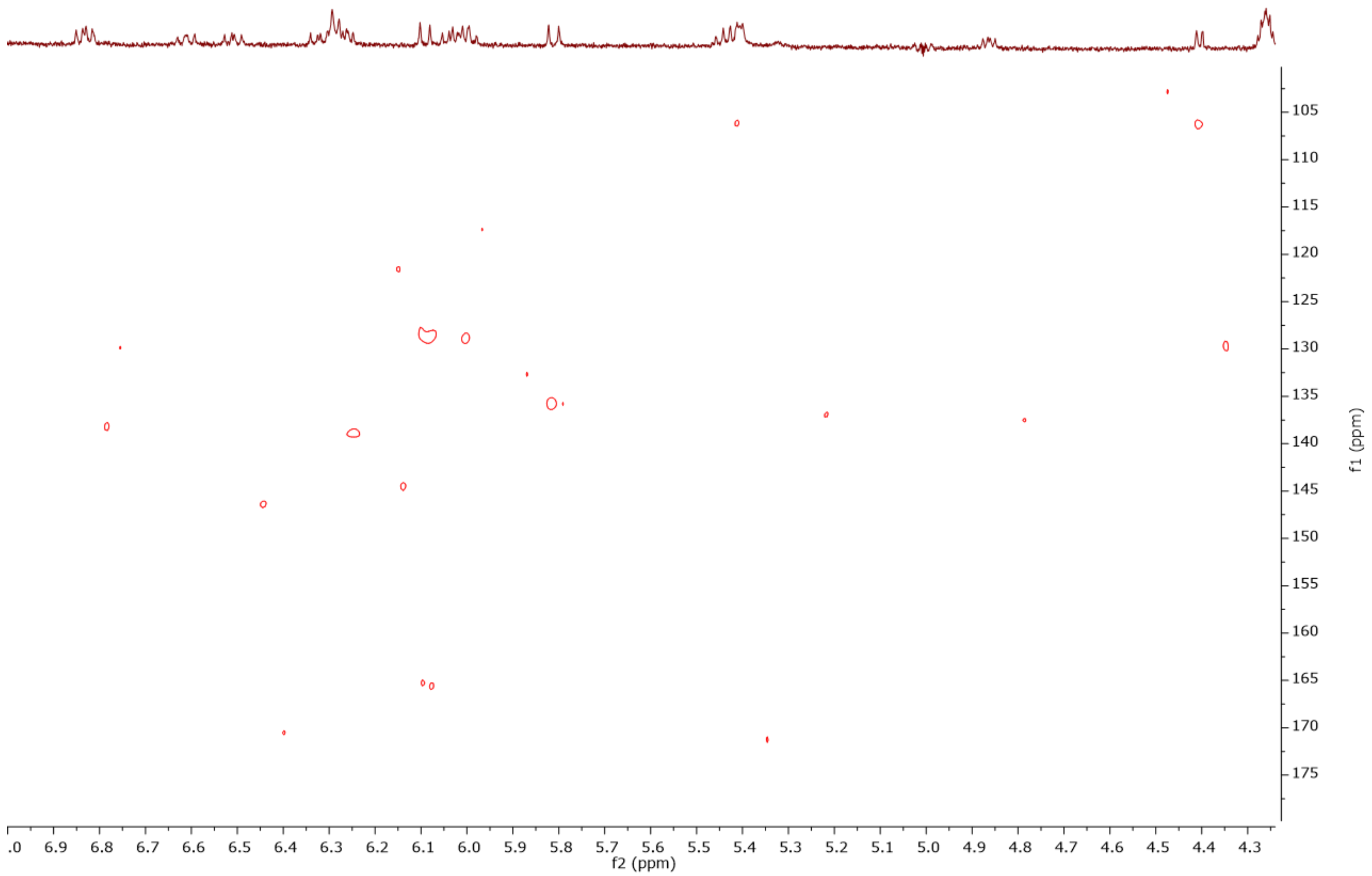

Figure S23. Magnified HMBC NMR spectrum of ciromicin A (**1**) in DMSO-d_6 showing the HMBC correlations from H-9 to C-1' and from H-2 to C-1.

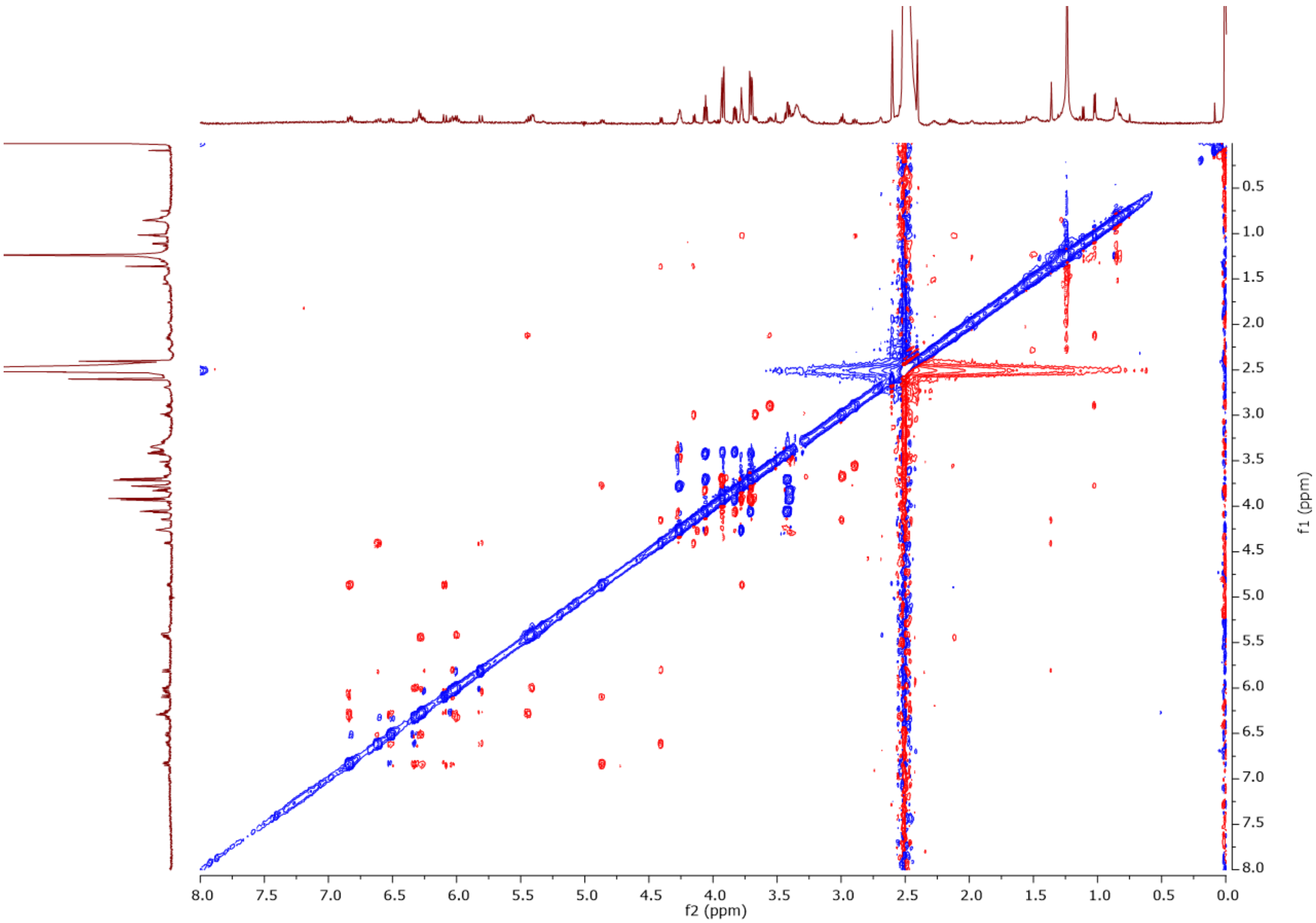

Figure S24. ROESY NMR spectrum of ciromicin A (**1**) in DMSO-d_6.

Supplementary Note 2

Structural characterization of macrotermycins:

Macrotermycin E (**2**) and macrotermycin F (**3**) were obtained as yellowish amorphous powders. Their molecular formulas, established as $C_{26}H_{36}N_2O_6$ by HRESIMS on the basis of molecular ion peaks $[M+H]^+$ at *m/z* 473.2652.

20-Membered macrocyclic ring structure: Analysis of NMR spectroscopic data revealed that **2** and **3** shared the same 20-membered macrocyclic core and aminosugar moiety with macrotermycin A. As previously reported for macrotermycin A, their ^1H NMR spectra (in CD_3OD) displayed signals of a polyene core structure with 14 olefinic protons ranging from δ_H 7.20 to δ_H 5.20, along with one secondary methyl group at δ_H 1.02 for **2** and δ_H 1.05 for **3**, and one tertiary methyl group at δ_H 1.33 for **2** and δ_H 1.45 for **3**. In addition, ten protons between δ_H 4.30 and δ_H 2.50 indicated carbinol and aliphatic protons (Table S7). The amide functionality was supported by an IR absorption at 1660 cm^{-1} for **2** and 1652 cm^{-1} for **3** (C=O stretch). Analysis of the gHSQC spectrum assigned 14 olefinic carbons, four oxygenated methines, one oxygenated methylene, one aliphatic methine, one aminated methine, one *N*-amidated methylene, and two methyl groups. The spin systems from C-2 to C-5 and from C-7 to C-19 were assigned by key gCOSY and TOCSY correlations (Figure S25). Moreover, key gHMBC correlations H_3-20/C-5, H_3-20/C-6, and H_3-20/C-7 assigned the single methyl group at the quaternary carbon C-6 and the connectivity of the C-2 to C-5. The core 20-membered macrocyclic ring structure of macrotermycin A-type was confirmed by gHMBC correlations along the carbon chain from C-7 to C-19 and by the gHMBC correlations from H-2, H-3 and H-19 to the carbonyl carbon C-1.

Aminosugar moiety: The deoxypentopyranose aminosugar moiety (C-1' to C-5') exhibited a characteristic spin system deduced from gCOSY, TOCSY and gHMBC correlations. The connectivity of the aminosugar unit to C-7 was confirmed by the H-1′/C-7 gHMBC correlation. The relative stereochemistry of the sugar moiety was deduced by the large coupling constants of $J_{1',2'}$ = 7.0 Hz, $J_{2',3'}$ = 8.0 Hz, $J_{3',4'}$ = 9.0 Hz, and $J_{4',5'ax}$ = 10.0 Hz, indicating axial configuration of H-1', H-2', H-3', and H-4'. These configurations were confirmed by comparing the NMR chemical shifts of the sugar moiety of **2** and **3** with those of macrotermycin A.

The **double bond configurations** of **2** and **3** were assigned as 2*E*, 4*E*, 10*E*, and 14*E* based on the large coupling constant between each proton (15.0 Hz). The 8*Z*, 12*Z*, and 16*Z* geometries were determined by their *cis*-coupling constants (11.0 Hz, 10.0 Hz or 9.5 Hz). Moreover, the geometries of double bonds were confirmed by H-2/H-4, H-3/H-5, and H-14/H-16 ROESY correlations.

Stereochemistry: Comparative NMR analysis indicated that **2** is a C-7 stereoisomer of macrotermycin A, as it exhibits a major ^{13}C chemical shift difference at C-7 (δ_C 79.9 for macrotermycin A and δ_C 86.5 for **2**) (Table S8). Moreover, the vicinal coupling constant (6.7 Hz) between H-7 and H-8 and a significant correlation between H-7 and H-8 in the ROESY spectrum of **2** supported the relative configuration at C-7 and C-8 as a *syn*-configuration (Figure S25). Finally, ROESY correlation of H-8/H-20 and H-7/H-20 suggested that the relative configurations of C-6 and C-7 were 6*S** and 7*R**, respectively.

For compound **3**, key ROESY correlations of H-7/H-8, H-7/H-20 and H-8/H-20 suggested that the relative configurations of C-6 and C-7 in **3** are identical to those of **2**. As for macrotermycin A, H-15/H-18 key ROESY correlation allowed us to establish the stereochemistry of C-18 in macrotermycin E as *S**. The stereochemistry of the C-18 chiral center of **3** was determined as *R* by comparing its chemical shifts of ^{13}C NMR to those of **2** in a same solvent (CD_3OD), which indicated that the ^{13}C chemical shift pattern of macrotermycin F was not in agreement

with those of macrotermycin E at positions C-17, C-18, and C-19. Moreover, this observation was confirmed by the absence of ROESY correlation between H-15 and H-18.

In conclusion, and as previously reported,[12] the ROESY correlation between H-7 and H-10 and the missing correlation between H-7/H-20 and H-7/H-8 ones, in addition of the vicinal coupling constant (9.0 Hz) between H-7 and H-8 led us deduce a 7*S** configuration of macrotermycins A and C (Figure S25).

According to this interpretation and all the comparative NMR data tables between macrotermycin A, compound **2**, and **3**, we propose to revise the C-7 configuration of macrotermycin A.

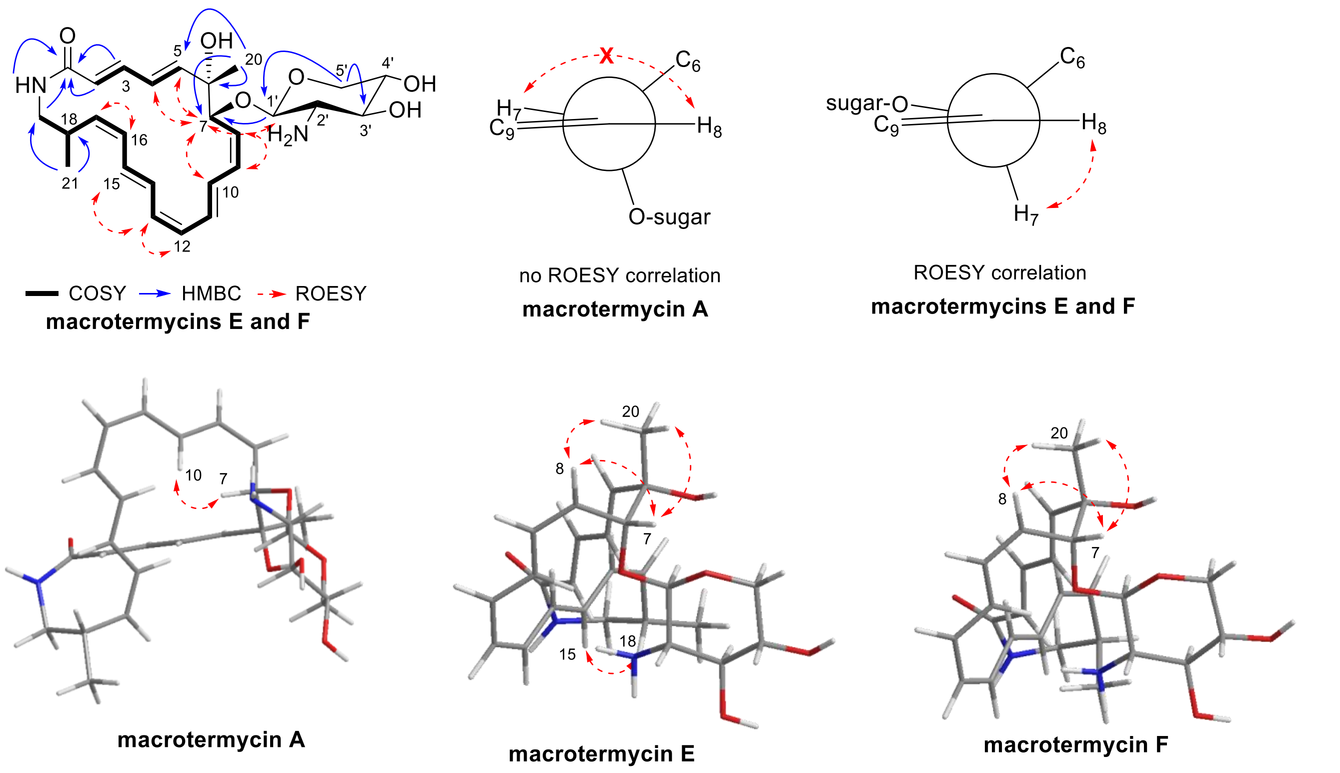

Figure S25. Proposed Newman projection at the C-7 to C-8 bond of macrotermycins A,E, and F and key 2D NMR correlations of macrotermycins A, E, and F.

Table S6. ^{1}H (600 MHz) and ^{13}C NMR (150 HMz) data of **macrotermycin E** in DMSO-d_6.

N°	Macrotermycin E	
	δ_C (DMSO-d_6)[a]	δ_H (J in Hz)
1	165.7 s	
2	124.5 d	6.00, d (15.0)
3	139.2 d	7.02, dd (15.0, 10.5)
4	128.1 d	6.44, dd (15.0, 10.5)
5	145.7 d	6.01, d (15.0)
6	75.5 s	
7	86.5 d	4.10, d (6.7)
8	129.3 d	5.56, dd (10.0, 6.7)
9	129.7 d	5.97, dd (10.0, 10.0)
10	131.0 d	6.06, dd (15.0, 10.0)
11	131.6 d	6.56, dd (15.0, 10.0)
12	131.0 d	6.05, dd (10.0, 10.0)
13	131.0 d	6.05, dd (10.0, 10.0)
14	131.0 d	6.04, dd (15.0, 10.0)
15	128.9 d	6.23, dd (15.0, 10.0)
16	129.3 d	5.94, dd (10.0, 10.0)
17	136.7 d	5.20, dd (10.0, 9.5)
18	31.3 d	3.01, m
19	46.5 t	3.22, m
		2.61, m
20	26.4 q	1.26, s
21	19.2 q	0.94, d (6.5)
19-NH		
1′	106.9 d	4.20, d (7.5)
2′	58.0 d	2.29, dd (8.5, 7.5)
3′	76.4 d	3.01, dd (9.0, 8.5)
4′	69.6 d	3.23, ddd (10.0, 9.0, 5.0)
5′eq	66.1 t	3.60, dd (10.5, 5.0)
5′ax		3.00, dd (10.5, 10.0)

[a] The assignments were based on COSY, gHSQC, TOCSY, and gHMBC experiments.

Table S7. Comparison of ^{1}H (600 MHz) and ^{13}C NMR chemical shifts of macrotermycin A and macrotermycin E in DMSO-d_6.

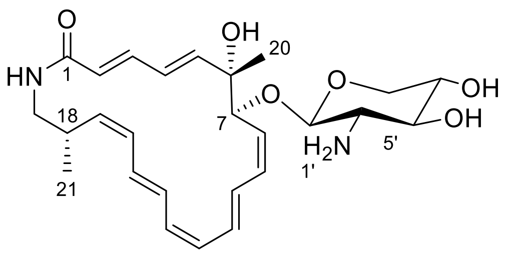

revised structure of
macrotermycin A

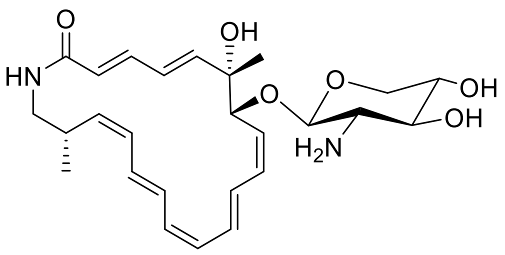

macrotermycin E

N°	Macrotermycin A		Macrotermycin E		$\lvert\Delta\delta_{(\text{macrotermycin A - macrotermycin E})}\rvert$	
	δ_C (ppm)	δ_H (ppm)	δ_C (ppm)	δ_H (ppm)	δ_C (ppm)	δ_H (ppm)
1	165.3		165.7		0.4	
2	123.7	5.57	124.5	6.00	0.8	0.43
3	137.8	6.55	139.2	7.02	1.4	0.47
4	126	5.89	128.1	6.44	2.1	0.55
5	145.1	5.76	145.7	6.01	0.6	0.25
6	75.3		75.5		0.2	
7	79.9	4.38	86.5	4.10	**6.6**	0.28
8	131.1	5.39	129.3	5.56	1.8	0.17
9	127.1	5.93	129.7	5.97	2.6	0.04
10	129	6.28	131	6.06	2.0	0.22
11	128.2	6.03	131.6	6.56	3.4	0.53
12	129.2	6.01	131.0	6.05	1.8	0.04
13	129.2	6.31	131.0	6.05	1.8	0.26
14	129.5	6.34	131.0	6.04	1.5	0.30
15	126.7	6.38	128.9	6.23	2.2	0.15
16	128.9	6.05	129.3	5.94	0.4	0.11
17	135.2	5.10	136.7	5.20	1.5	0.10
18	31.4	3.02	31.3	3.01	0.1	0.01
19	44.6	3.10	46.5	3.22	1.9	0.12
		2.72		2.61		0.11
20	26.2	1.33	26.4	1.26	0.2	0.07
21	17.5	0.91	19.2	0.94	1.7	0.03
1′	104.8	4.20	106.9	4.20	2.1	0.00
2′	57.3	2.50	58.0	2.29	0.7	0.21
3′	75.6	3.03	76.4	3.01	0.8	0.02
4′	69.1	3.27	69.6	3.23	0.5	0.04
5′eq	65.5	3.68	66.1	3.60	0.6	0.08
5′ax		3.01		3.00		0.01
				**Average**	**1.53**	**0.18**
				s	**1.31**	**0.16**
				**R^2**	**0.999361**	**0.991275**

Table S8. ^{1}H (600 MHz) and ^{13}C NMR (150 HMz) data of macrotermycin E and macrotermycin F in CD_3OD.

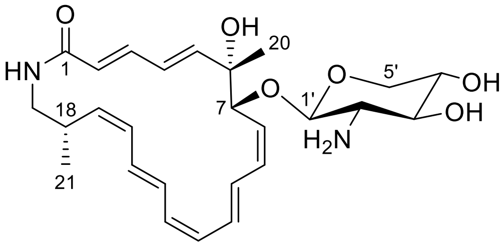

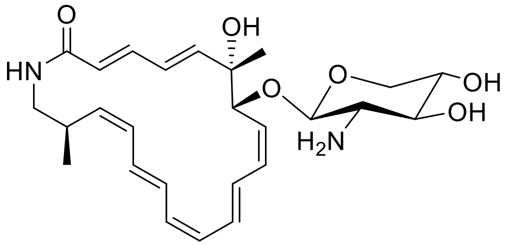

N°	Macrotermycin E		Macrotermycin F	
	δ_C (CD_3OD)[a]	δ_H (*J* in Hz)	δ_C (CD_3OD)[a]	δ_H (*J* in Hz)
1	169.2 s		168.3 s	
2	124.4 d	6.02, d (15.0)	125.0 d	6.06, d (15.5)
3	141.9 d	7.17, dd (15.0, 11.0)	141.3 d	7.11, dd (15.5, 10.5)
4	129.0 d	6.51, dd (15.0, 11.0)	128.7 d	6.38, dd (15.5, 10.5)
5	146.9 d	6.12, d (15.0)	146.5 d	6.09, d (15.5)
6	77.1 s		76.7 s	
7	87.0 d	4.25, d (6.5)	84.7 d	4.24, d (8.6)
8	128.8 d	5.60, dd (11.0, 6.5)	129.8 d	5.58, dd (11.5, 8.6)
9	131.9 d	6.13, dd (11.0, 11.0)	130.6 d	6.06, dd (11.5, 11.0)
10	132.6 d	6.10, dd (15.0, 11.0)	133.7 d	6.10, dd (15.0, 11.0)
11	132.4 d	6.70, dd (15.0, 11.0)	129.7 d	6.28, dd (15.0, 11.0)
12	131.9 d	6.12, dd (11.0, 11.0)	128.6 d	5.85, dd (11.0, 11.0)
13	131.9 d	6.13, dd (11.0, 11.0)	131.1 d	5.93, dd (11.0, 11.0)
14	131.9 d	6.13, dd (15.0, 11.0)	130.6 d	6.07, dd (15.0, 11.0)
15	129.8 d	6.32, dd (15.0, 11.0)	130.7 d	6.06, dd (15.0, 11.0)
16	130.7 d	6.06, dd (11.0, 10.5)	126.3 d	6.25, dd (11.0, 11.0)
17	136.8 d	5.21, dd (10.5, 9.5)	139.1 d	5.67, dd (11.0, 6.0)
18	32.6 d	3.15, m	36.1 d	2.60, m
19	47.8 t	3.44, m<br>2.71, dd (14.0, 10.0)	44.9 t	3.38, m<br>3.17, dd (14.0, 9.5)
20	25.7 q	1.33, s	24.4 q	1.45, s
21	19.1 q	1.02, d (7.0)	18.8 q	1.05, d (7.0)
1′	106.9 d	4.36, d (8.0)	106.1 d	4.26, d (8.0)
2′	58.3 d	2.56, dd (9.0, 8.0)	58.3 d	2.61, dd (9.5, 8.0)
3′	77.3 d	3.20, dd (9.0, 9.0)	77.2 d	3.19, dd (9.5, 9.0)
4′	71.1 d	3.42, ddd (10.0, 9.0, 5.0)	71.1 d	3.44, ddd (10.5, 9.0, 5.0)
5′eq<br>5′ax	66.8 t	3.81, dd (12.0, 5.0)<br>3.13, dd (12.0, 10.0)	66.8 t	3.80, dd (11.5, 5.0)<br>3.11, dd (11.5, 10.5)

[a] The assignments were based on COSY, gHSQC, TOCSY, and gHMBC experiments.

Table S9. Comparison of ^{1}H (600 MHz) and ^{13}C NMR chemical shifts values of macrotermycin E and macrotermycin F in CD_3OD and shift differences.

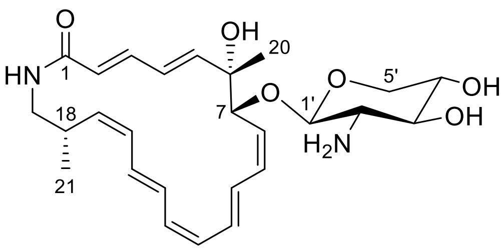

macrotermycin E

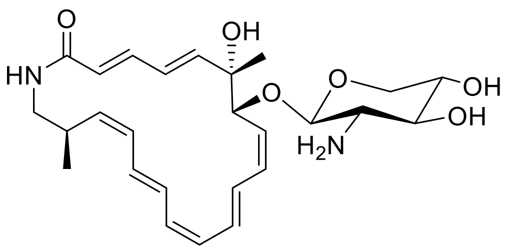

macrotermycin F

N°	Macrotermycin E		Macrotermycin F		$\|\Delta\delta_{(macrotermycin\ E\ -\ macrotermycin\ F)}\|$	
	δ_C (ppm)	δ_H (ppm)	δ_C (ppm)	δ_H (ppm)	δ_C (ppm)	δ_H (ppm)
1	169.2		168.3		0.9	
2	124.4	6.02	125.0	6.06	0.6	0.04
3	141.9	7.17	141.3	7.11	0.6	0.06
4	129	6.51	128.7	6.38	0.3	0.13
5	146.9	6.12	146.5	6.09	0.4	0.03
6	77.1		76.7		0.4	
7	87.0	4.25	84.7	4.24	2.3	0.01
8	128.8	5.60	129.8	5.58	1.0	0.02
9	131.9	6.13	130.6	6.06	1.3	0.07
10	132.6	6.10	133.7	6.10	1.1	0.00
11	132.4	6.70	129.7	6.28	2.7	0.42
12	131.9	6.12	128.6	5.85	3.3	0.27
13	131.9	6.13	131.1	5.93	0.8	0.20
14	131.9	6.13	130.6	6.07	1.3	0.06
15	129.8	6.32	130.7	6.06	0.9	0.26
16	130.7	6.06	126.3	6.25	4.4	0.19
17	136.8	5.21	139.1	5.67	2.3	0.46
18	32.6	3.15	36.1	2.60	**3.5**	**0.55**
19	47.8	3.44	44.9	3.38	2.9	0.06
		2.71		3.17		0.46
20	25.7	1.33	24.4	1.45	1.3	0.12
21	19.1	1.02	18.8	1.05	0.3	0.03
1′	106.9	4.36	106.1	4.26	0.8	0.10
2′	58.3	2.56	58.3	2.61	0.0	0.05
3′	77.3	3.20	77.2	3.19	0.1	0.01
4′	71.1	3.42	71.1	3.44	0.0	0.02
5′eq	66.8	3.81	66.8	3.80	0.0	0.01
5′ax		3.13		3.11		0.02
				**Average**	**1.29**	**0.14**
				s	**1.19**	**0.16**
				**R^2**	**0.999194**	**0.992731**

Supplementary Note 3

Macrotermycin G (4) was isolated as an amorphous powder.

The molecular formula of **4** was established as $C_{26}H_{36}N_2O_6$ by HRESIMS on the basis of molecular ion peak $[M+H]^+$ at *m/z* 473.2650.

The ^{1}H NMR spectrum displayed ten olefin signals between δ_H 6.43 and δ_H 5.04, a secondary methyl signal at δ_H 1.00, a tertiary methyl signal at δ_H 1.15, and 14 protons between δ_H 4.57 and δ_H 2.46 attributable to carbinol and aliphatic protons (Table S10). Analysis of gHSQC spectral data indicated ten olefinic methines, four oxygenated methines, one oxygenated methylene, six aliphatic methines, one aliphatic methylene, and two methyl carbons. The combination of gCOSY, TOCSY, and gHMBC NMR data assigned a [12+6+6] substructure and a pentopyranose aminosugar residue. Key gHMBC correlations of H-20/C-5, H-20/C-6 and H-20/C-7, as well as H-2/C-1, H-3/C-1, and H-19/C-1 secured the linkages of C-5→C-6→C-7 and the presence of a cyclic-lactam ring (C-1).

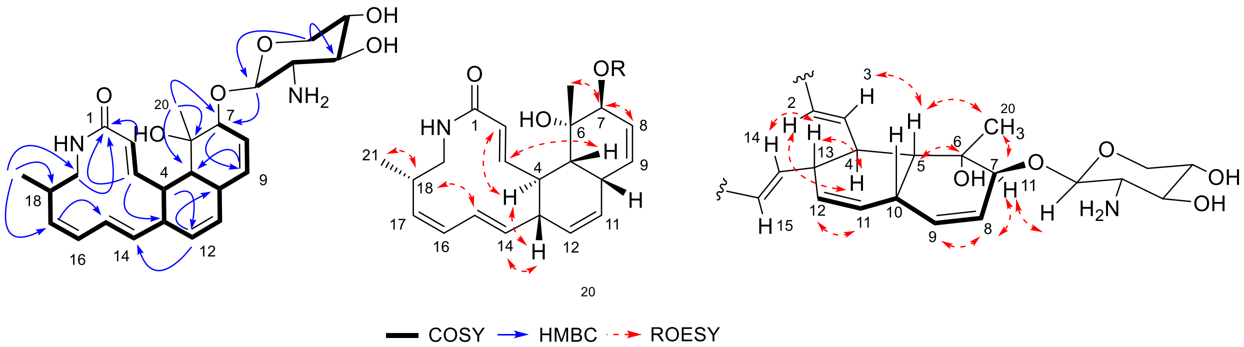

Figure S26. Key NMR correlations and proposed chemical structure of macrotermycin G (**4**).

The NMR data of **4** were very similar to those of macrotermycin B, with the most noticeable difference being the replacement of methylene carbon C-2 and oxymethine carbon C-3 by the double bond at C-2/C-3 (Table S11). The location of the aminosugar group was assigned to be C-7 by the gHMBC correlation between H-1′ (δ_H 4.57) and C-7 (δ_C 82.5), and the aminosugar was determined to be identical to that of macrotermycin B by comparing NMR data of the sugar, the coupling constants of the sugar protons, and ROESY spectrum.

The double bond geometries were established as 2*E*, 8*Z*, 11*Z*, 14*E*, and 16*Z* based on the coupling constants observed in the homo *J*-resolved ^{1}H NMR spectrum and corresponding ROESY correlations. The relative configuration of macrotermycin G (4*R**, 5*R**, 6*S**, 7*S**, 10*S**, 13*S**, 18*S**) was determined by analysis of coupling constants and ROESY experiment. H-4/H-13 ROESY correlation led to the supposition that H-4 and H-13 are *trans*-orientated but in an unusual close proximity ($J_{4,13}$ = 9.0 Hz) due to a low dihedral angle caused by the strained ring systems and the *cis*-decalin like system. The strong ROESY signal between H-13 and H-14 indicated a locked conformation of the 14*E* double bond. The relative large coupling constant between H-4/H-5 ($J_{4,5}$ = 9.5 Hz) and the missing ROESY signal indicated a *trans* relation. The supposed configuration was supported by intense H-4/H-2 and H-3/H-5 ROESY signals due to the 2*E* geometry. H-5/H-10 ROESY correlation indicated a *cis*-configuration, in agreement with strong ROESY signals from H_3-20 to H-5 and H-10, and H-10 to H-13 confirming the 6*S** and 13*S**

configurations. While H-7/H-20 and H-7/H-1’ ROESY correlations are observed, these cannot unambiguously resolve the stereoconfiguration of C-7 as both configurations would lead to these observations due to the close proximity. The absence of H-7/H-10 and H-7/H-5 ROESY correlations led to the hypothesis that H-7 is not in the same side of H-10 and H-5, which results in a proposed 7*S**-configuration. The stereochemistry at C-18 was determined by H-15/H-18 and H-18/H-21, supported by signals from H-21 to H-18, H-19a and to H-19b, and confirmed by considering of similar ^{13}C chemical shift pattern of C-17, C-18, and C-19 to that of macrotermycin B in a same solvent (Table S11).

Table S10. ^{1}H (600 MHz) and ^{13}C NMR (150 HMz) data of macrotermycin G in CD_3OD.

N°	**Macrotermycin G**	
	δ_C (CD_3OD)[a]	δ_H (*J* in Hz)
1	169.4 s	
2	125.7 d	5.67, d (15.5)
3	139.2 d	7.02, dd (15.0, 10.5)
4	148.1 d	6.43, dd (15.5, 10.0)
5	42.9 d	3.82, ddd (10.0, 9.5, 9.0)
6	47.1 d	2.61, dd (9.5, 9.0)
7	72.1 s	
8	82.5 d	3.90, d (5.5)
9	126.9 d	5.92, dd (10.0, 5.5)
10	132.0 d	5.95, dd (10.0, 3.0)
11	41.7 d	2.67, m
12	133.1 d	6.18, dd (8.5, 2.5)
13	132.5 d	5.65, dd (8.5, 1.0)
14	41.3 d	2.98, t (8.5)
15	131.0 d	6.03, dd (15.0, 9.5)
16	129.2 d	6.12, dd (15.0, 9.5)
17	131.1 d	6.15, dd (10.0, 9.5)
18	135.8 d	5.04, dd (10.0, 9.5)
19	33.6 d<br>45.7 t	2.82, m<br>3.53, dd (13.0, 5.0)
20	26.4 q	2.46, dd (13.0, 11.0)
21	26.8 q	1.15, s
19-NH	17.7 q	1.00, d (6.5)
1′	105.2 d	4.57, d (8.0)
2′	59.4 d	2.76, dd (9.5, 8.0)
3′	76.0 d	3.41, dd (9.5, 9.0)
4′	71.5 d	3.50, ddd (10.0, 9.0, 5.5)
5′eq	66.9 t	3.90, dd (11.5, 5.5)
5′ax		3.24, dd (11.5, 10.0)

[a] The assignments were based on COSY, gHSQC, TOCSY, and gHMBC experiments.

Table S11. Comparison of ^{1}H (600 MHz) and ^{13}C NMR chemical shifts of macrotermycin B and macrotermycin G in CD_3OD.

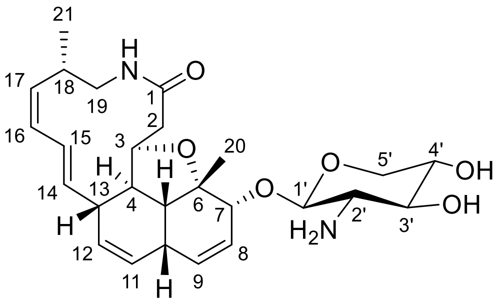

macrotermycin B

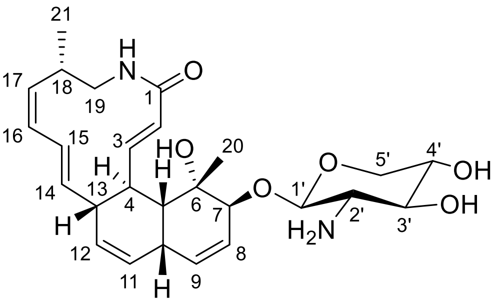

macrotermycin G

N°	Macrotermycin B		Macrotermycin G		|Δ*δ*(macrotermycin B – macrotemycin G)|	
	*δ*C (ppm)	*δ*H (ppm)	*δ*C (ppm)	*δ*H (ppm)	*δ*C (ppm)	*δ*H (ppm)
1	173.2		169.4		3.8	
2	41.5	2.66; 2.43	125.7	5.67	**84.2**	3.01; 3.24
3	80.2	4.25	148.1	6.43	**67.9**	2.18
4	49.6	1.71	42.9	3.82	6.7	2.11
5	49.6	1.99	47.1	2.61	2.5	0.62
6	n.d.		72.1			
7	80.4	3.75	82.5	3.90	2.1	0.15
8	126.8	5.78	126.9	5.92	0.1	0.14
9	129.4	5.62	132.0	5.95	2.6	0.33
10	35.5	2.92	41.7	2.67	6.2	0.25
11	129.8	5.87	133.1	6.18	3.3	0.31
12	131.4	5.53	132.5	5.65	1.1	0.12
13	44.7	2.73	41.3	2.98	3.4	0.25
14	134.8	5.18	131.0	6.03	3.8	0.85
15	129.8	6.23	129.2	6.12	0.6	0.11
16	131.1	6.12	131.1	6.15	0.0	0.03
17	135.1	5.13	135.8	5.04	0.7	0.09
18	31.4	3.14	33.6	2.82	2.2	0.32
19	46.4	3.53	45.7	3.53	0.7	0.00
		2.23		2.46		0.23
20	26.7	1.35	26.8	1.15	0.1	0.2
21	17.6	0.97	17.7	1.00	0.1	0.03
1′	107.7	4.32	105.2	4.57	2.5	0.25
2′	58.1	2.63	59.4	2.76	1.3	0.13
3′	77.0	3.24	76.0	3.41	1.0	0.17
4′	71.0	3.44	71.5	3.50	0.5	0.06
5′eq	66.5	3.87	66.9	3.90	0.4	0.03
5′ax		3.20		3.24		0.04

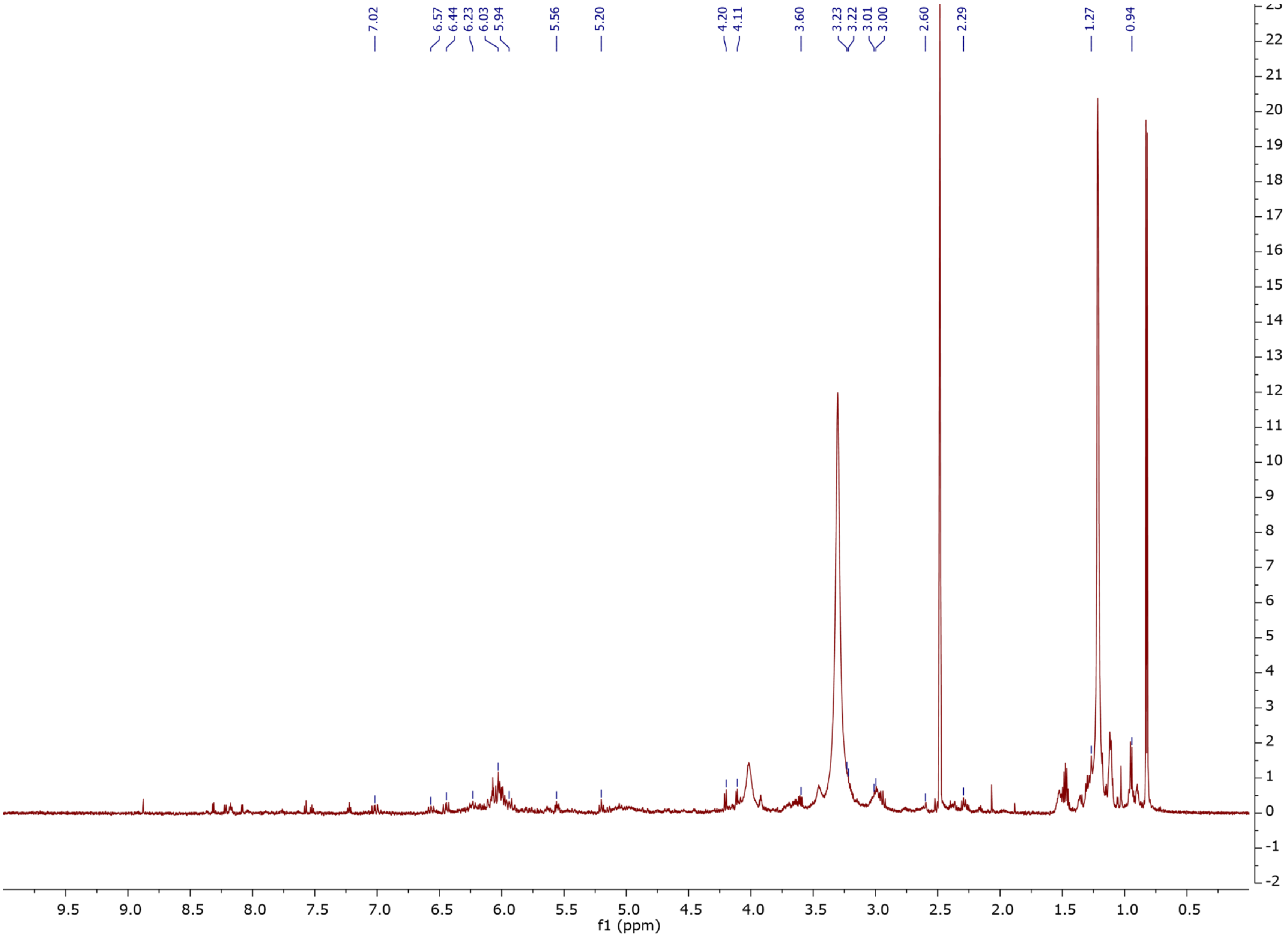

Figure S27. ^{1}H NMR spectrum of macrotermycin E (**2**) in DMSO-d_6.

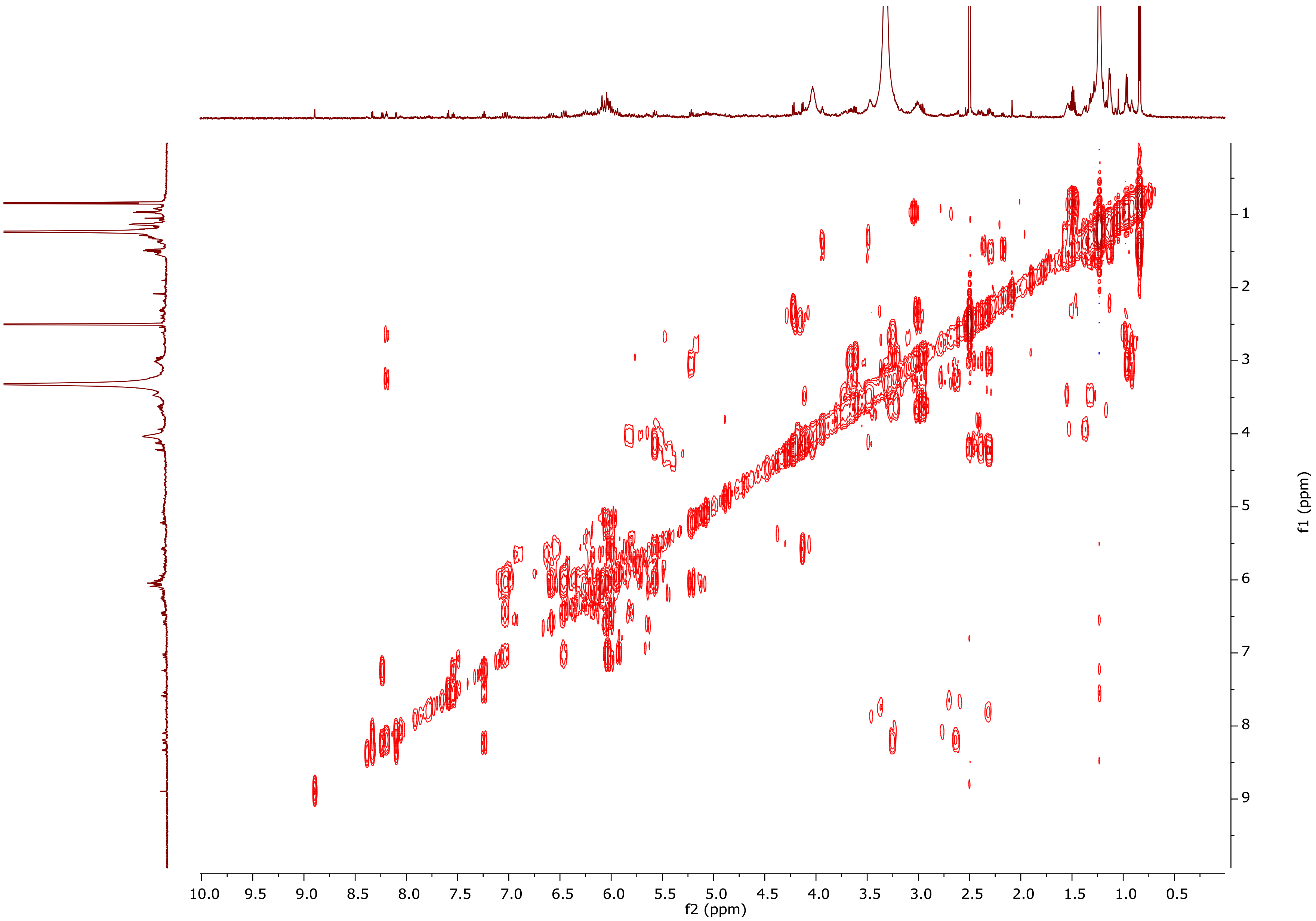

Figure S28. gCOSY NMR spectrum of macrotermycin E (**2**) in DMSO-d_6.

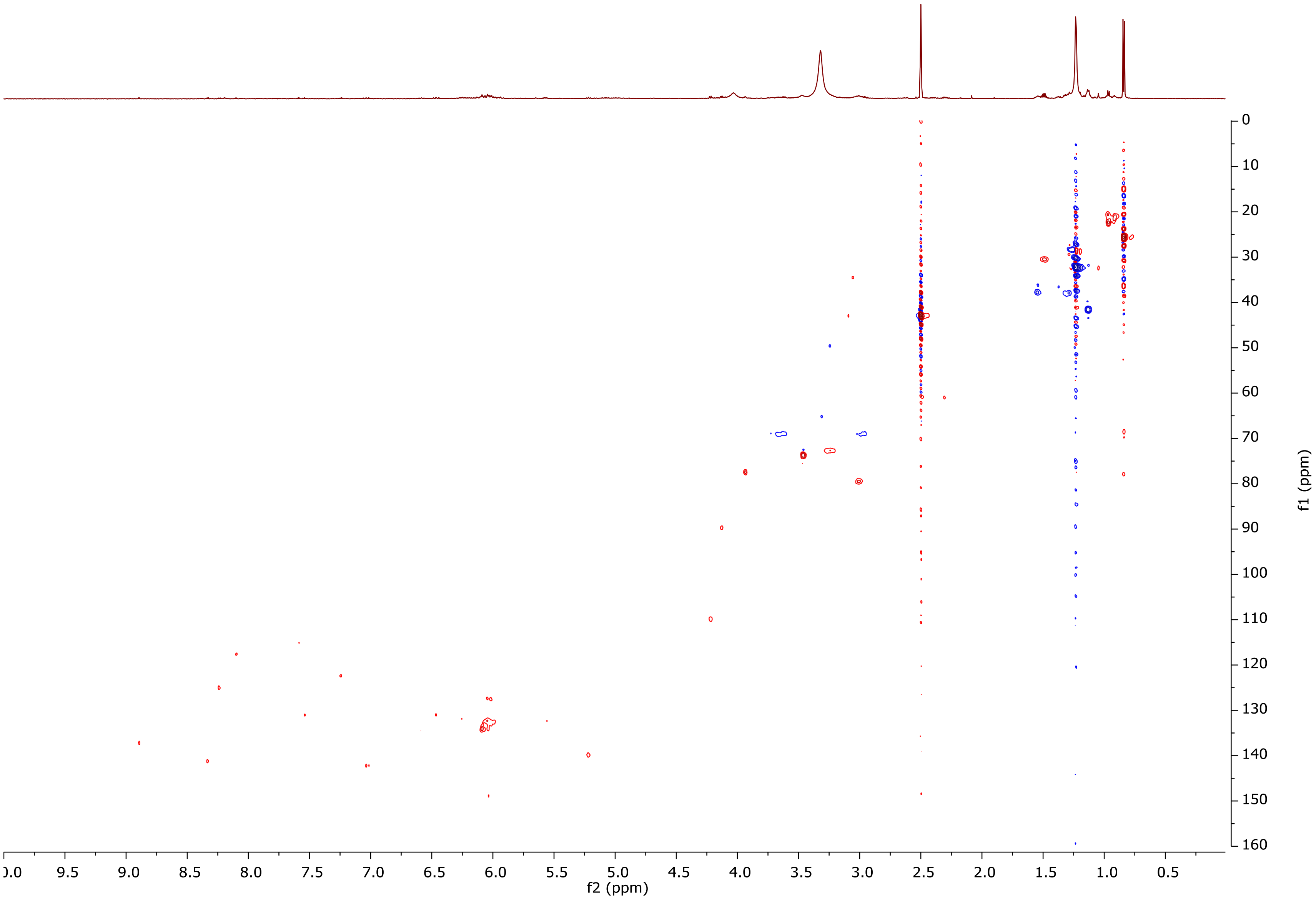

Figure S29. gHSQC NMR spectrum of macrotermycin E (**2**) in DMSO-d_6.

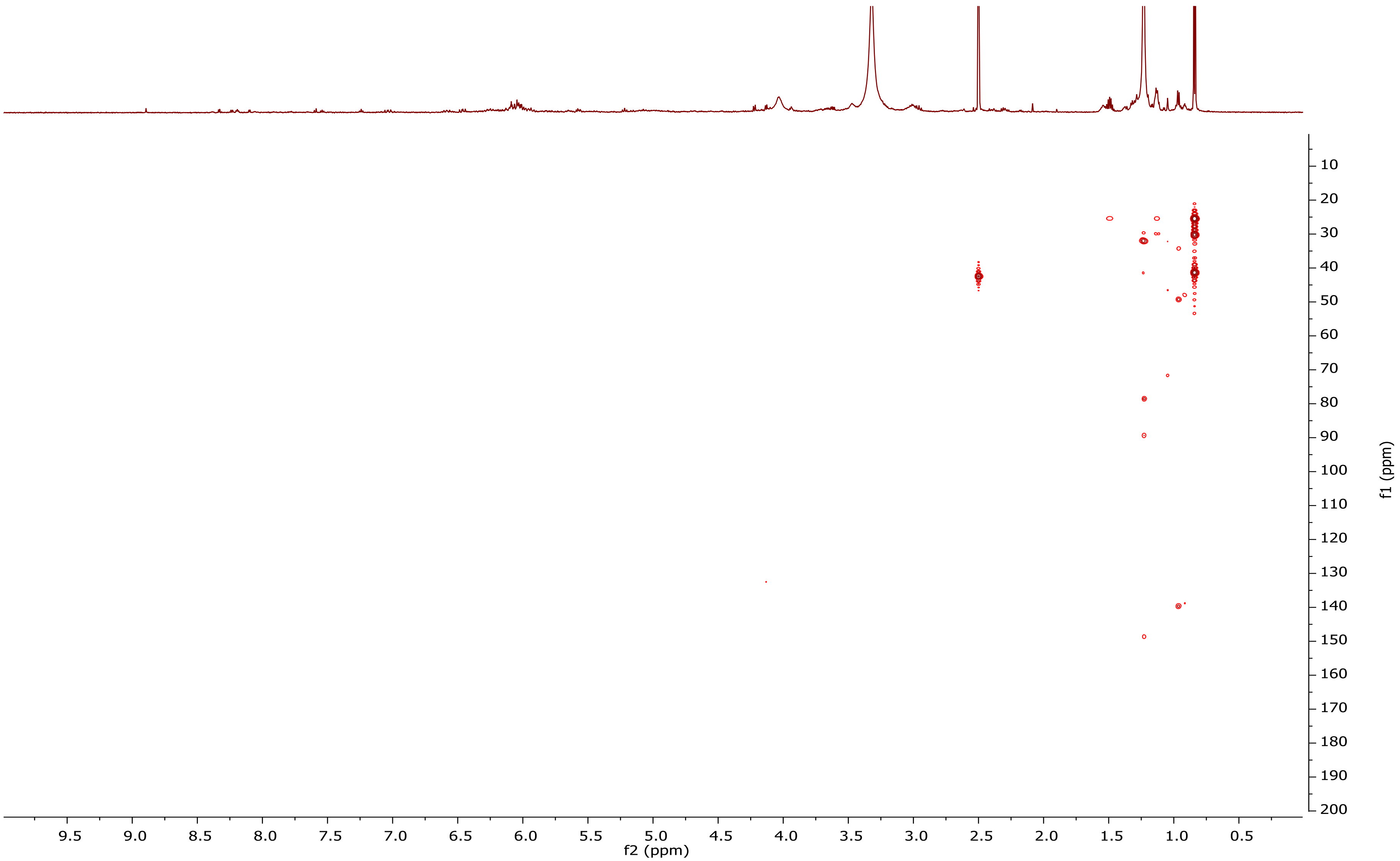

Figure S30. gHMBC NMR spectrum of macrotermycin E (**2**) in DMSO-d_6.

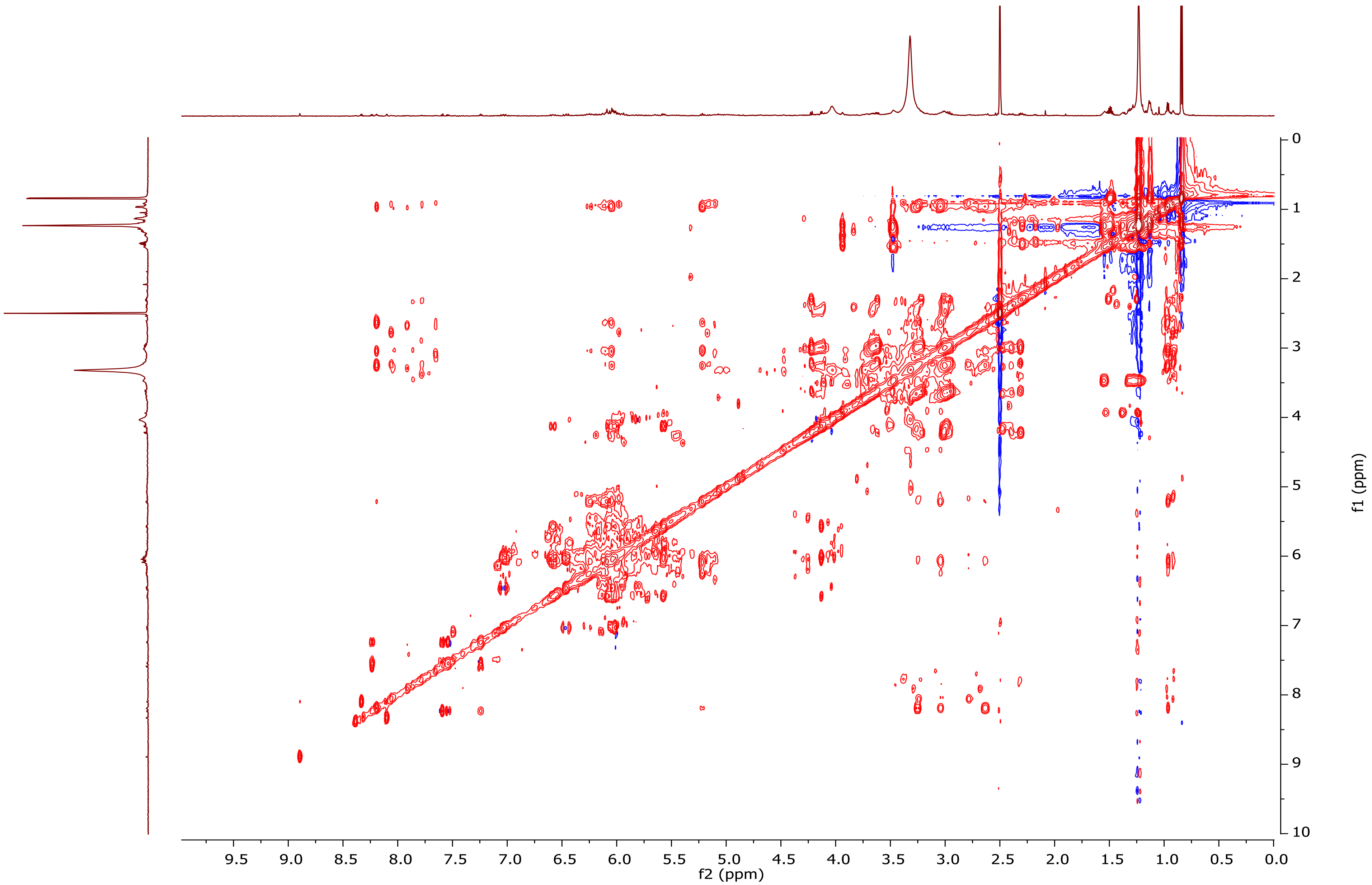

Figure S31. TOCSY NMR spectrum of macrotermycin E (**2**) in DMSO-d_6.

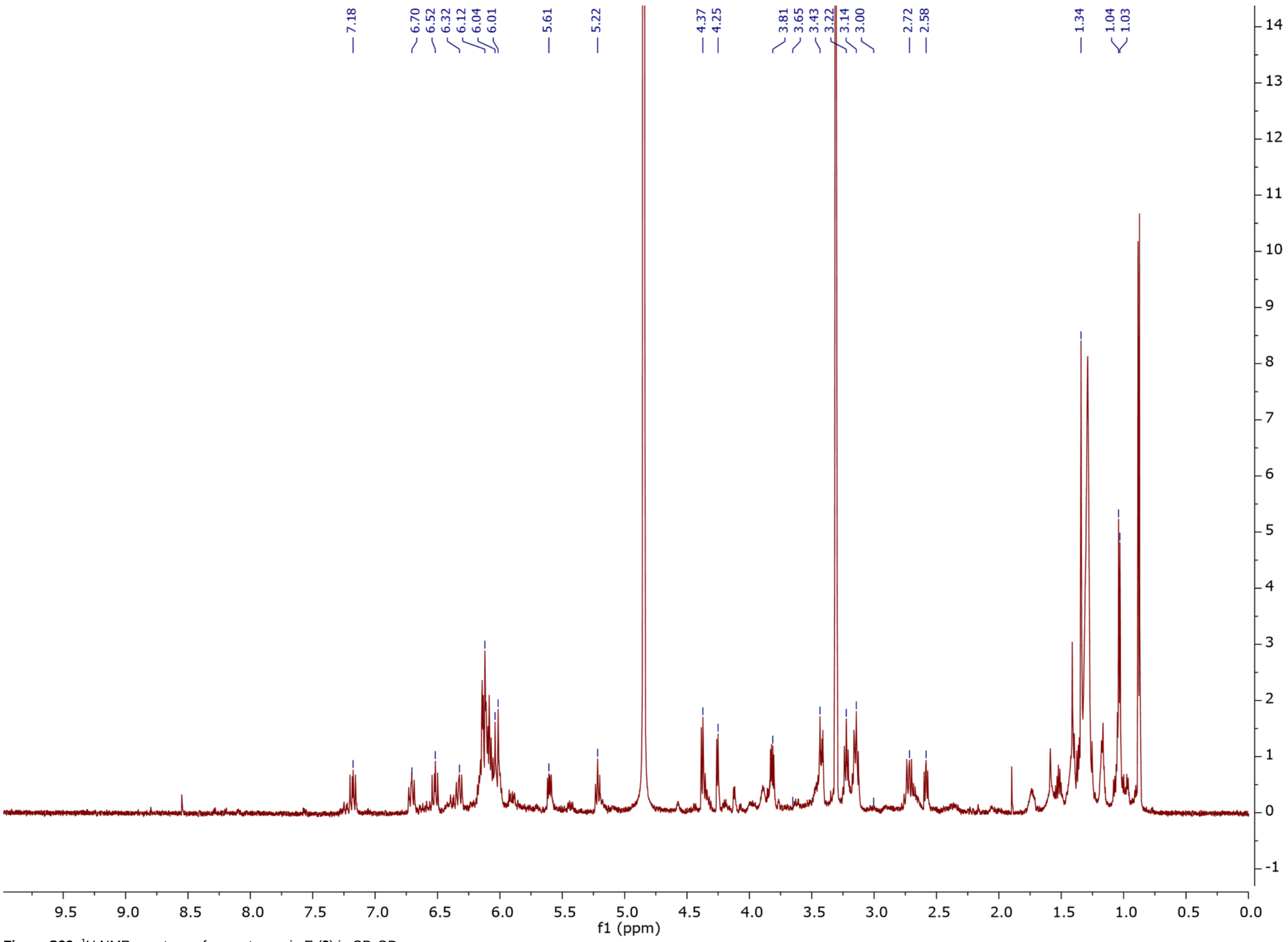

Figure S32. ^{1}H NMR spectrum of macrotermycin E (**2**) in CD_3OD.

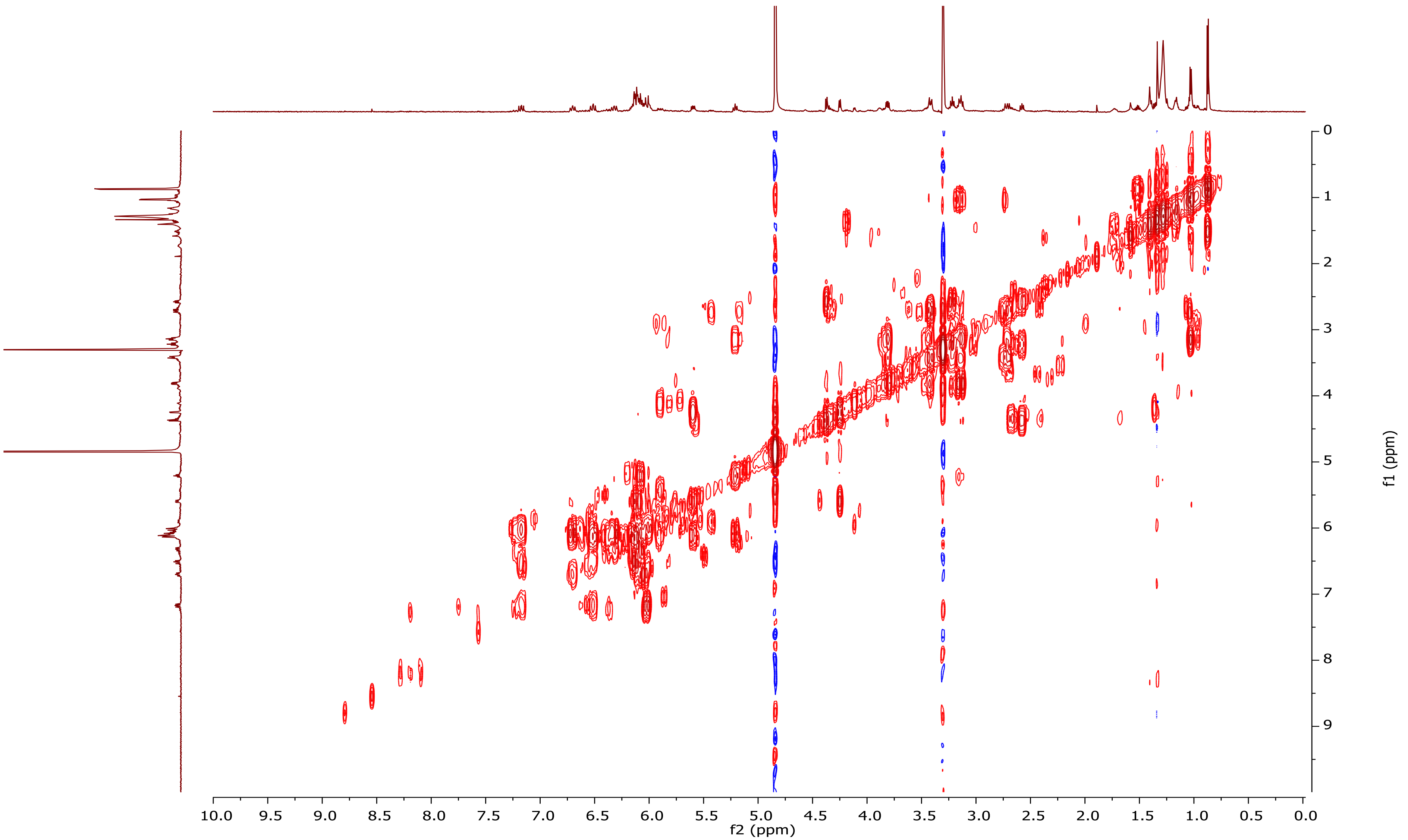

Figure S33. gCOSY NMR spectrum of macrotermycin E (**2**) in CD_3OD.

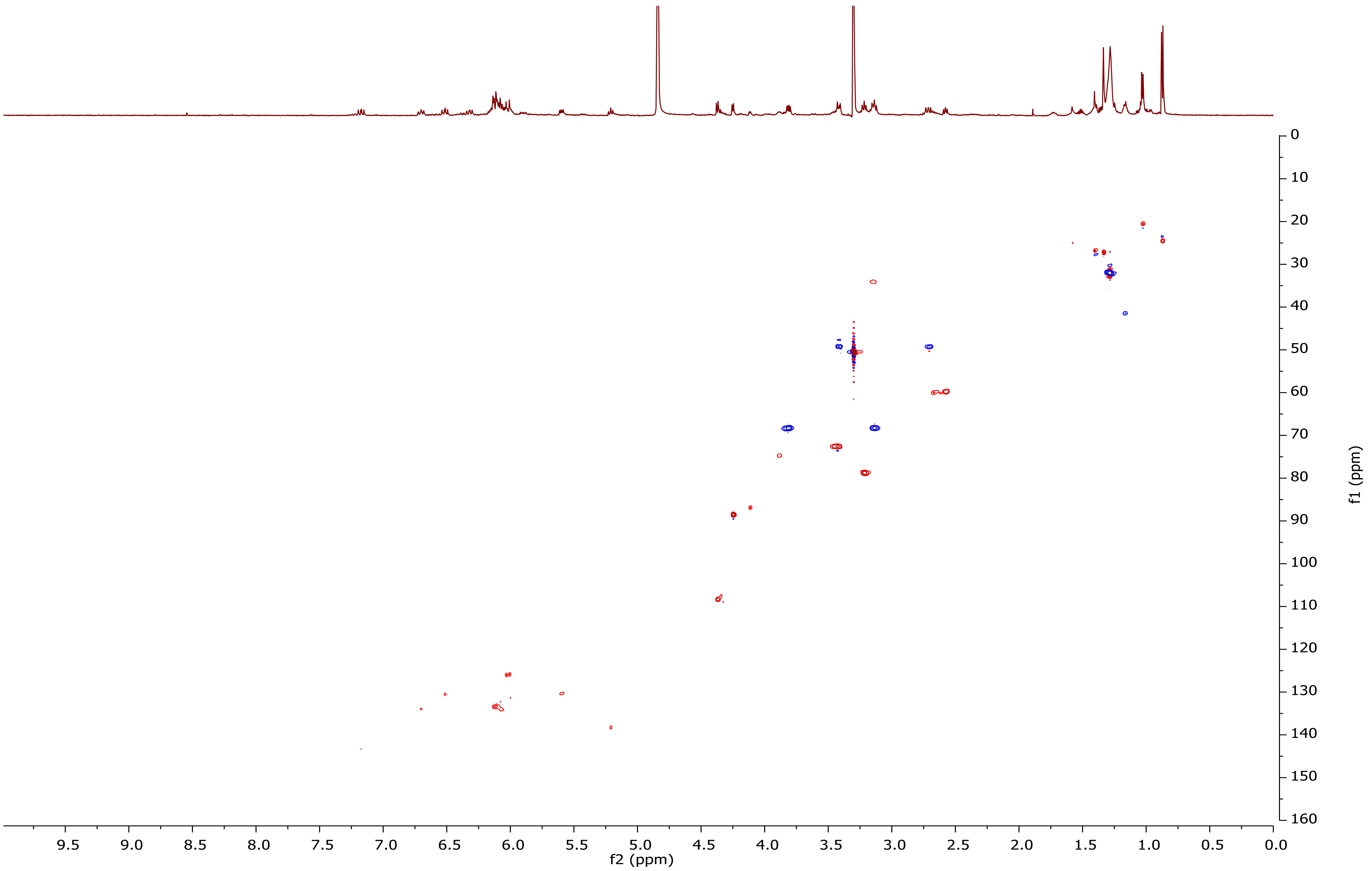

Figure S34. gHSQC NMR spectrum of macrotermycin E (**2**) in CD_3OD.

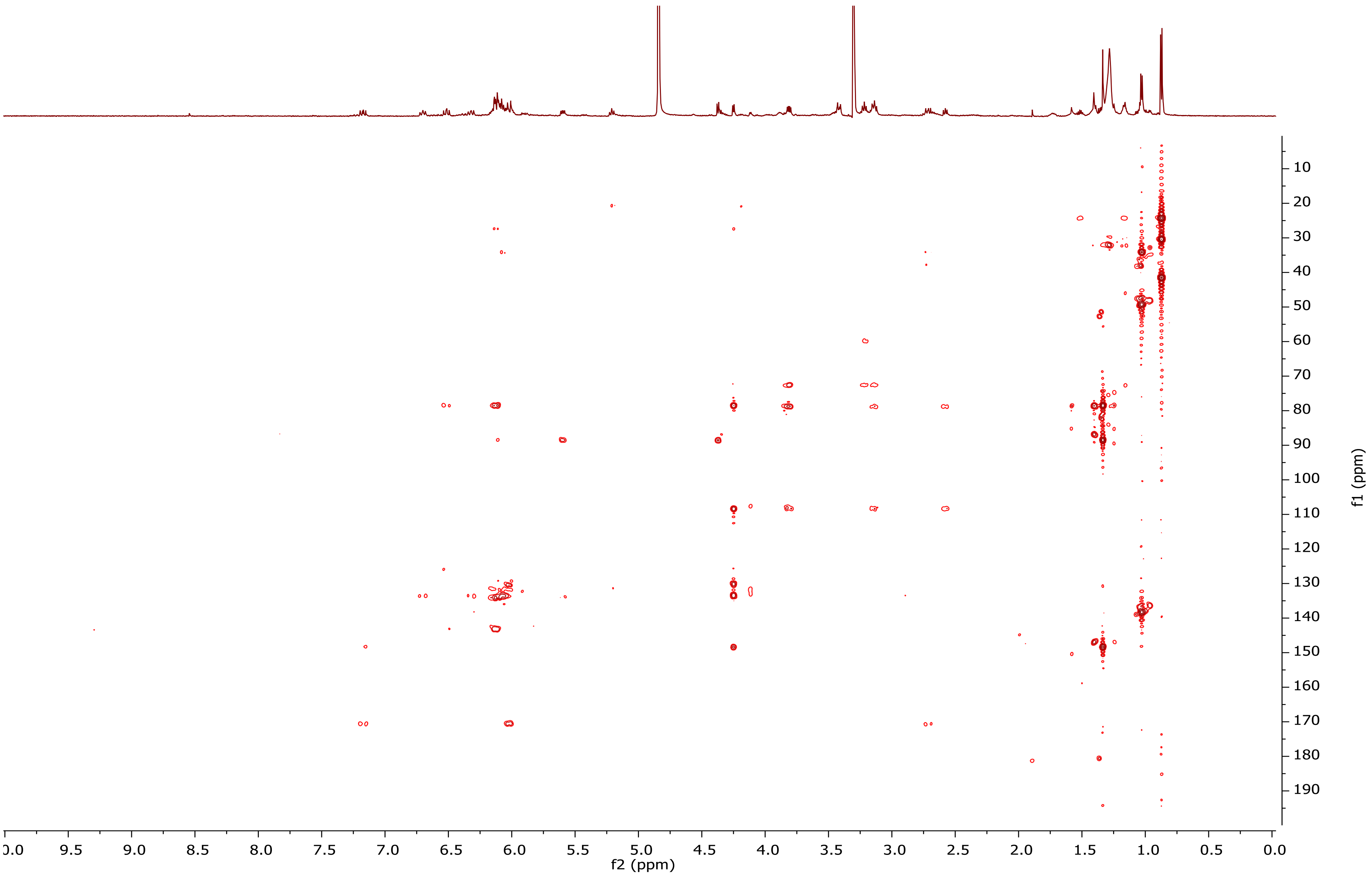

Figure S35. gHMBC NMR spectrum of macrotermycin E (**2**) in CD_3OD.

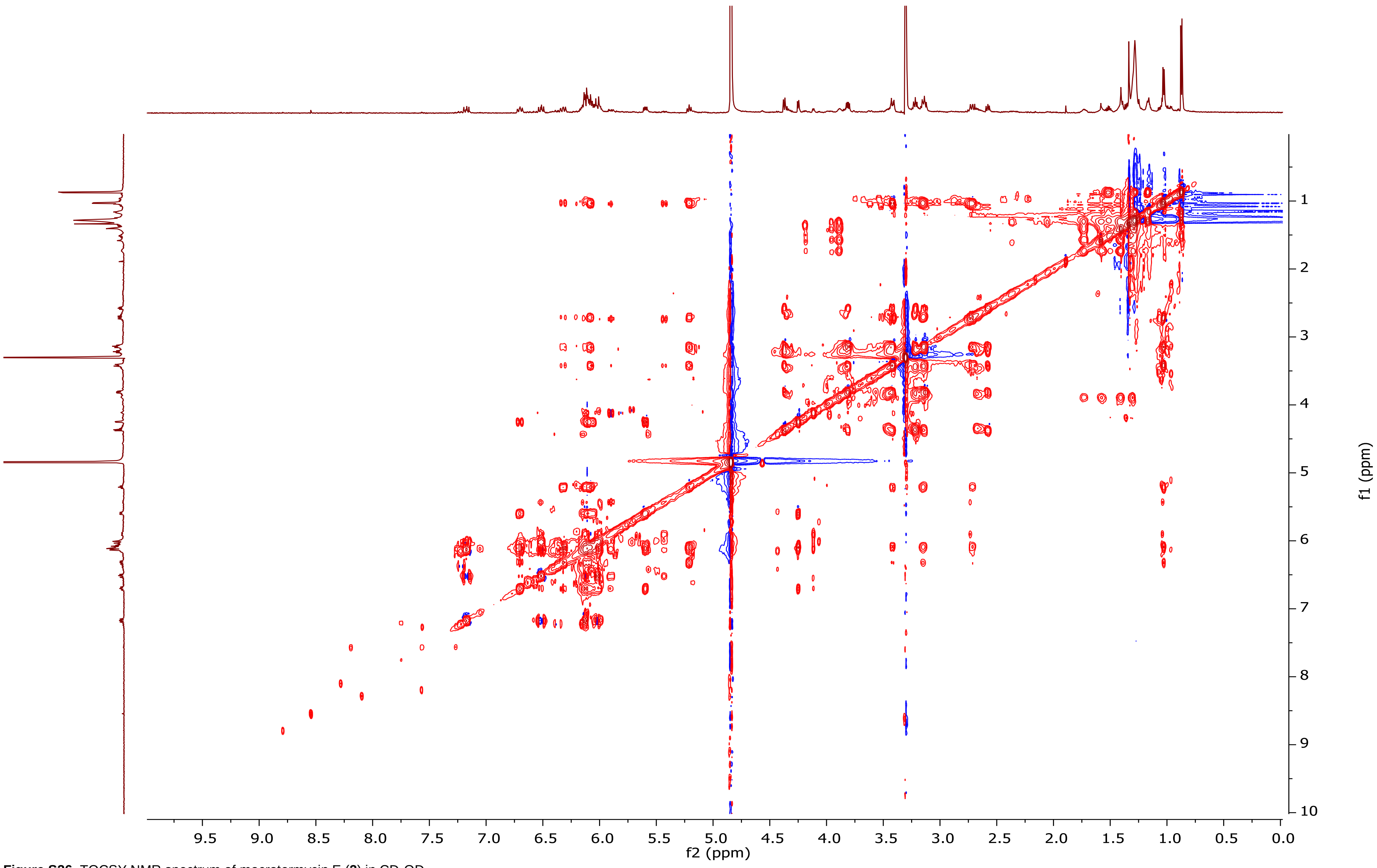

Figure S36. TOCSY NMR spectrum of macrotermycin E (**2**) in CD_3OD.

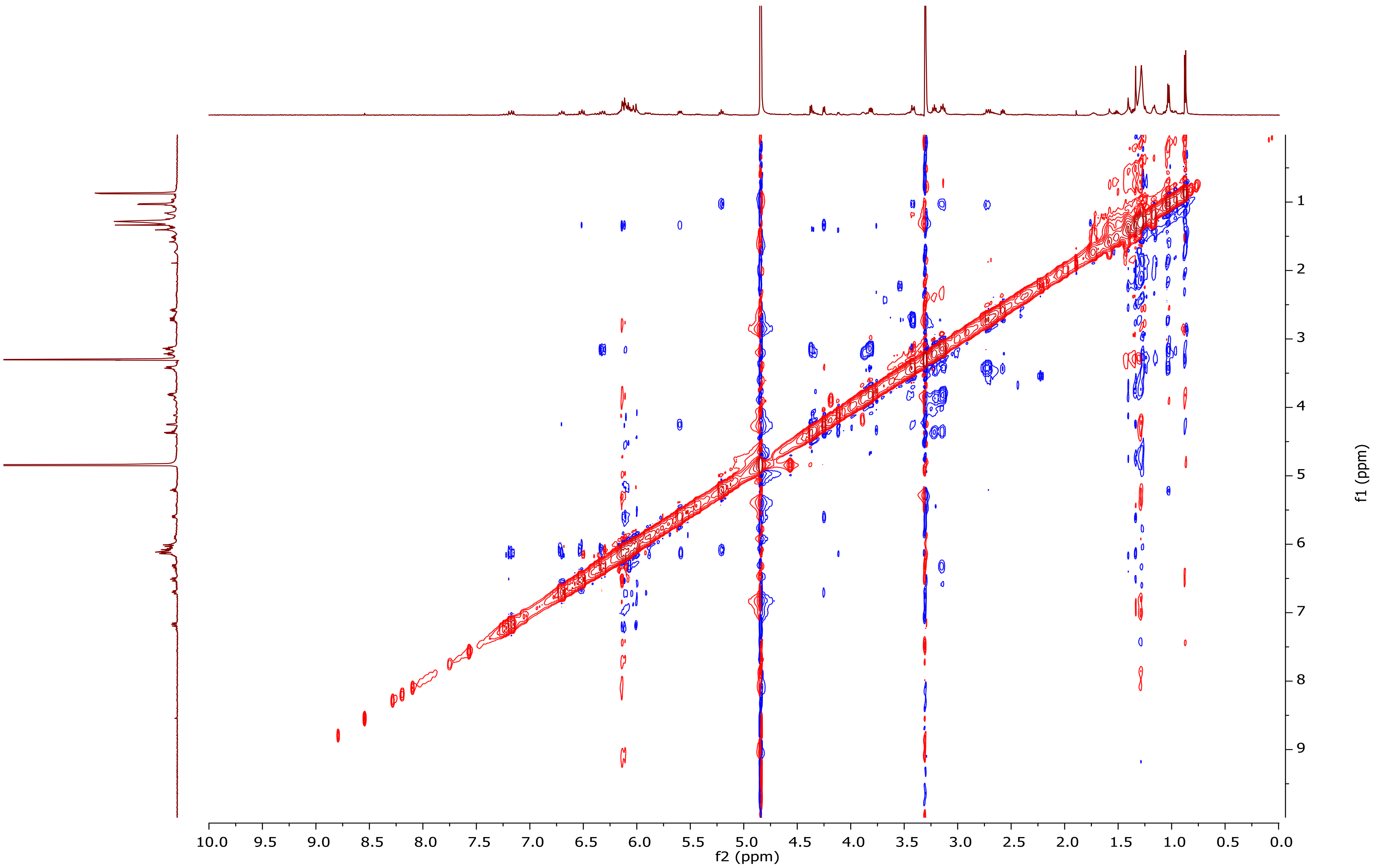

Figure S37. ROESY NMR spectrum of macrotermycin E (**2**) in CD_3OD.

Elemental Composition Report **Page 1**

Single Mass Analysis
Tolerance = 10.0 PPM / DBE: min = -1.5, max = 600.0
Element prediction: Off
Number of isotope peaks used for i-FIT = 3

Monoisotopic Mass, Even Electron Ions
49 formula(e) evaluated with 1 results within limits (all results (up to 1000) for each mass)
Elements Used:
C: 0-125 H: 0-250 N: 2-4 O: 5-7

Ki-hyun Kim, M39-compound 9 University of Illnois, SCS, Mass Spectrometry Lab Q-tof UE521
Qtof_50374 30 (2.245) AM (Cen,3, 80.00, Ar,15000.0,716.46,0.70,LS 3); Sm (SG, 2x3.00); Cm (30:34) 1: TOF MS ES+
4.21e+002

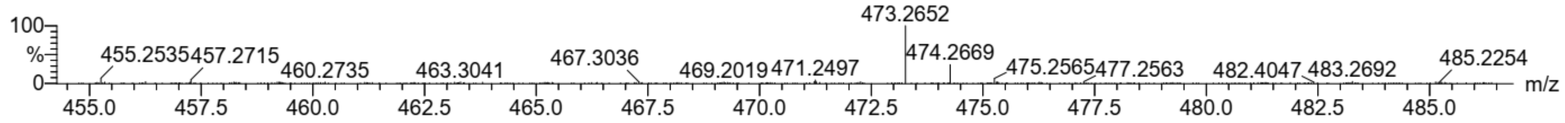

Minimum:				-1.5		
Maximum:		5.0	10.0	600.0		
Mass	Calc. Mass	mDa	PPM	DBE	i-FIT	Formula
473.2652	473.2652	0.0	0.0	9.5	1.6	C26 H37 N2 O6

Figure S38. HRMS-spectrum of macrotermycin E (**2**)

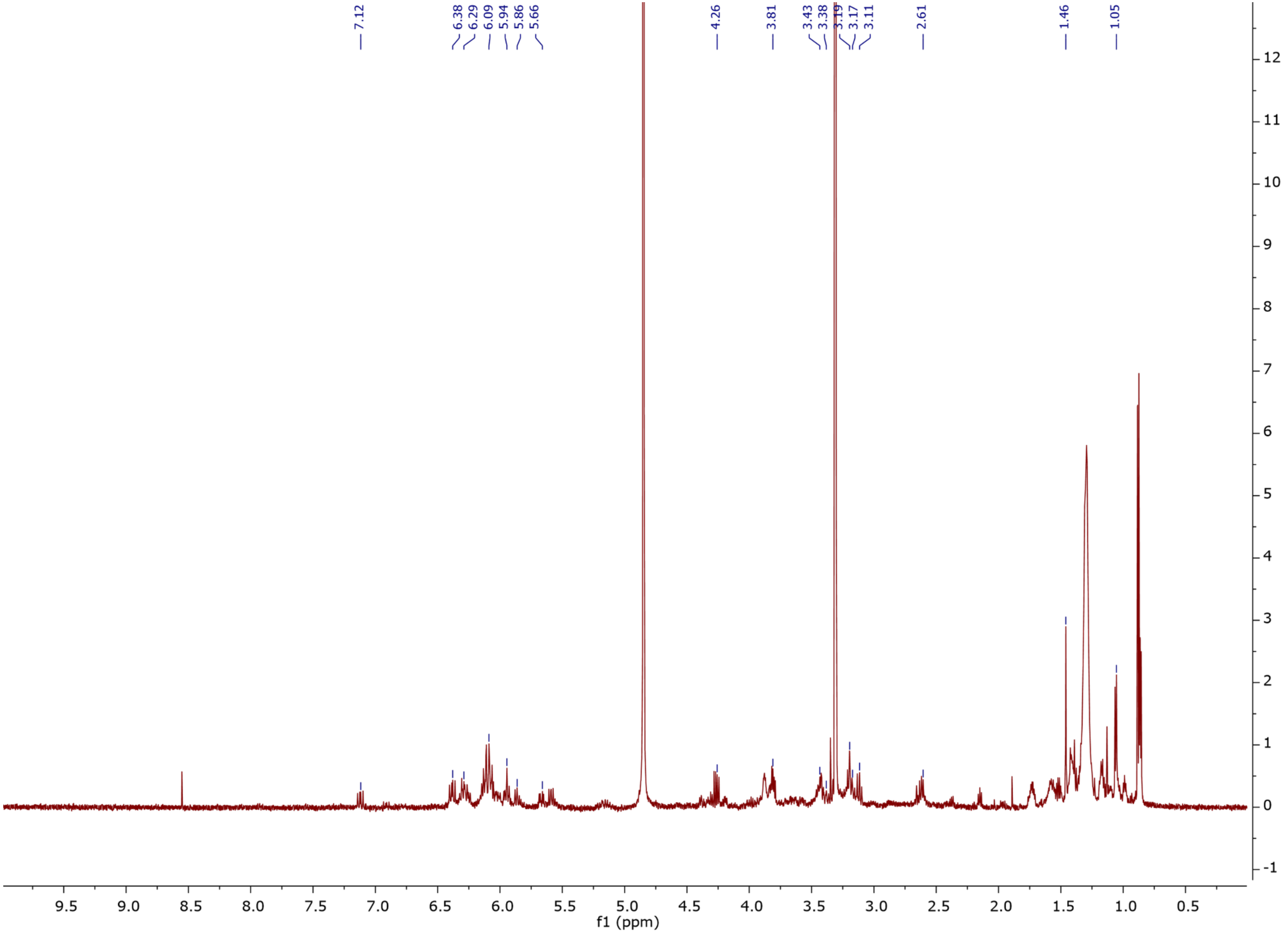

Figure S39. ^{1}H NMR spectrum of macrotermycin F (**3**) in CD_3OD.

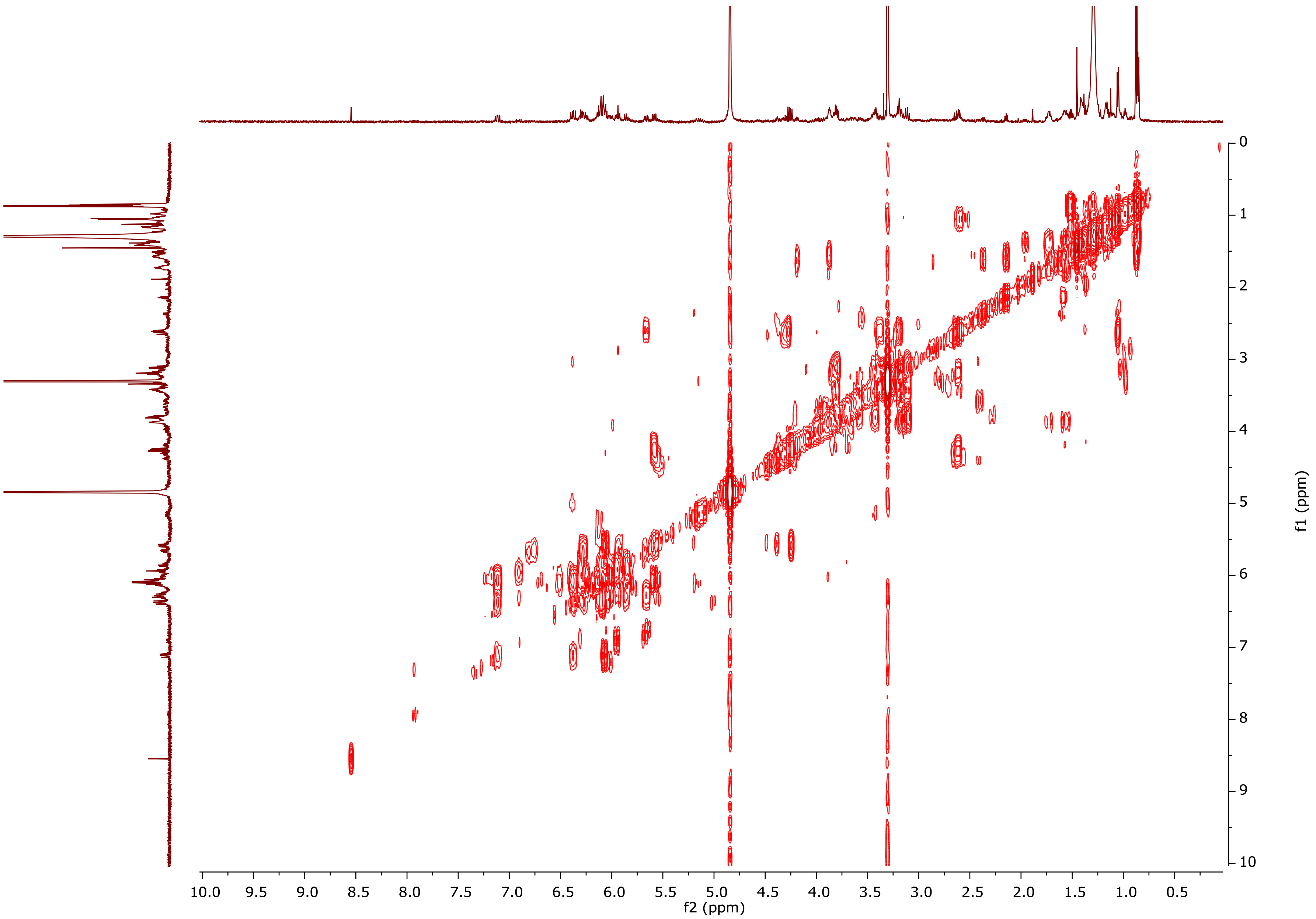

Figure S40. gCOSY NMR spectrum of macrotermycin F (**3**) in CD_3OD.

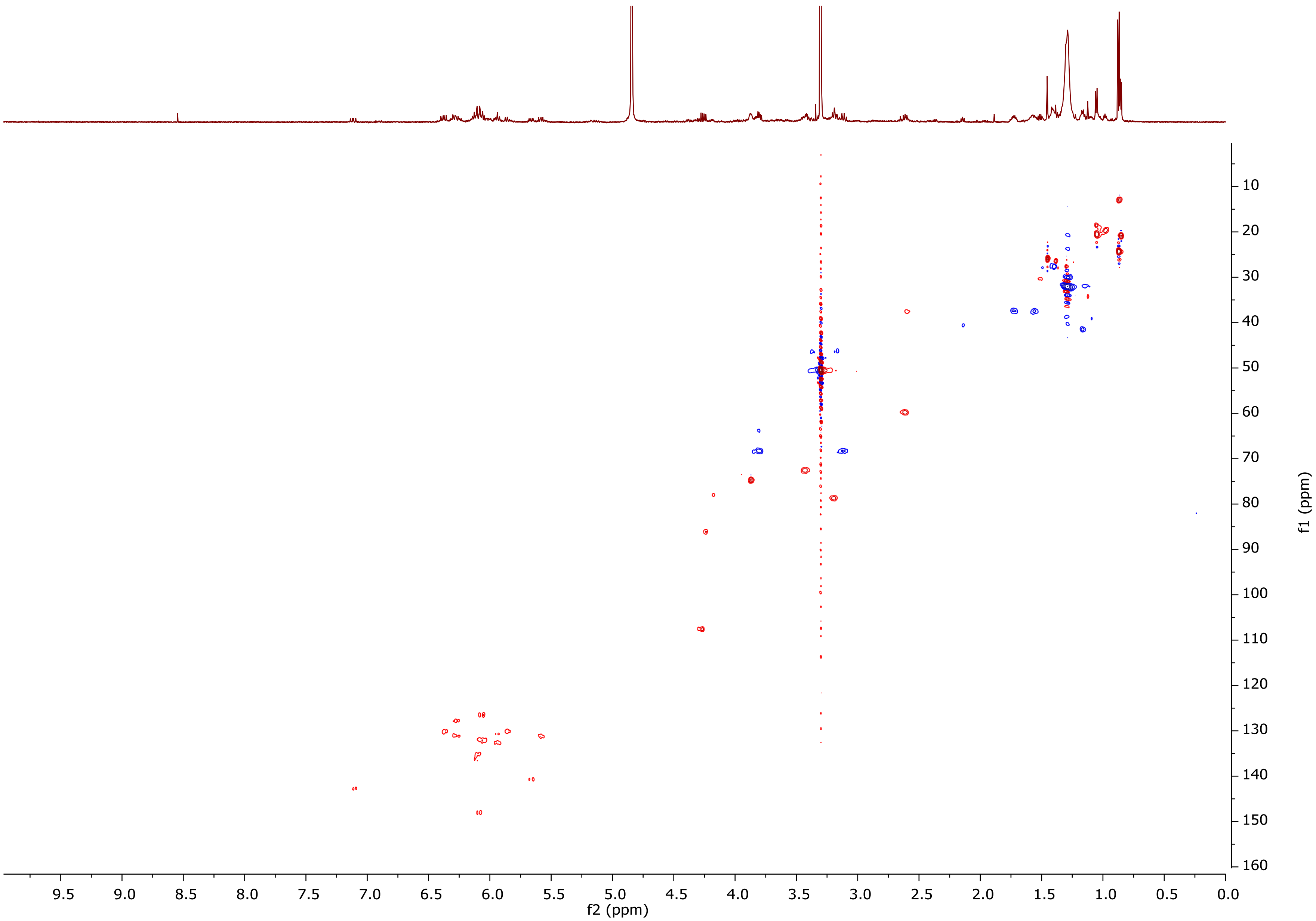

Figure S41. gHSQC NMR spectrum of macrotermycin F (**3**) in CD_3OD.

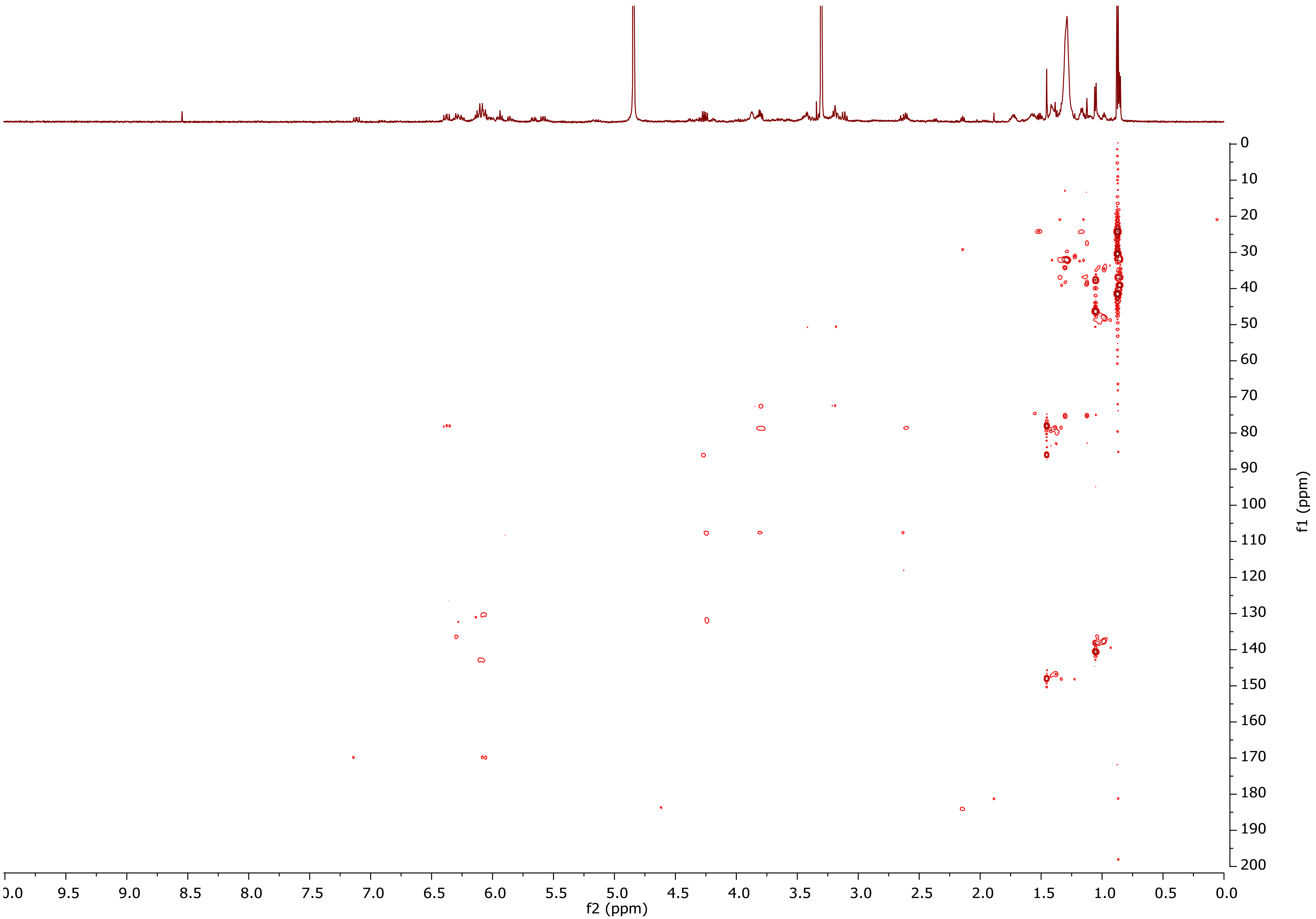

Figure S42. gHMBC NMR spectrum of macrotermycin F (**3**) in CD_3OD.

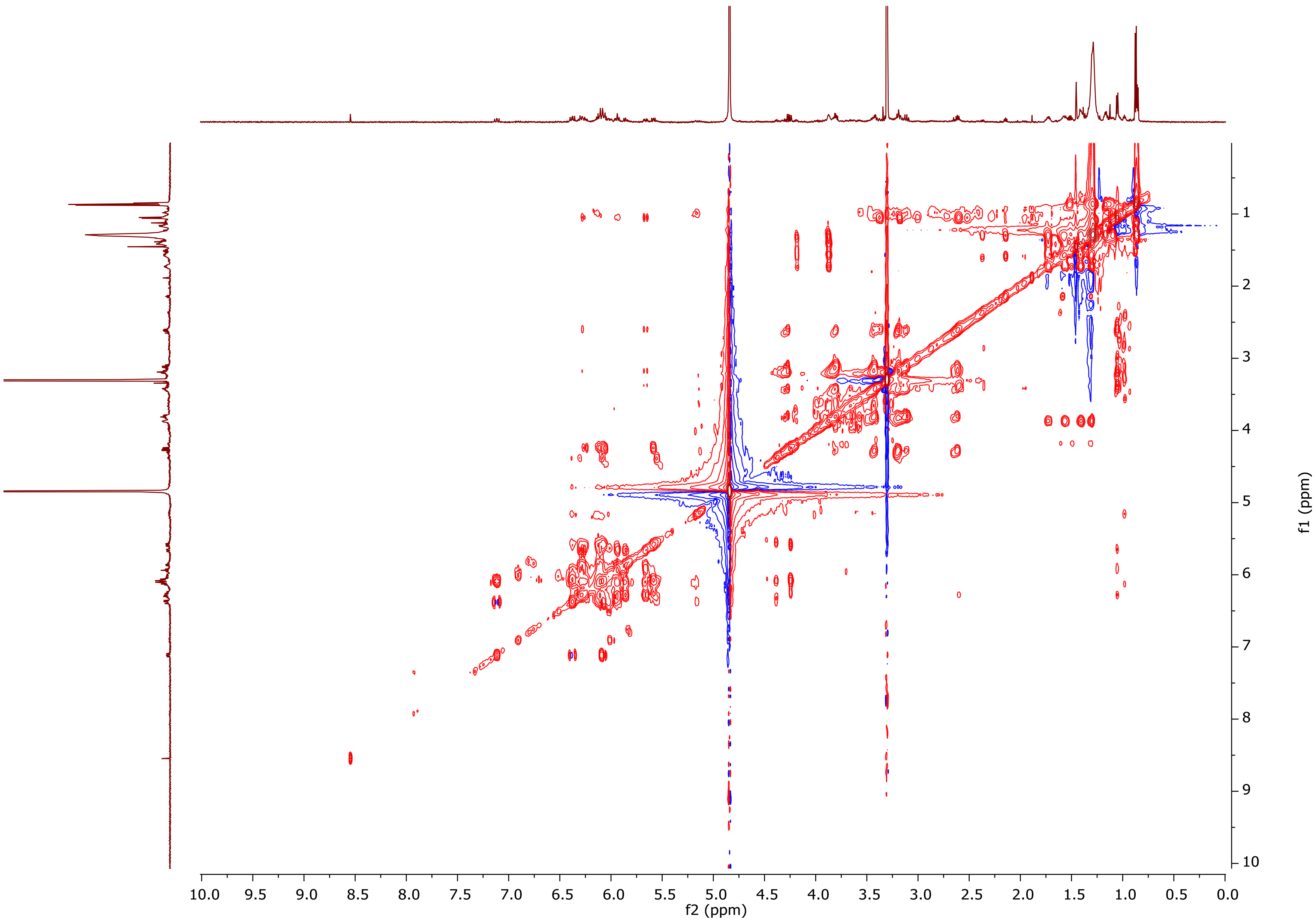

Figure S43. TOCSY NMR spectrum of macrotermycin F (**3**) in CD_3OD.

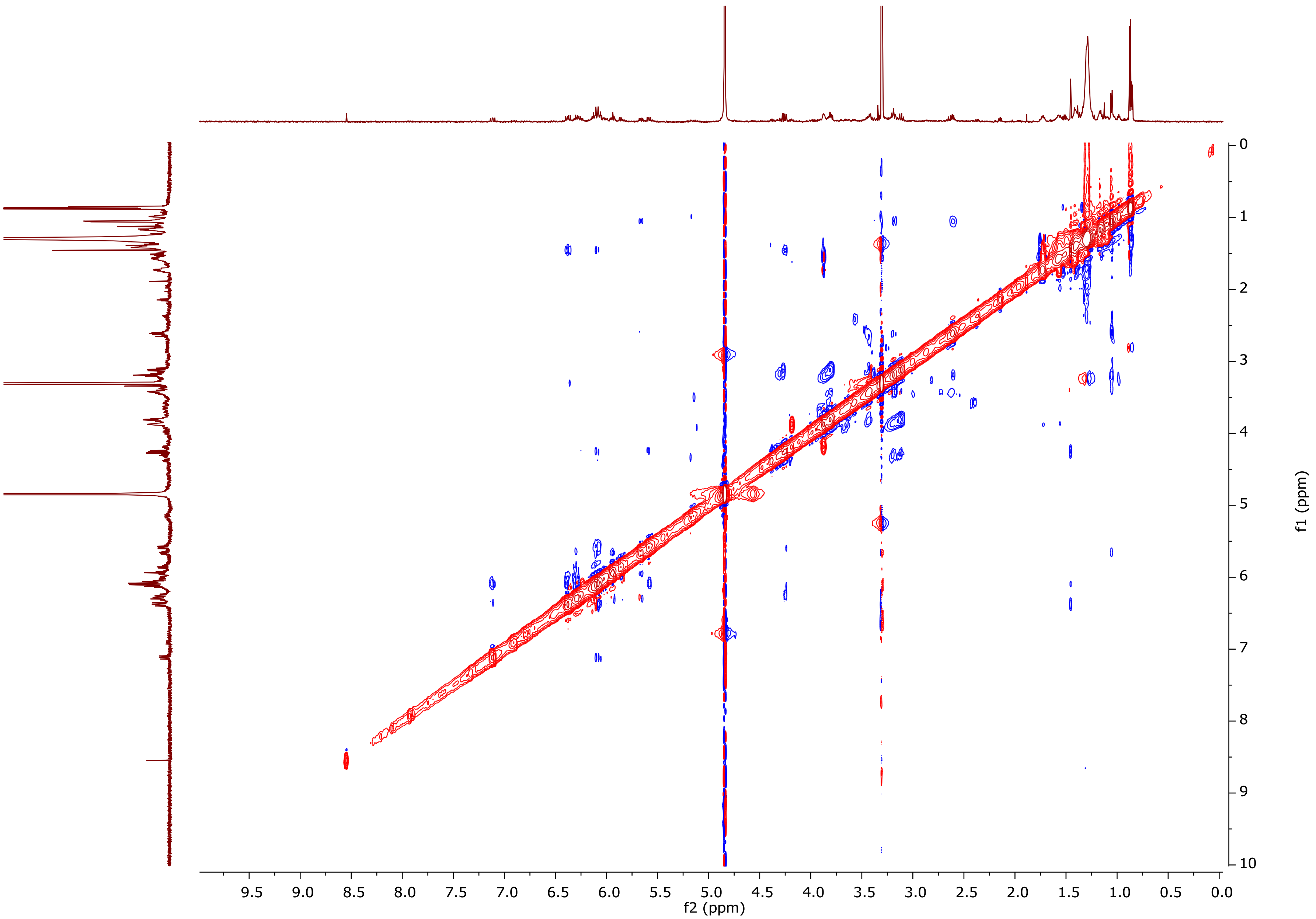

Figure S44. ROESY NMR spectrum of macrotermycin F (**3**) in CD_3OD.

Elemental Composition Report

Single Mass Analysis
Tolerance = 10.0 PPM / DBE: min = -1.5, max = 600.0
Element prediction: Off
Number of isotope peaks used for i-FIT = 3

Monoisotopic Mass, Even Electron Ions
49 formula(e) evaluated with 1 results within limits (all results (up to 1000) for each mass)
Elements Used:
C: 0-125 H: 0-250 N: 2-4 O: 5-7

Ki-hyun Kim, M39-compound 11 University of Illnois, SCS, Mass Spectrometry Lab Q-tof UE521
Qtof_50372 28 (2.096) AM (Cen,3, 80.00, Ar,15000.0,716.46,0.70,LS 3); Sm (SG, 2x3.00); Cm (28:29) 1: TOF MS ES+ 1.50e+003

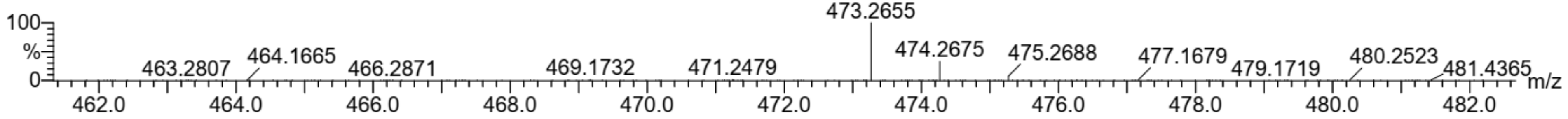

Minimum:				-1.5		
Maximum:		5.0	10.0	600.0		
Mass	Calc. Mass	mDa	PPM	DBE	i-FIT	Formula
473.2655	473.2652	0.3	0.6	9.5	2.4	C26 H37 N2 O6

Figure S45. HRMS of macrotermycin F (**3**).

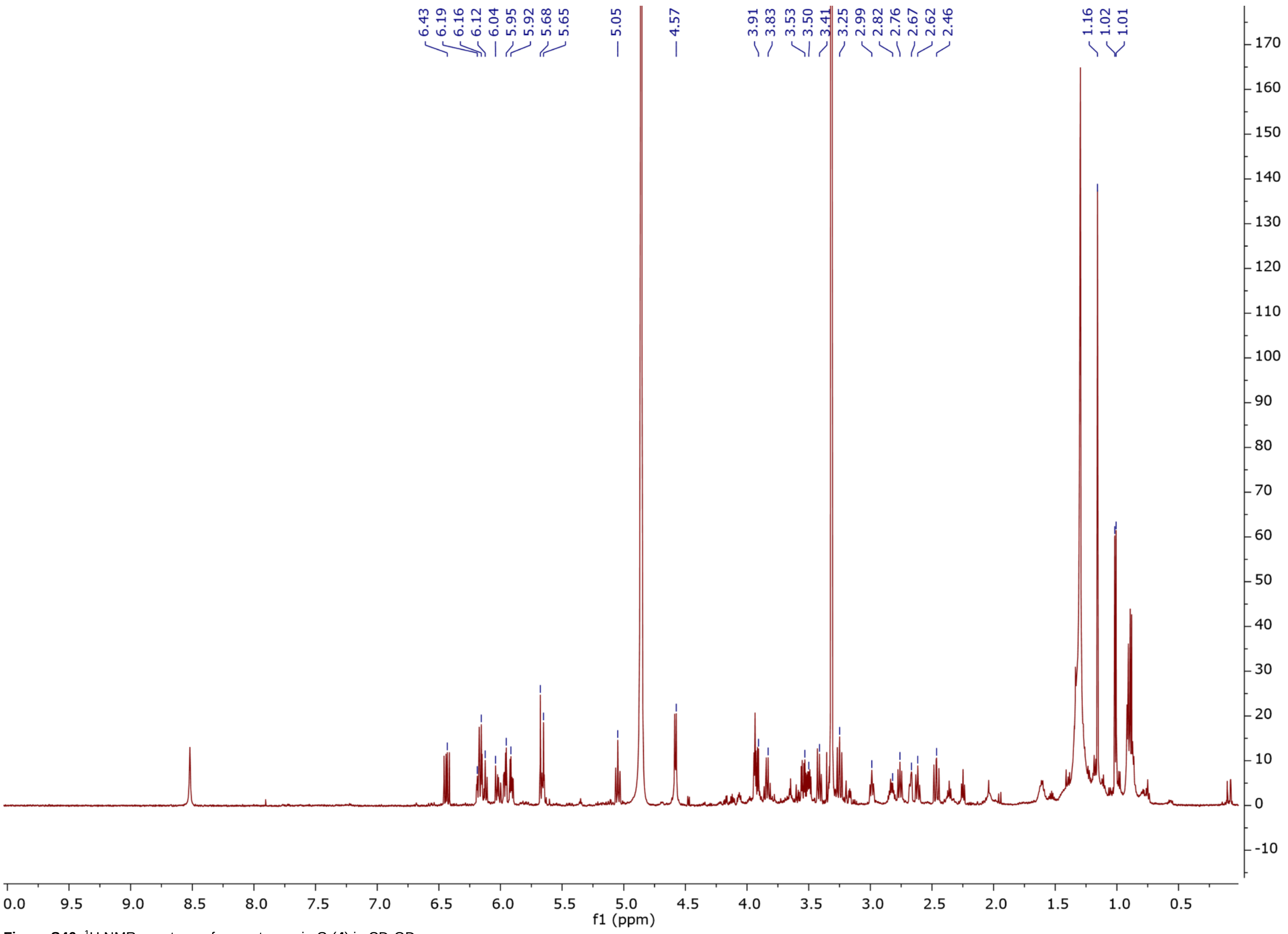

Figure S46. ^{1}H NMR spectrum of macrotermycin G (**4**) in CD_3OD.

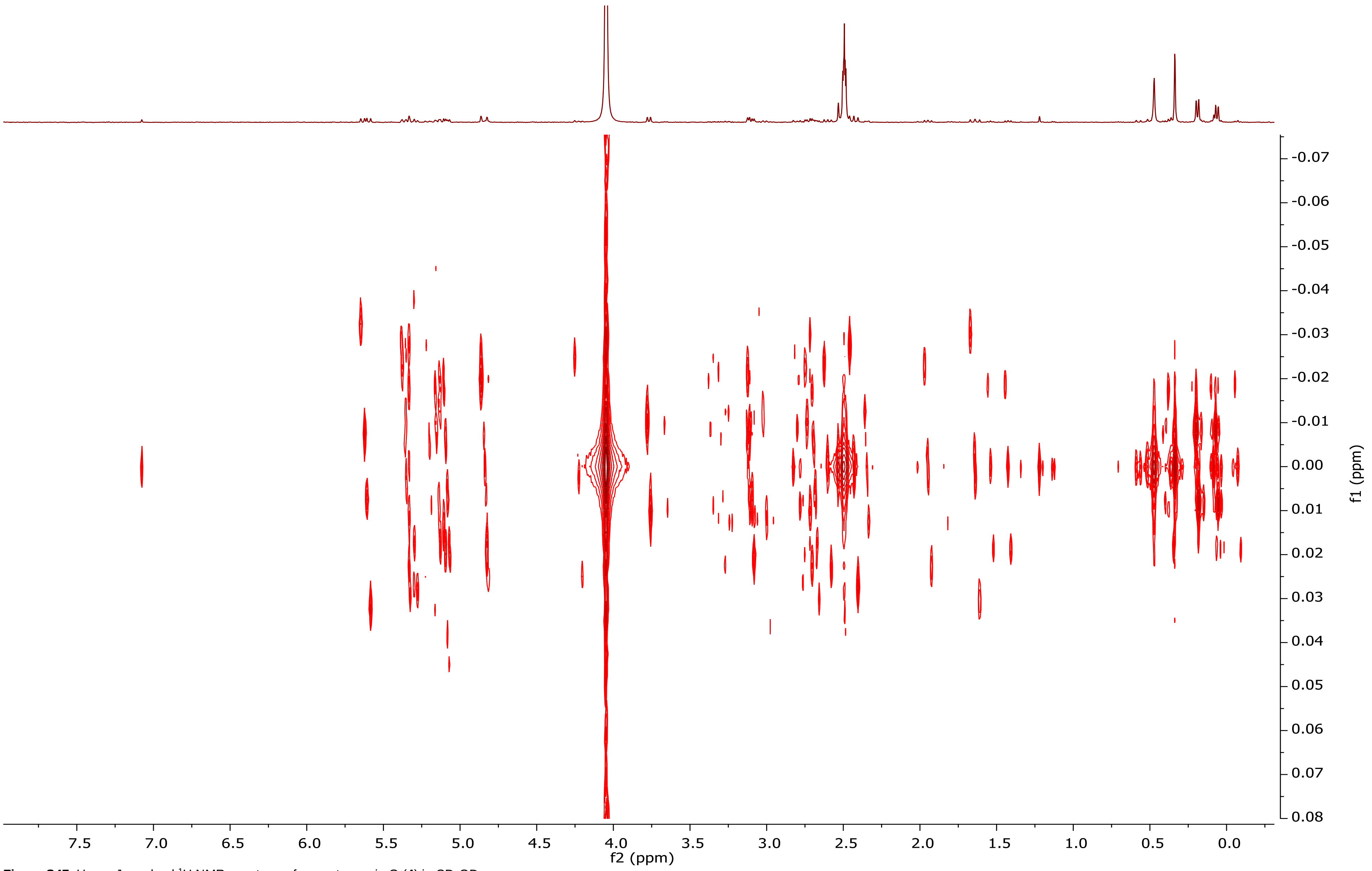

Figure S47. Homo *J*-resolved ^{1}H NMR spectrum of macrotermycin G (**4**) in CD_3OD.

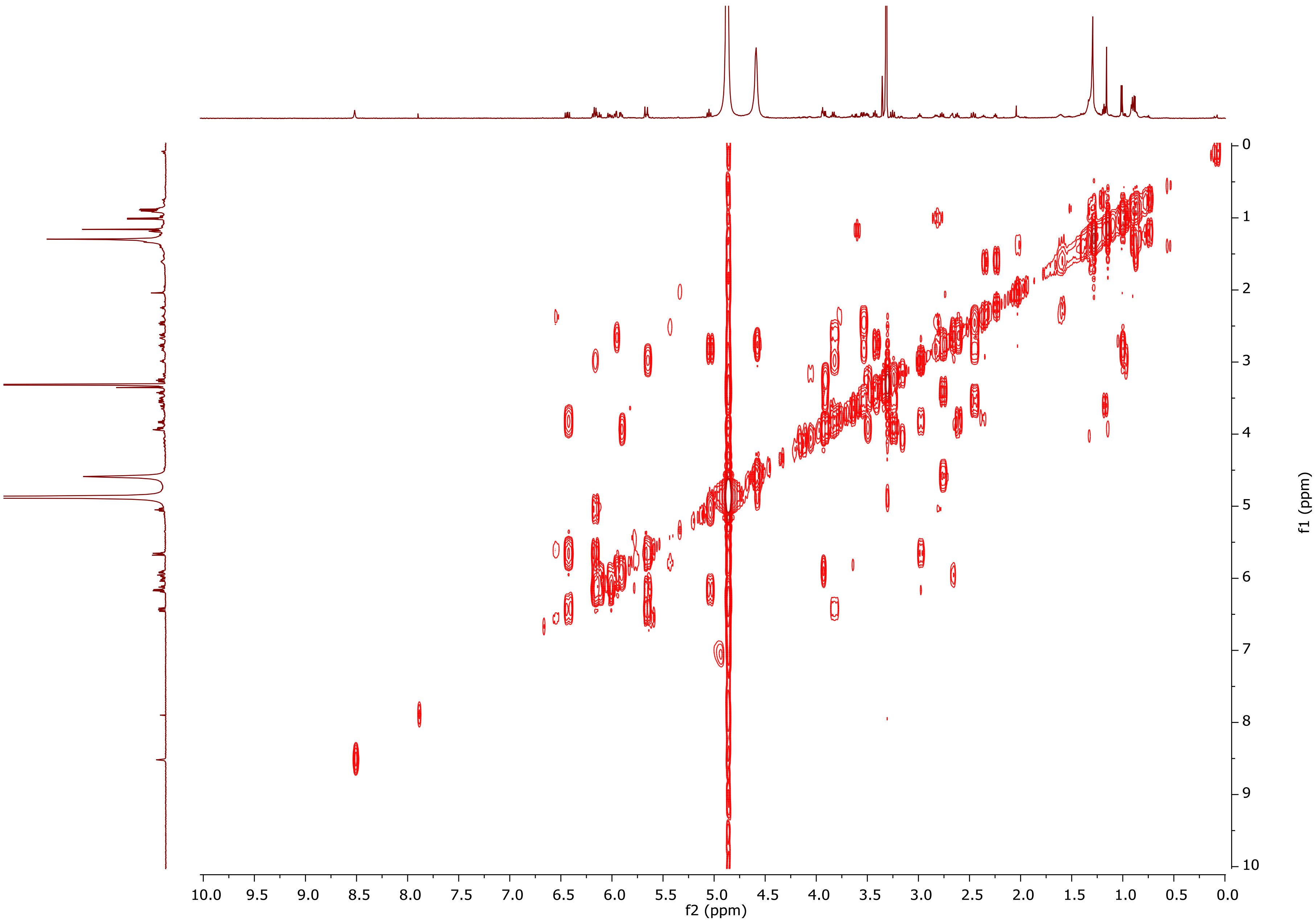

Figure S48. gCOSY NMR spectrum of macrotermycin G (**4**) in CD_3OD.

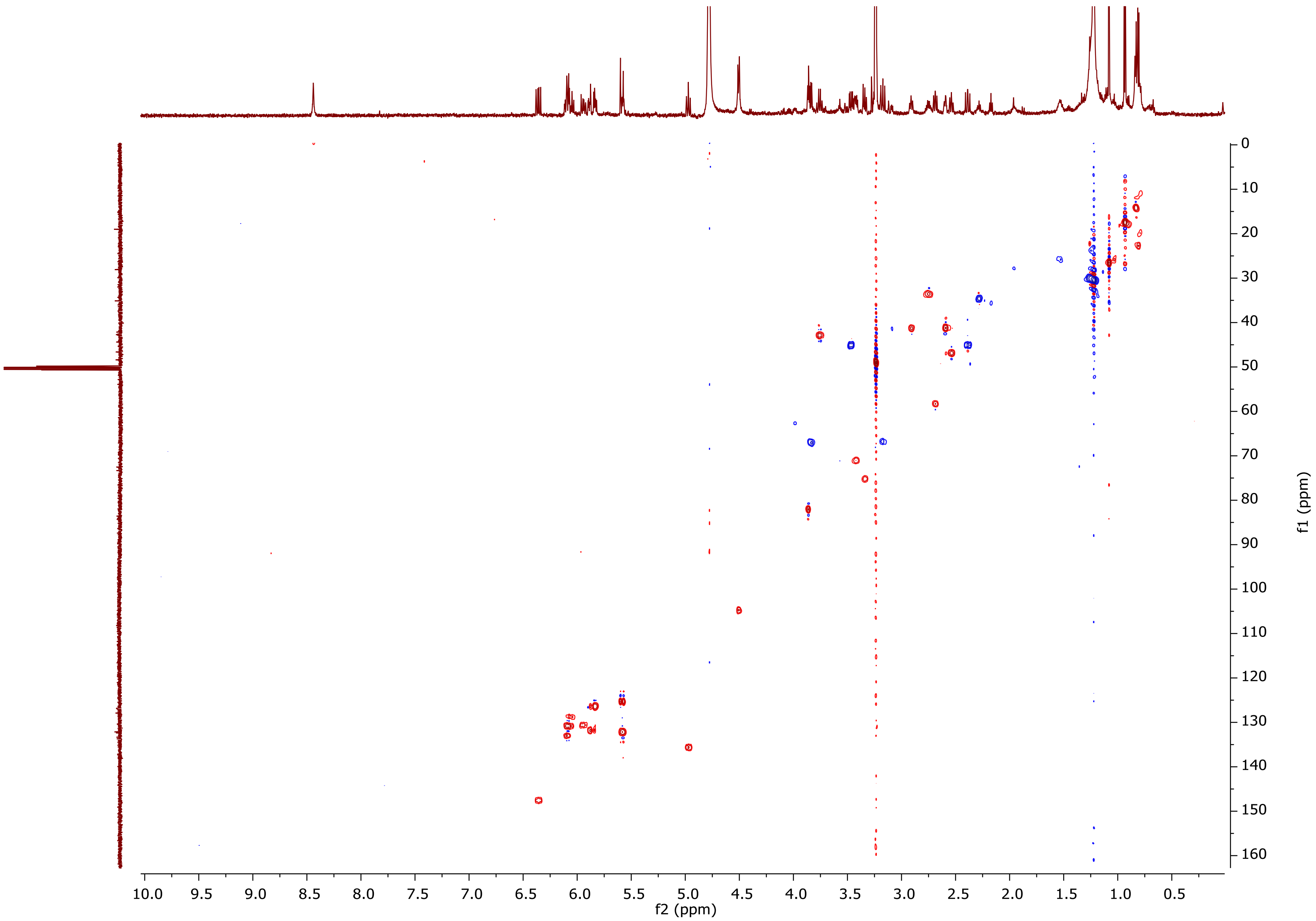

Figure S49. HSQC NMR spectrum of macrotermycin G (**4**) in CD_3OD.

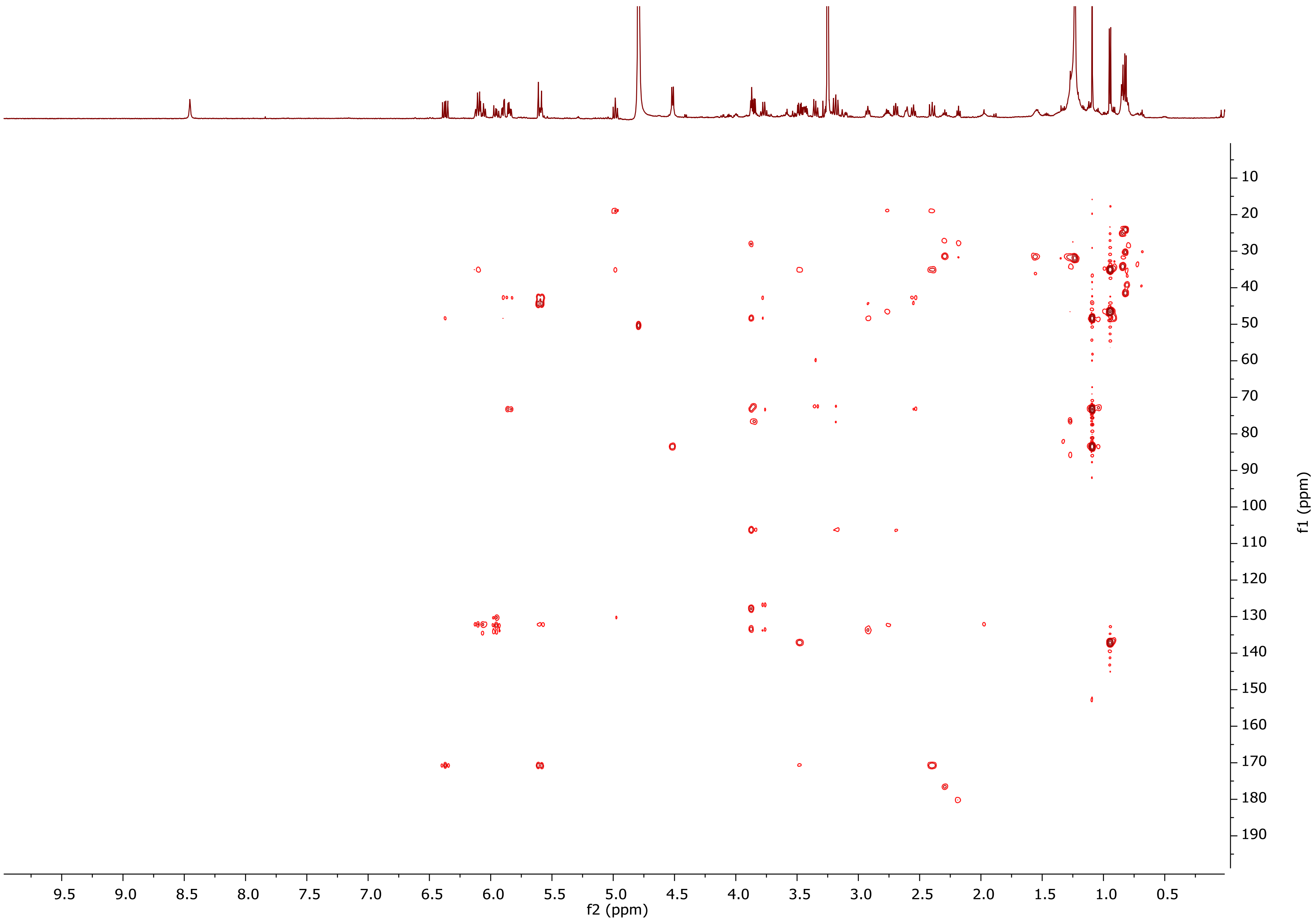

Figure S50. gHMBC NMR spectrum of macrotermycin G (**4**) in CD_3OD.

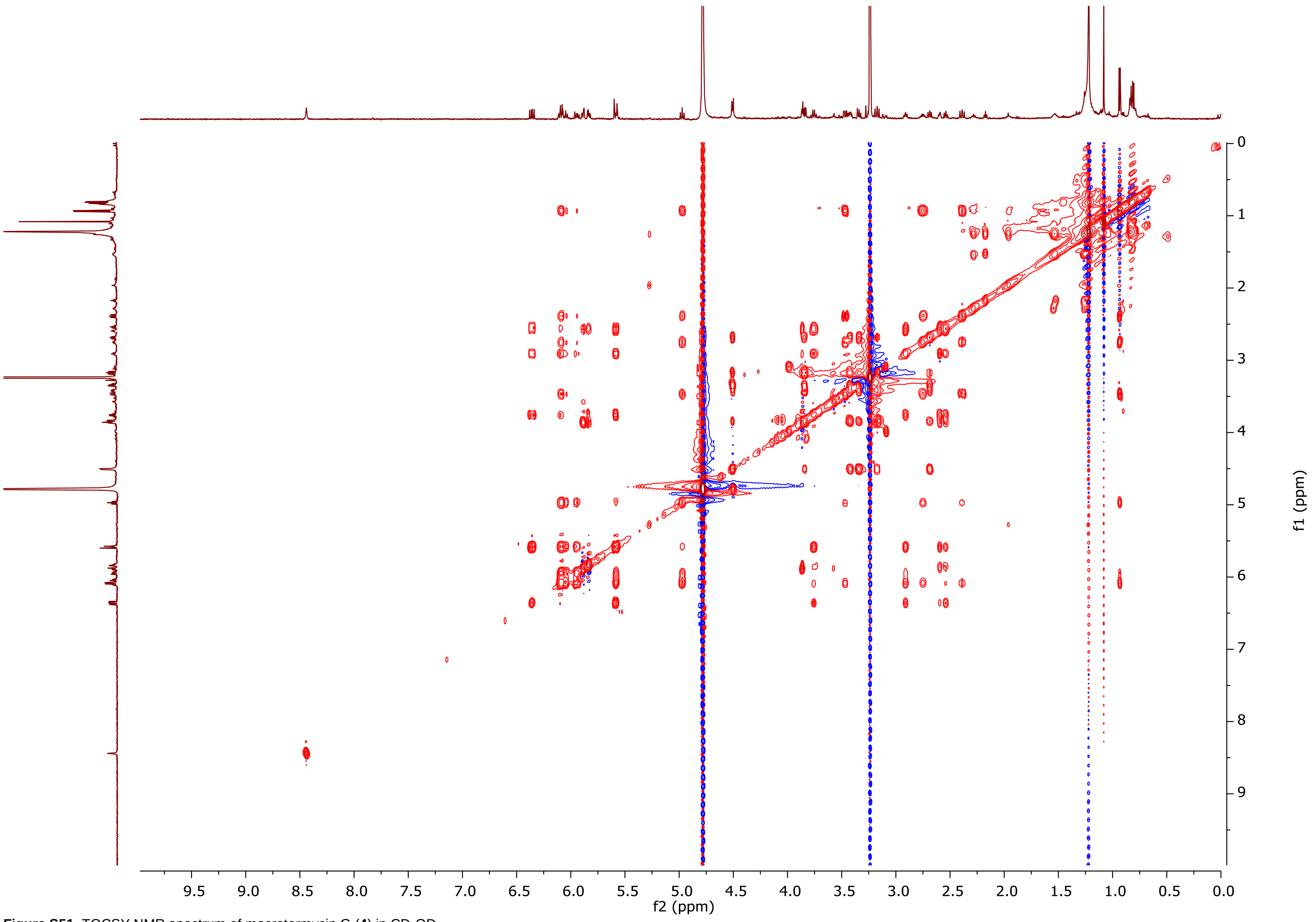

Figure S51. TOCSY NMR spectrum of macrotermycin G (**4**) in CD_3OD.

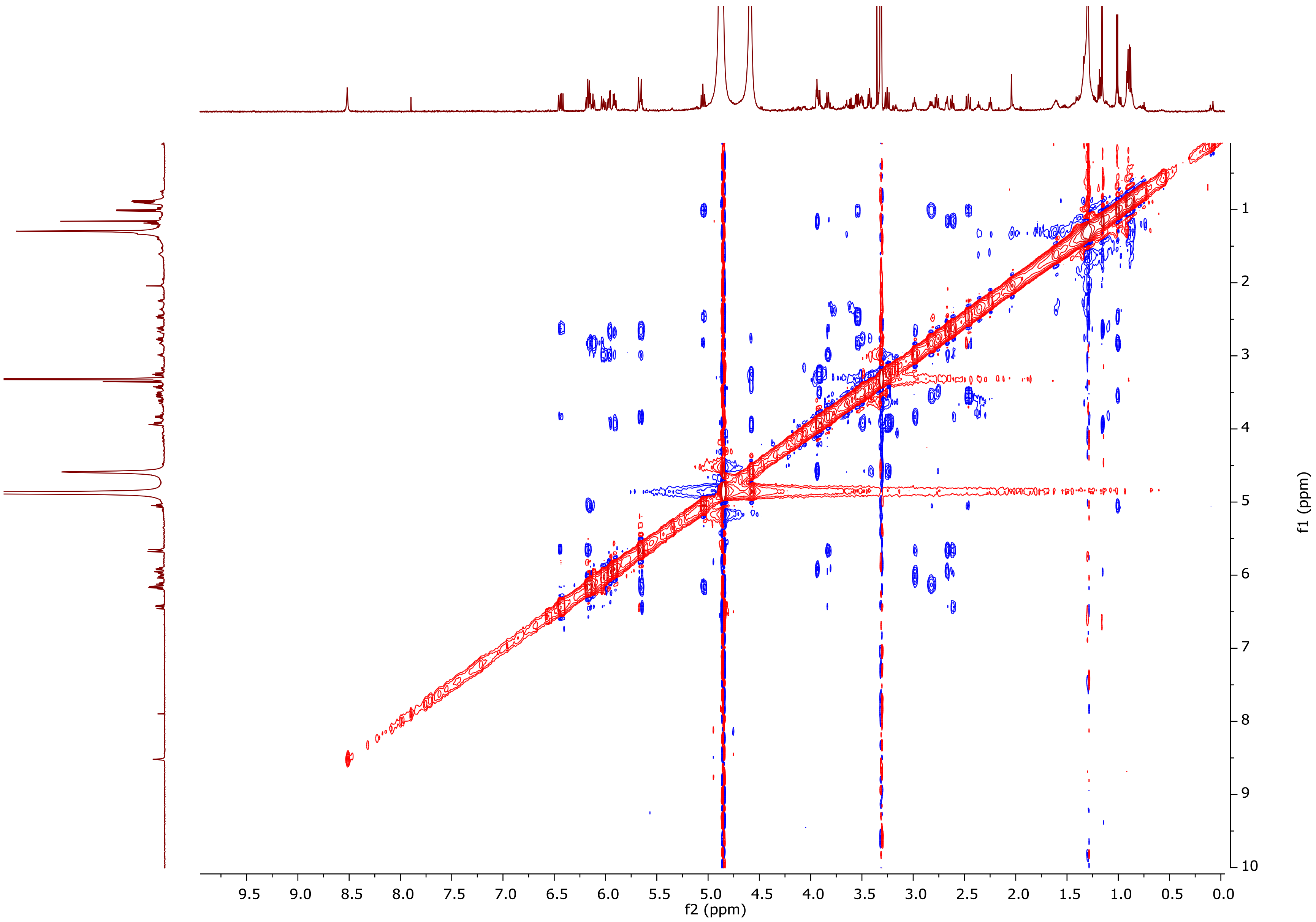

Figure S52. ROESY NMR spectrum of macrotermycin G (**4**) in CD_3OD.

Elemental Composition Report

Single Mass Analysis

Tolerance = 10.0 PPM / DBE: min = -1.5, max = 600.0
Element prediction: Off
Number of isotope peaks used for i-FIT = 3

Monoisotopic Mass, Even Electron Ions
140 formula(e) evaluated with 2 results within limits (all results (up to 1000) for each mass)
Elements Used:
C: 0-120 H: 0-250 N: 0-3 O: 1-6

Christine Beemelmanns, M39-V8-P1 University of Illnois, SCS, Mass Spectrometry Lab Q-tof UE521
Qtof_45785 29 (2.078) AM (Cen,3, 80.00, Ar,15000.0,716.46,0.70,LS 2); Sm (SG, 2x3.00); Cm (29:31) 1: TOF MS ES+
1.23e+003

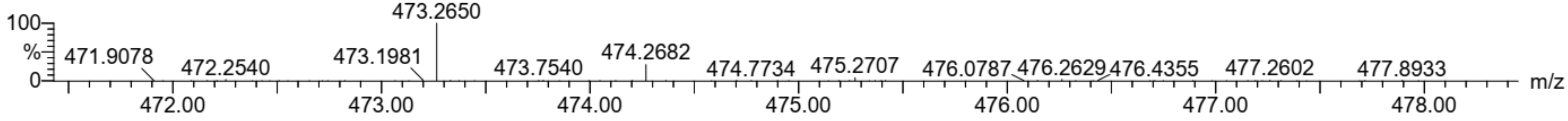

Minimum:				-1.5		
Maximum:		5.0	10.0	600.0		
Mass	Calc. Mass	mDa	PPM	DBE	i-FIT	Formula
473.2650	473.2652	-0.2	-0.4	9.5	1.3	C26 H37 N2 O6
	473.2692	-4.2	-8.9	13.5	10.2	C31 H37 O4

Figure S53. HRMS analysis of macrotermycin G (4)

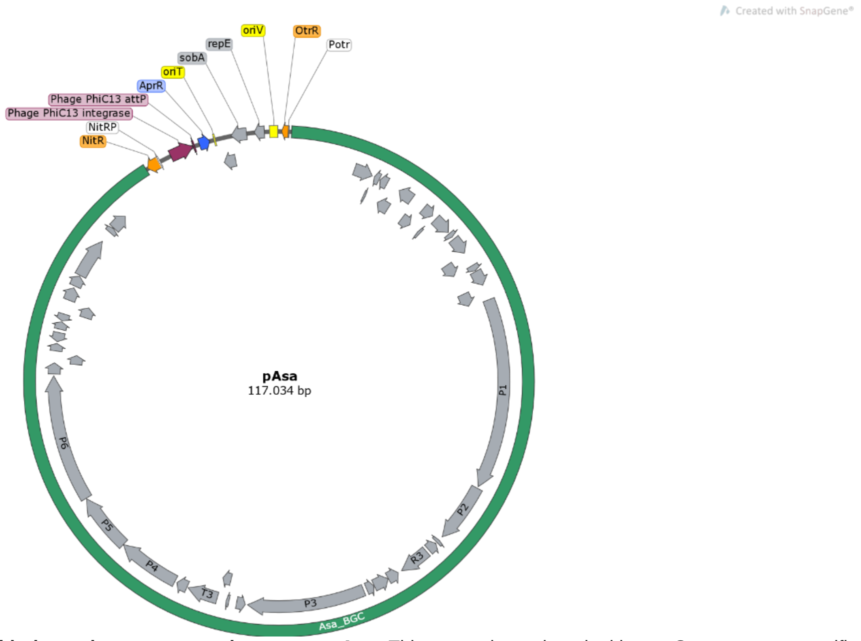

Figure S54. Vector map of the dual-promotor inducible heterologous expression vector pAsa. This vector is equipped with two Streptomyces-specific, inducible promoters flanking the inserted *asa* BGC. Potr, upstream of the cluster, can be activated by oxytetracycline dihydrate (OXT) and NitRP, downstream of the cluster, can be activated by ε -caprolactam (ε-CL). In addition, this vector contains an apramycin resistance gene (AprR) for selection in *E. coli* and *Streptomyces*, an oriT for conjugational transfer and phage φ-13 attP site and integrase for integration in the *Streptomyces* genome.

Table S12. Cas9 gRNAs.

Cluster	Cas9 Cut Site	Guide RNA Sequence	PAM
asaBGC-left	Left side	GTGGAACTCGATGAGCTGAC	CGG
asaBGC-right	Right side	GATGTAGTACACGTAGTCGG	TGG

Table S13. Primers used in this study.

Name	Sequence (5' – 3')	Purpose
L-Asa_fwd3	CACTCGCGAACGCAGAAAG	Colony PCR primer for confirmation of insertion of pAsa
L-Asa_rev3	CAGGTGCAGTGGAACTCGAT	Colony PCR primer for confirmation of insertion of pAsa
R-Asa_fwd2	GCATGAAGCTGGTCTGGGTG	Colony PCR primer for confirmation of insertion of pAsa
R-Asa_rev2	ATCCGTAACCTCCGACTCGA	Colony PCR primer for confirmation of insertion of pAsa

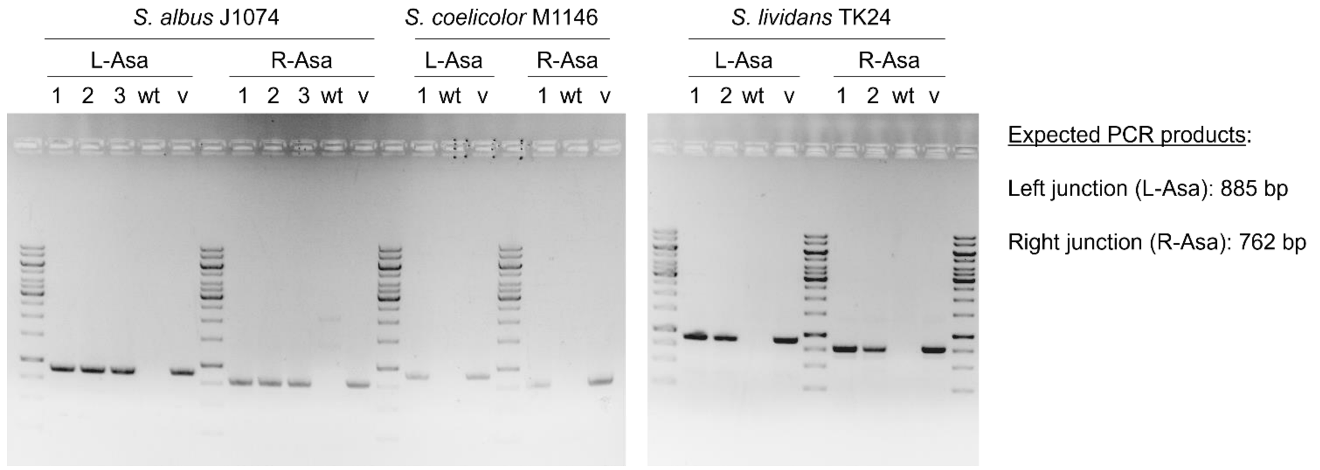

Figure S55. Verification of *asa* BGC insertion mutants. PCR amplifying left and right BAC-BGC junctions to verify the introduction of the whole BGC in Streptomyces hosts. PCR reactions (7 µl) were applied into a 1% agarose gel and electrophoresis was conducted for 45 min at 100V. As size reference Generuler^{TM} 1kb DNA ladder (Thermo Fisher Scientific Inc., USA) was used. Wildtype DNA was used as negative control (wt) and isolated vector as positive control (v).

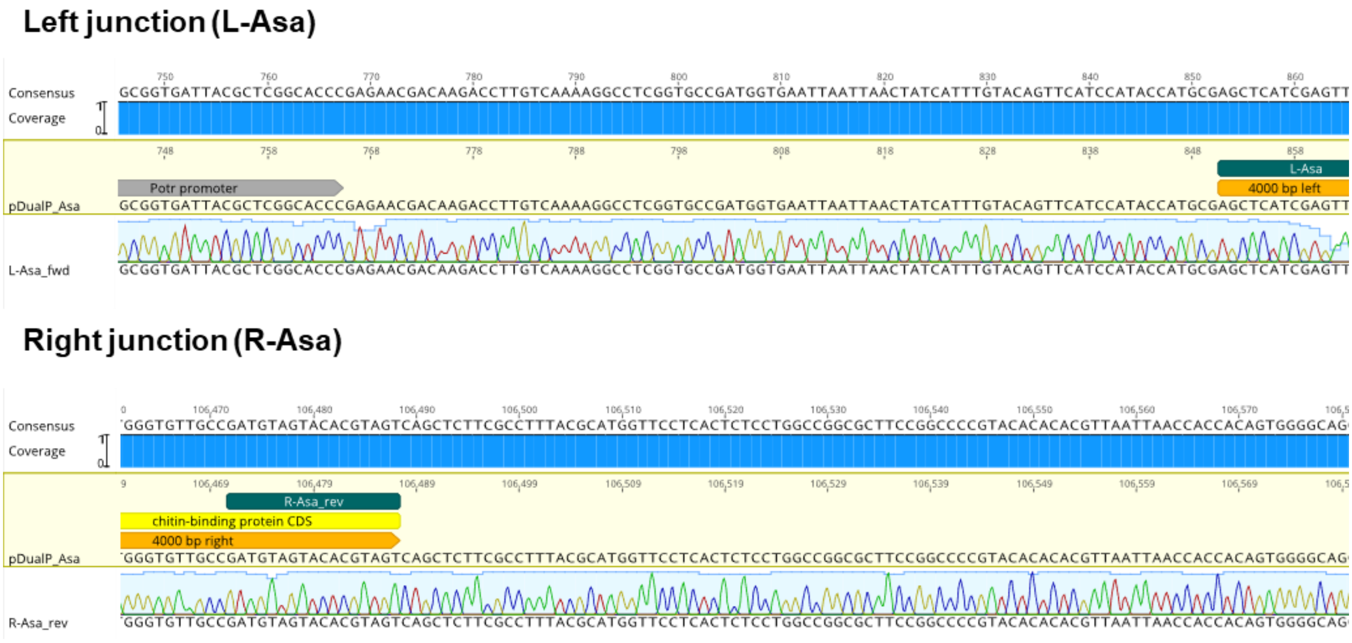

Figure S56. Verification of BAC vector insertion in *Streptomyces* host genomes. Sanger sequencing of each vector-BGC junction to confirm the insertion of the entire *asa* BGC into the *Streptomyces* genomes (exemplary for strain *S. albus*::pAsa clo).

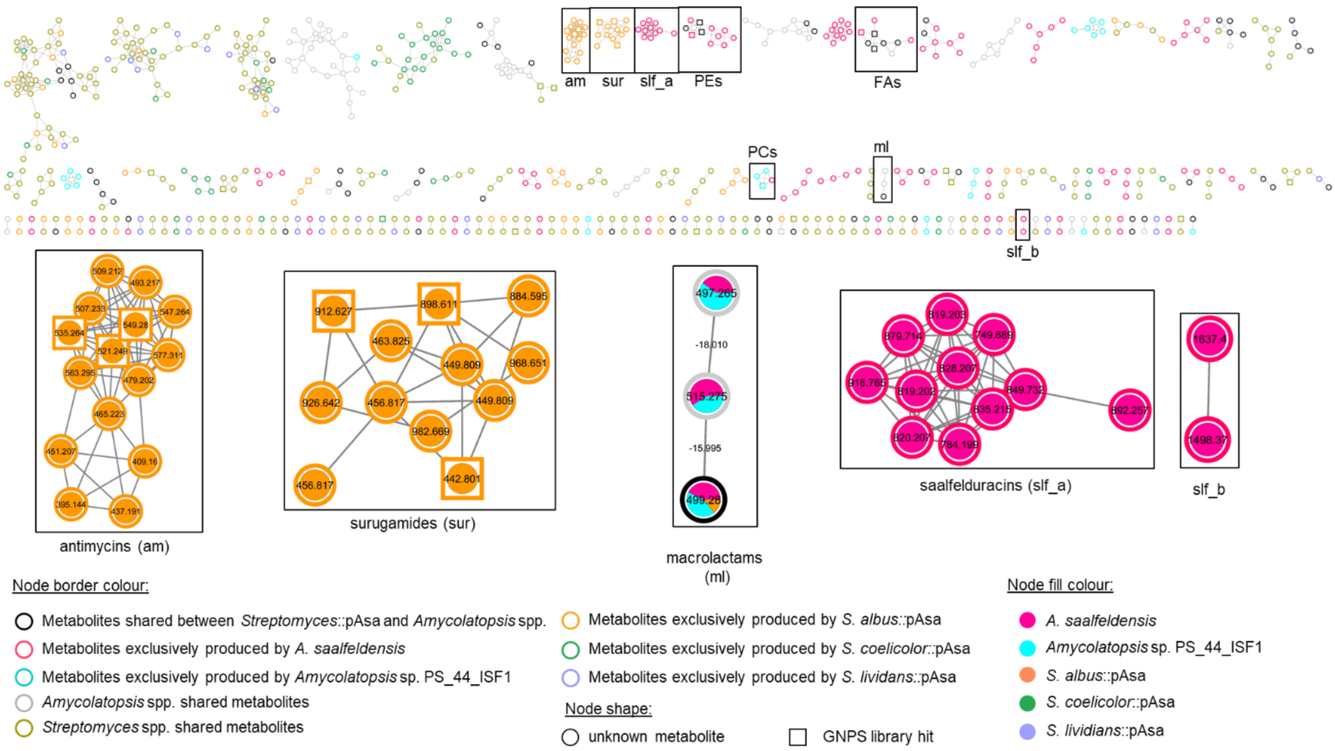

Figure S57. GNPS cluster for the comparison of *Streptomyces* heterologous hosts with *Amycolatopsis saalfeldensis* and ISF1 known to produce macrolactams (cosine score: 0.8). *Streptomyces* were extracted with EtOAc.

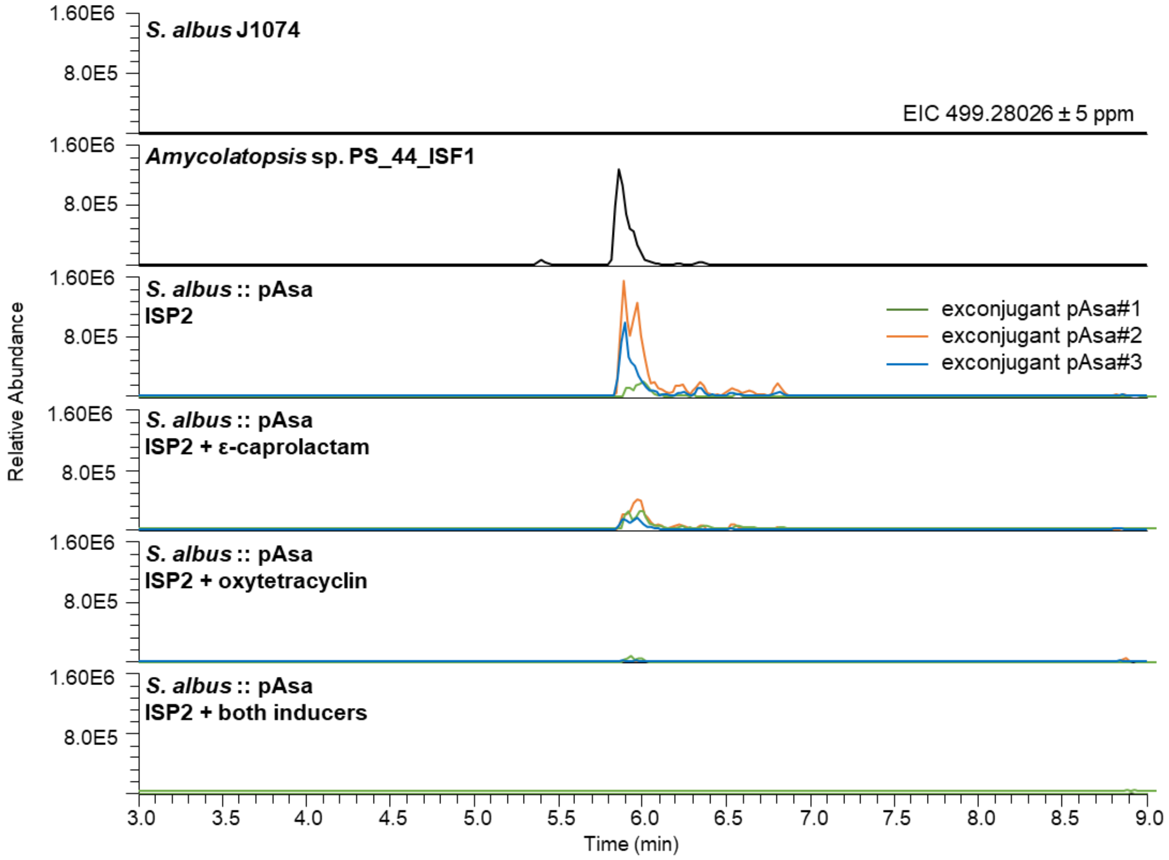

Figure S58. Comparison of macrolactam production in heterologous *Streptomyces albus* exconjugants. Extracted ion chromatograms (EIC 499.28026 ± 5 ppm) of *S. albus* exconjugants carrying the *asa* BGC cultivated on ISP2 with different inducers. The 3 different exconjugants are overlayed. *S. albus* wildtype served as negative control and *Amycolatopsis* sp. PS_44_ISF1 as positive control.

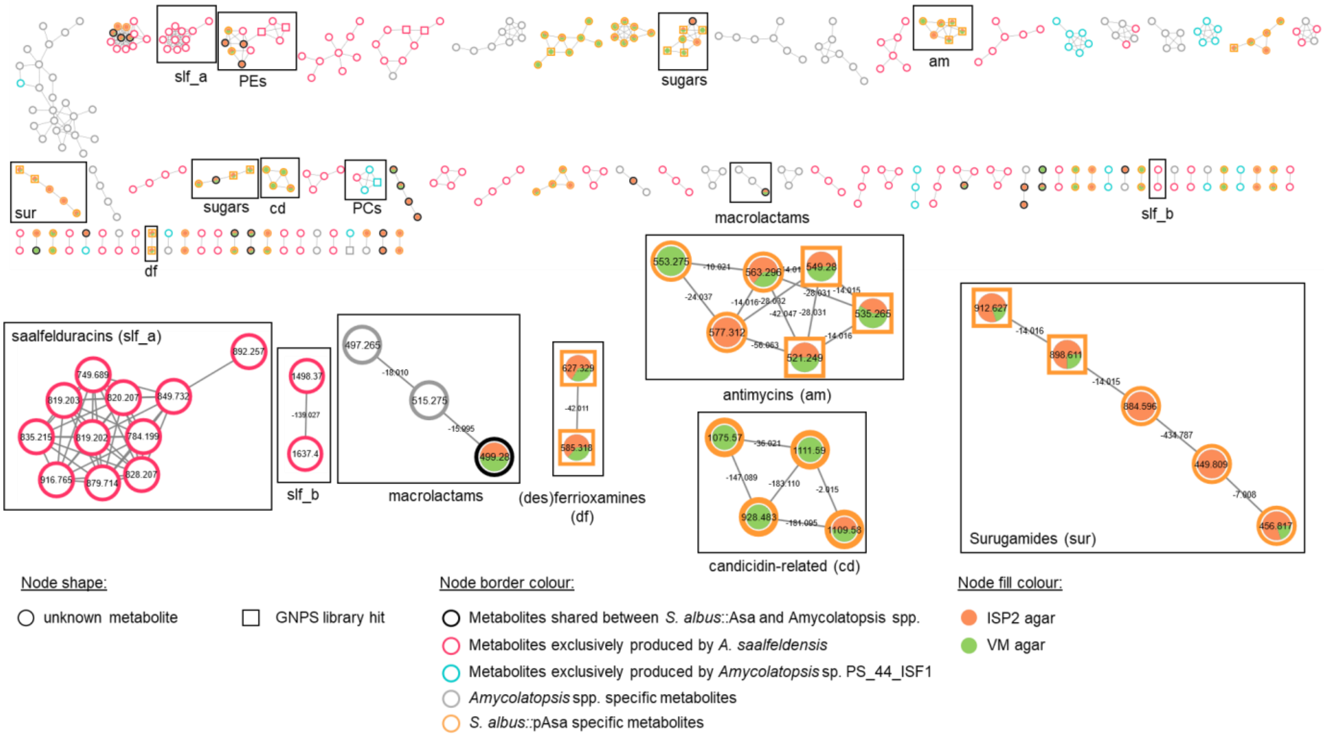

Figure S59. Metabolic comparison of *S. albus*::pAsa on VM and ISP2. *Amycolatopsis saalfeldensis* and ISF1 were added as control for macrolactam production (cosine score 0.8).

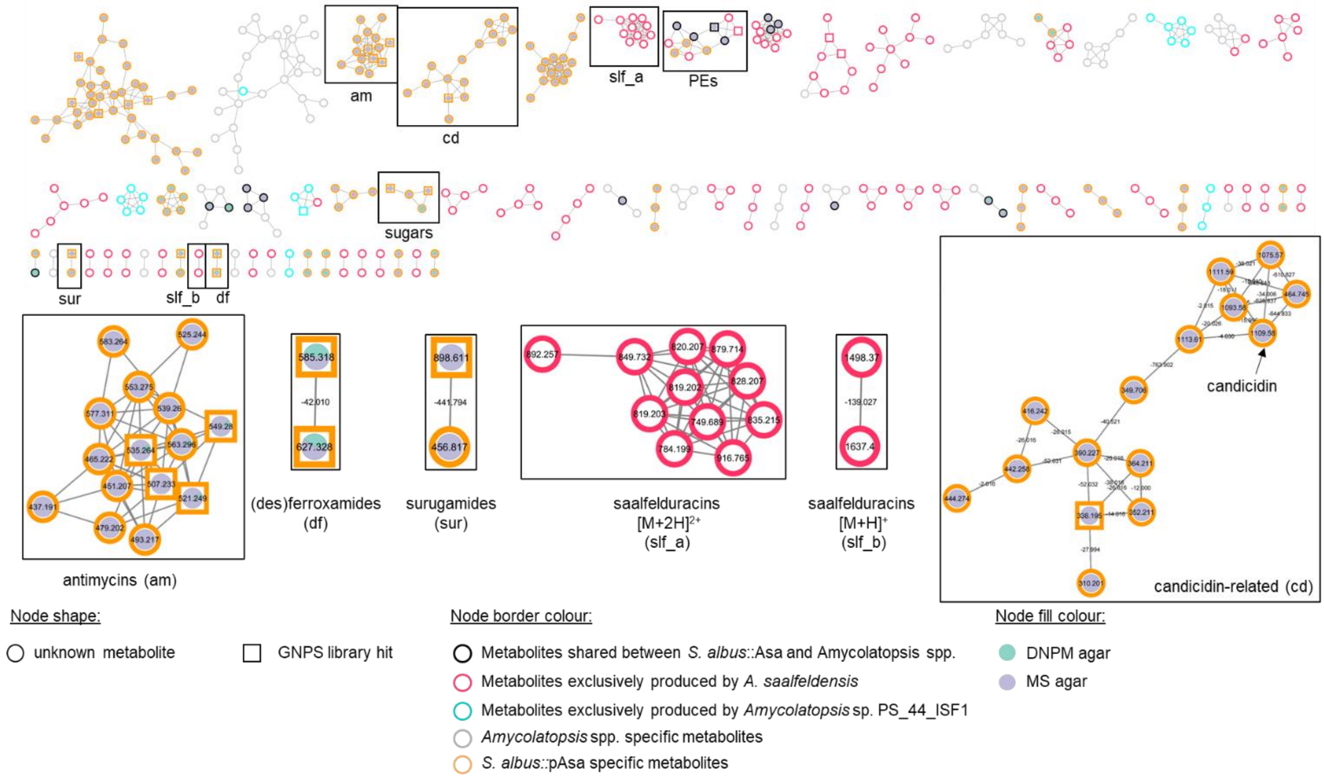

Figure S60. Metabolic comparison of *S. albus*::pAsa on MS and DNPM. *Amycolatopsis saalfeldensis* and ISF1 were added as control for macrolactam production (cosine score: 0.8).

Supplementary References

[1] Gilchrist, C. L. M.; Booth, T. J.; van Wersch, B.; van Grieken, L.; Medema, M. H.; Chooi, Y. H., *Bioinform Adv* **2021,** *1*, vbab016, 10.1093/bioadv/vbab016.
[2] Caffrey, P., *ChemBioChem* **2003,** *4*, 654-7, https://doi.org/10.1002/cbic.200300581.
[3] Yeo, W. L.; Heng, E.; Tan, L. L.; Lim, Y. W.; Ching, K. C.; Tsai, D. J.; Jhang, Y. W.; Lauderdale, T. L.; Shia, K. S.; Zhao, H.; Ang, E. L.; Zhang, M. M.; Lim, Y. H.; Wong, F. T., *Microb Cell Fact* **2020,** *19*, 3, 10.1186/s12934-019-1274-y.
[4] Beemelmanns, C.; Ramadhar, T. R.; Kim, K. H.; Klassen, J. L.; Cao, S.; Wyche, T. P.; Hou, Y.; Poulsen, M.; Bugni, T. S.; Currie, C. R.; Clardy, J., *Org Lett* **2017,** *19*, 1000-3, 10.1021/acs.orglett.6b03831.
[5] Carlsohn, M. R.; Groth, I.; Tan, G. Y. A.; Schütze, B.; Saluz, H.-P.; Munder, T.; Yang, J.; Wink, J.; Goodfellow, M., *International Journal of Systematic and Evolutionary Microbiology* **2007,** *57*, 1640-6, https://doi.org/10.1099/ijs.0.64903-0.
[6] Guo, H.; Kreuzenbeck, N. B.; Otani, S.; Garcia-Altares, M.; Dahse, H.-M.; Weigel, C.; Aanen, D. K.; Hertweck, C.; Poulsen, M.; Beemelmanns, C., *Organic Letters* **2016,** *18*, 3338-41, 10.1021/acs.orglett.6b01437.
[7] Zaburannyi, N.; Rabyk, M.; Ostash, B.; Fedorenko, V.; Luzhetskyy, A., *BMC Genomics* **2014,** *15*, 97, 10.1186/1471-2164-15-97.
[8] Gomez-Escribano, J. P.; Bibb, M. J., *Microbial Biotechnology* **2011,** *4*, 207-15, https://doi.org/10.1111/j.1751-7915.2010.00219.x.
[9] Kieser, T.; Foundation, J. I., *Practical Streptomyces Genetics*. John Innes Foundation: 2000.
[10] Derewacz, D. K.; Covington, B. C.; McLean, J. A.; Bachmann, B. O., ACS Chem Biol 2015, 10, 1998-2006, 10.1021/acschembio.5b00001.
[11] Wang, F.; Liigand, J.; Tian, S.; Arndt, D.; Greiner, R.; Wishart, D. S., *Analytical Chemistry* **2021,** *93*, 11692-700, 10.1021/acs.analchem.1c01465.
[12] Beemelmanns, C.; Ramadhar, T. R.; Kim, K. H.; Klassen, J. L.; Cao, S.; Wyche, T. P.; Hou, Y.; Poulsen, M.; Bugni, T. S.; Currie, C. R.; Clardy, J., *Org Lett* **2017,** *19*, 1000-3, 10.1021/acs.orglett.6b03831
