# Supplementary material for: Genome mining for macrolactam-encoding gene clusters allowed for the network-guided isolation of β-amino acid-containing cyclic derivatives and heterologous production of ciromicin A
Source: Commun Chem. 2023 Nov 20;6:257. doi: 10.1038/s42004-023-01034-w (PMC10662134; doi:10.1038/s42004-023-01034-w)
Supplement: Supplementary file 3 — Description of Additional Supplementary Files [file 42004_2023_1034_MOESM3_ESM.pdf]

Description of Additional Supplementary Files

File name: Supplementary Data 1

Description: Abundance and structural diversity of macrolactams

File name: Supplementary Data 2

Description: cblaster output
